# Bacterial communities 16S rDNA fingerprinting as a potential tracing tool for cultured seabass *Dicentrarchus labrax*

**DOI:** 10.1038/s41598-017-11552-y

**Published:** 2017-09-19

**Authors:** Tânia Pimentel, Joana Marcelino, Fernando Ricardo, Amadeu M. V. M. Soares, Ricardo Calado

**Affiliations:** 1 Departamento de Biologia & CESAM & ECOMARE, Universidade de Aveiro, Campus Universitário de Santiago, 3810-193 Aveiro, Portugal; 2 Instituto Universitário de Lisboa (ISCTE-IUL), DINÂMIA’CET, Avenida das Forças Armadas, 1649-026 Lisbon, Portugal

## Abstract

Traceability of seafood has become crucial with market globalization and consumer’s awareness. The present study used PCR-DGGE and 454 pyrosequencing to assess if bacterial communities fingerprint associated to seabass (*Dicentrarchus labrax*) skin mucus can be used to discriminate the geographic origin of fishes cultured in three semi-intensive fish farms. PCR-DGGE and pyrosequencing results were congruent and suggested that this molecular approach has the potential to trace fish farms with a spatial resolution <500 m. Pyrosequencing results provided a detailed insight into the bacterial community composition of seabass skin mucus and revealed the existence of a core of bacterial communities within family Pseudomonadaceae and Rhodobacteraceae. This approach also allowed to recognized key OTUs that are potentially relevant to discriminate the geographic origin of the fish being surveyed. Overall, the present study increased our knowledge on farmed seabass microbiome and demonstrated that specific and unique bacterial taxa can act as natural signatures that allow us to trace fish to its respective geographic origin. Our study provides valuable clues that should be more investigated in future studies as a way to fulfill current traceability needs in the global trade of seafood.

## Introduction

European seabass (*Dicentrarchus labrax*, L.) is one of the most commonly farmed marine fish species accounted for 134 538 tons in 2014^1^. Seafood production prompts trade and safety challenges^2–4^. As European seabass is mainly marketed whole and fresh, its perishable nature and associated risks are enhanced when compared to other farmed fish species (e.g., salmon or trout). The complexity of seafood supply chains is unprecedented. The increasing globalization of this activity and the recurrent food safety alerts issued have prompted a growing awareness on consumers towards these issues^5–7^. In this context, and in order to accomplish global societal needs, a global requirement for seafood traceability has emerged; consequently, seafood trade is characterized by international legislation that ensure the possibility of easily tracing back the origin of traded products^2,3^, with the aquaculture sector benefiting from international standards and certification systems^8^.

Current needs on seafood traceability have driven relevant biotechnological breakthroughs that can have direct applications in the authentication or/and origin certification of seafood^3,9,10^. The study of microbial communities diversity associated to food products, as well as its linkage to a particular geographic origin, has already been applied in traceability issues, namely the molecular approach employing polymerase chain reaction denaturing gradient gel electrophoresis (PCR-DGGE)^11–16^. This molecular method relies on DNA amplification of microbial communities associated to a food product. The amplicons produced are after used as samples on the DGGE and the result is an electrophoretic profile composed of several bands that can be assumed as a genetic fingerprint of microbial communities related with the origin of any given food item^17^. PCR-DGGE is recommended as an effective biotechnological tool for seafood traceability since it can provide unique biological signatures that can be assigned to specific geographic origins^2,3^. Nonetheless, this method does not provide an in-depth representation of microbial communities composition^18^. In this way, it is necessary to employ subsequent molecular approaches based on large scale comparative analysis of 16S ribosomal RNA with the potential to profile microbial communities at a higher resolution. Next generation Sequencing (NGS), such as 454 pyrosequencing technique, allows to understand in detail the microbial composition associated with a specific community and provides a simple and cost-effective mechanism for characterizing the composition of bacterial communities^19–21^. This NGS method allows to recognize the microbial profile associated to each PCR-DGGE fingerprint and thus offers the possibility to identify taxonomically any given microbiological signature. This highly accurate and sensitive information will enable the development and implementation of better traceability systems and it is particularly relevant for aquaculture. In fact, the discrimination of the exact geographic origin of traded fish will profit both producers and consumers. In other words, while fish farms can promote their products based on safety and sustainable aquaculture practices, consequently adding value to their products, consumers willing to pay more for such premium products may be offered a better warranty to verify the claims made by producers.

Another important aspect to take into account to achieve a suitable traceability methodology using the tools described above relies on the morphological structure of fish being surveyed. Indeed, the determination of geographic origin using microbial fingerprints conditions the use of the fish matrix to be surveyed; this matrix should be more prone to evidence environment-driven shifts in its microbial communities. Therefore, and despite the few studies addressing the diversity skin microbiota of fishes^22,23^, skin mucus emerges as a preferential matrix to investigate microbial community composition. Furthermore, skin fish mucus is a biochemically complex fluid that includes a number of nutrient that favor a rich bacterial diversity, as well as several antibacterial compounds secreted by skin cells^24,25^. In this favorable microenvironment for bacteria the balance between its constituents seem to have a very important role to discriminate between commensals, symbionts or pathogenic bacterial strains, which are collectively forming the fish skin mucus microbiome^25^. The present study aimed to evaluate if bacterial communities fingerprint of seabass skin mucus can be used to successfully discriminate the origin of specimens produced in three semi-intensive fish farms using a PCR-DGGE and 454 pyrosequencing approach.

## Material and Methods

### Study site and sampling preparation

**Figure 1:**
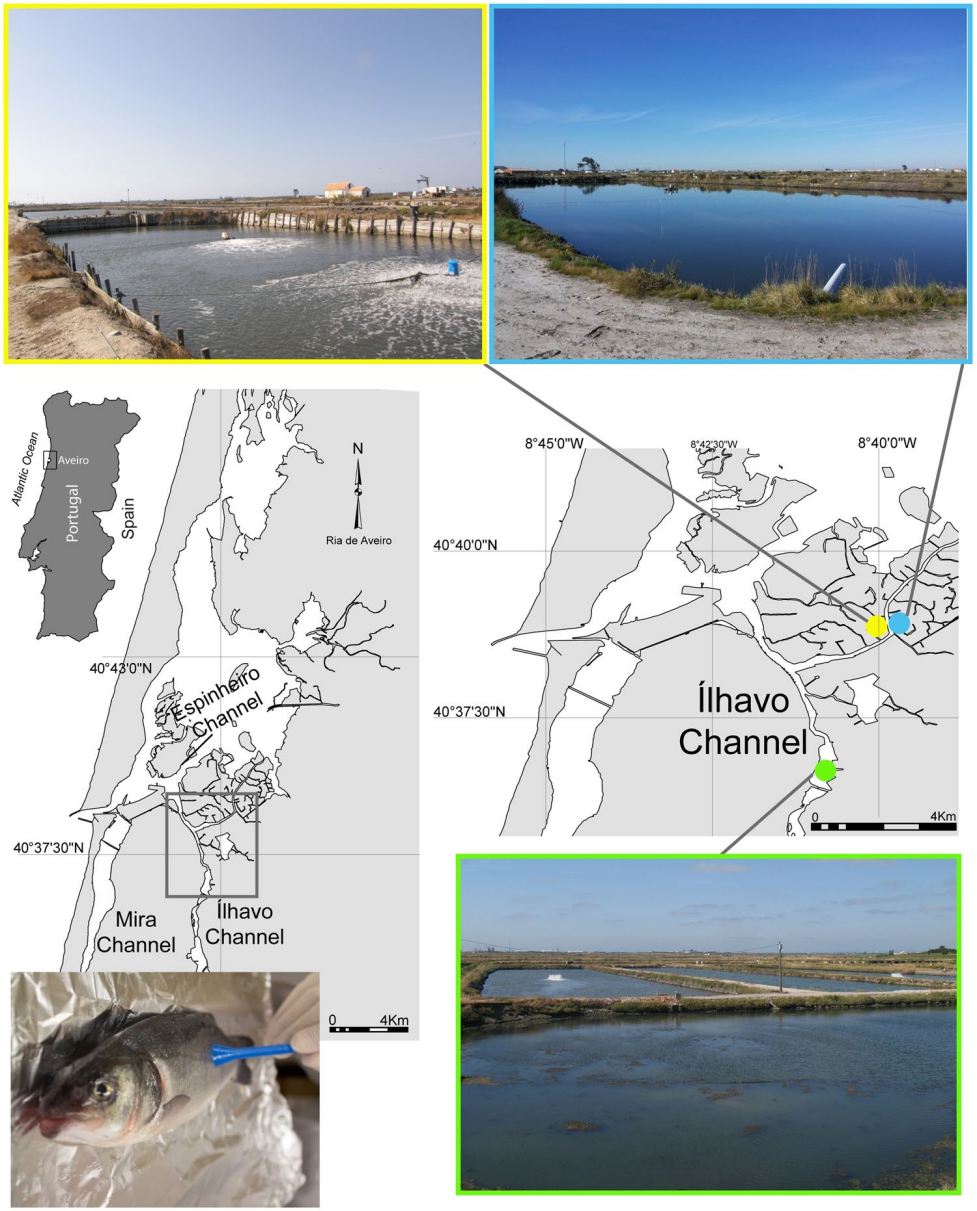
Map of Portugal, with Ria de Aveiro ecosystem showing the geographical location of three sampling semi-intensive fish farms:  - Farm A,  - Farm B and  - Farm C. The map was created using the software ArcGIS v10.2.2.

The present study was performed in three semi-intensive fish farms producing seabass (*Dicentrarchus labrax*) in earth ponds located in Ria de Aveiro - Portugal (Fig. 1) during the peak period of commercial transactions (July). Five individual fish (average weight and length: 600 ± 50 g and 350 ± 20 mm, respectively) from each farm were harvested and stored individually in separated sterile plastic bags. Sampled seabasses were immediately transported to the laboratory in refrigerated polystyrene box. In the laboratory, four samples of skin mucus were collected from each fish along the fish lateral line. This process can be briefly described as follows: skin mucus was scraped along the lateral line of the fish with sterile spatulas and samples of 500 mg were transferred into screw cap microcentrifuge tubes containing 1 mL of PBS (Phosphate-Saline Solution at pH 7.4). All samples were centrifuged at 16 000 g at 4 °C for 16 min to pellet microbial biomass. After decanting the supernatant, microbial biomass pellets were stored immediately at −20 °C for posterior DNA extraction.

### DNA extraction and PCR-DGGE bacterial community fingerprint

DNA extraction included an initial mechanical lysis step using FastPrep24 instrument (MP Biomedicals Europe) at 6.5 m s^−1^ for 90 s and afterwards was accomplished using E.Z.N.A Soil DNA Extraction kit (Omega Bio-Tek, USA) following the manufacturer’s protocol.

The 16S rRNA gene was amplified using a nested polymerase chain reaction (PCR) approach. The 27 F and 1494 R primer set^26^ was used in the first PCR at 0.1 µM each primer. PCR reaction mixture (final volume of 25 µL) included 1 µl of template DNA, 0.08 mg/mL of BSA, 1x DreamTaq DNA polymerase Master Mix (Thermo Scientific In. US) and water nuclease-free. The amplification was carried out as follow: an initial denaturation at 94 °C for 5 min and 25 cycles of denaturation at 94 °C for 45 s, annealing at 56 °C for 45 s, and extension at 72 °C for 1.5 min, followed by a final extension step at 73 °C for 10 min. For the second PCR, 1378 R and 984GC primers^27,28^ were used at 0.2 µM. PCR reaction mixture (final volume of 25 µL) included 1 µL of the product obtained in the first PCR, 4% (v/v) of acetamide, 1x DreamTaq DNA polymerase Master Mix (Thermo Scientific In. US) and water nuclease-free. The amplification was carried out as follow: an initial denaturation at 94 °C for 4 min and 30 cycles of denaturation at 95 °C for 1 min., annealing at 53 °C for 1.5 min., and extension at 72 °C for 1.5 min, followed by a final extension step at 73 °C for 7 min. The PCR reactions were conducted in a TProfessinal Thermocycler (Biometra, Goettingen, Germany). Reactions included a negative and positive control. Aliquots (5 μL) of PCR products were analyzed by electrophoresis in 1.2% (w/v) agarose gel with TAE 1 × buffer (40 mM Tris–HCL pH 7.4, 20 mM sodium acetate, 1.0 mM Na_2_-EDTA), stained with 1.5 × Gel Red (VWR) and verified using a standard DNA (100 bp ladder, Promega).

PCR products were loaded on 8% acrylamide gel with a denaturing gradient ranging from 40% to 60% where 100% denaturant correspond to 7 M urea (Sigma-Aldrich, Co., St. Louis, MO) and 40% (v/v) formamide (Sigma-Aldrich, Co., St. Louis, MO). Denaturing gradient gel electrophoresis (DGGE) was performed within TAE 1 × buffer at 60 °C applying 160 V during 16 hours, using a DCode™ Universal Mutation Detection System (Bio-Rad, Hercules, CA, USA). Gels were silver-stained according to Byun *et al*.^29^ and images were acquired by the Molecular Imager® Gel Doc™ XR + System with Image Lab™ Software (Bio-Rad, Hercules, CA, USA).

### Barcoded pyrosequencing

A barcoded pyrosequencing approach was used for a preliminary study of bacterial communities composition associated to each geographical origin under analysis. Prior to pyrosequencing, DNA from all twenty replicates of each sampling site were combined in equimolar ratios, forming one DNA library per fish farm.

The V3-V4 hypervariable region of bacterial 16S rRNA gene was amplified using barcoded fusion primer Forward (5′-ACTCCTACGGGAGGCAGCAG-3′) and Reverse primer (5′-TACNVRRGTHTCTAATYC-3′)^30^ attached to the 454 A and B adaptors, respectively. The amplicons were purified and subsequently quantified by fluorimetry with PicoGreen dsDNA quantitation kit (Invitrogen, CA, USA), pooled at equimolar concentrations and sequenced in the B direction with GS 454 FLX Titanium chemistry, according to manufacturer’s instructions (Roche, 454 Life Sciences, Brandford, CT, USA) at Biocant (Cantanhede, Portugal).

### Statistical analysis and bioinformatics

DGGE band patterns were analyzed using BioNumerics software (version 6.6, Applied Maths, Ghent, Belgium). The program generated a matrix that combined the band position and the associated band intensity. This matrix was upload to R^31^, converted into relative abundances and data were log (x + 1) transformed. Using the function metaMDS from the “vegan” package in R, with Bray-Curtis similarity coefficient, a non-metric multidimensional scaling (NMDS) was performed in order to visualized and interpreted bacterial communities affinity between sampling fish farms. The NMDS procedure was iterative and rationale supporting the choice of the truthful number of dimensions was the achievement of lower stress values (stress <0.05 provides an excellent representation; <0.10 is good; >0.20 provides a poor representation). Statistical differences (*p* ≤ 0.05) in bacterial communities among sampling sites were tested using the adonis function in “vegan”. If significant statistical differences were detected, an analysis of variance (Kruskal-Wallis test) was performed to identify which DGGE bands (bacterial communities) established the difference(s) between fish farms (*p* ≤ 0.05). Only the DGGE bands that allowed for a suitable differentiation were used on further statistical analysis. Post hoc tests (Nemenyi test) were conducted to detect which fish farms differed from each other (*p* ≤ 0.05).

The Mothur software^32^ was used to analyze the pyrosequencing data following Schloss *et al*.^19^ standard operation procedure (454 SOP). Briefly, all sequences were denoised using the Mothur’s implementation of the PyroNoise algorithm^33^. Sequences with more than two mismatch bases in the primers, one mismatched base in the barcode and homologous bases longer than 8 bp were discarded. Sequence reads were aligned and taxonomically classified with the SILVA database^34^ and subsequently chimeric sequences were removed applying the UCHIME method^35^ from Mothur software. Sequence reads were clustered into operational taxonomic units (OTUs) at a distance cutoff level of 3%. OTUs with no match in SILVA database grouped together as “unclassified”. Taxonomic assignments determined at the genus level were used in subsequent analysis, although some OTUs that could not find a match at genus level were reported at a higher taxonomical level (family or order). Sequence reads of individual OTUs were tabulated in an OTU table and afterwards uploaded to R. The raw sequences are available at NCBI Sequence Read Archive (SRA) database with access number SRP101902.

Alpha bacterial diversity, including the species richness estimators Chao1 and ACE and the community diversity indices Shannon and Simpson, were calculated with the “phyloseq” package in R. To determine which OTUs characterized each fish farms, shared and unique OTUs associated to each fish farm were represented in Venn diagrams generated in R applying the package “venndiagram”. Bacterial composition among fish farms was visualized in heat maps with the associated dendrograms produced at family and genus level with the package “NMF” in R. Family/genus displaying a relative abundance >1% in at least one fish farm were considered in the analysis.

## Results

**Figure 2:**
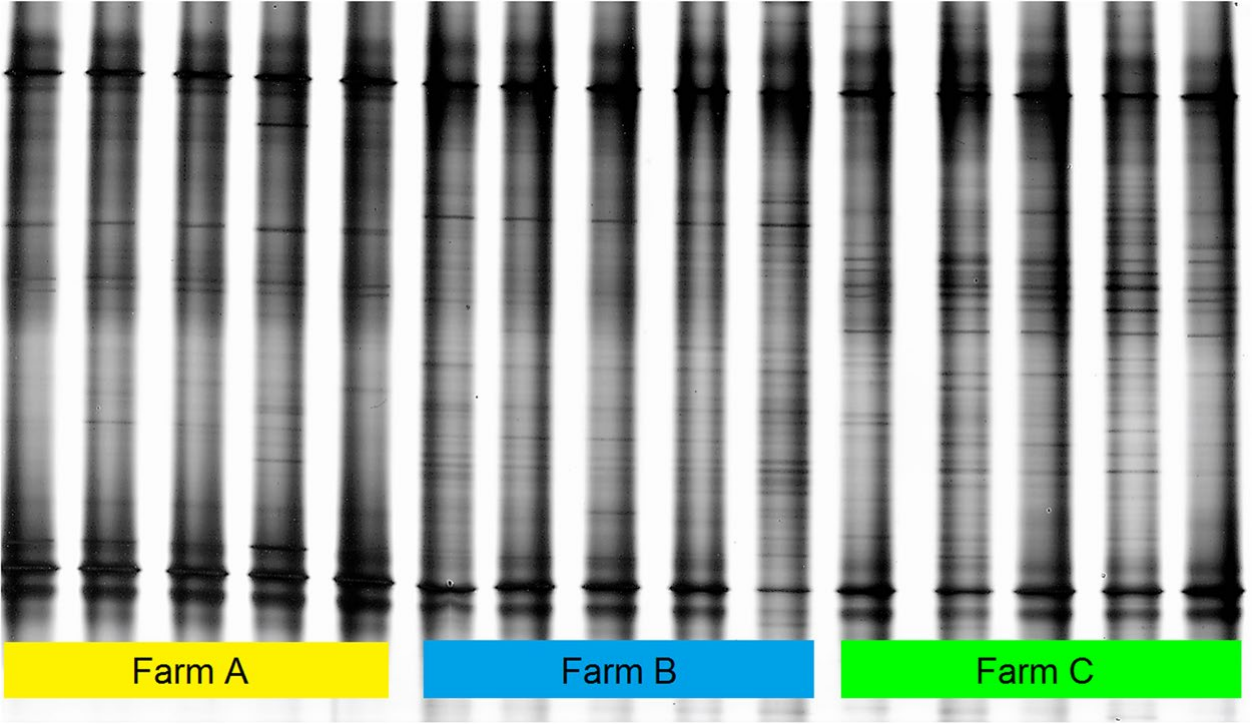
Polymerase chain reaction denaturing gradient gel electrophoresis (PCR-DGGE) banding profiles for bacterial communities in fish mucus from three semi-intensive fish farms: Farm A, Farm B and Farm C.

**Figure 3:**
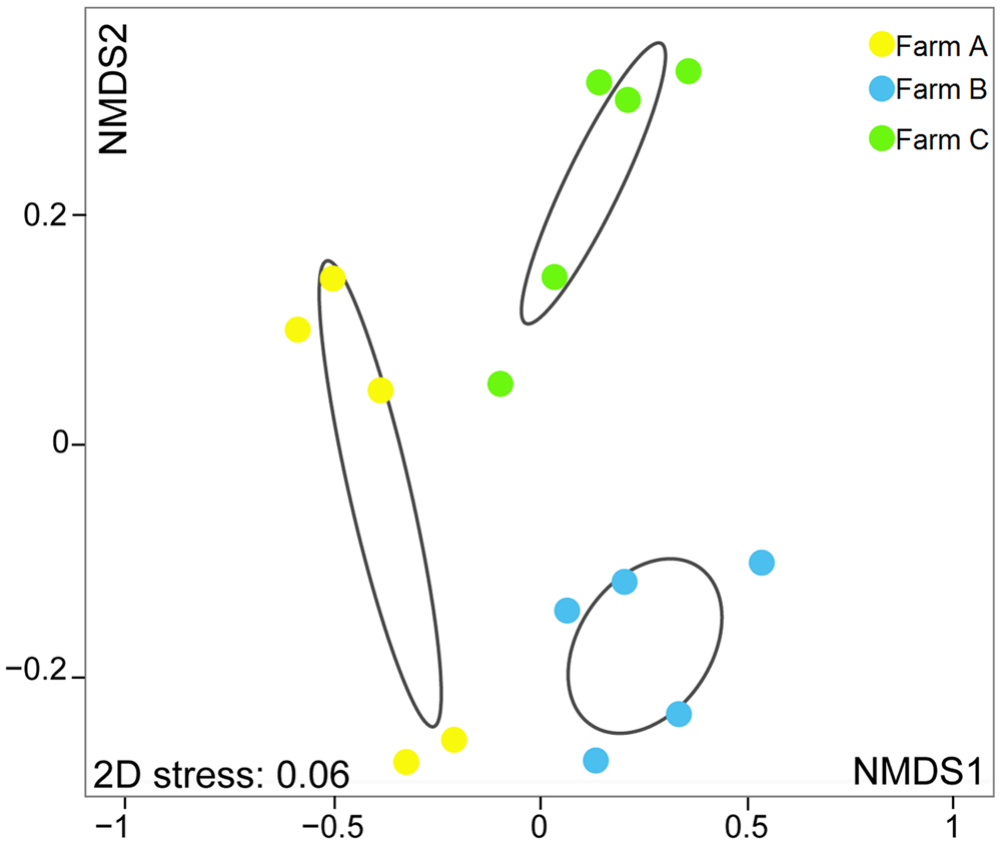
Non-metric multidimensional scaling (NMDS) ordination of bacterial DGGE fingerprint, grouped according to three semi-intensive fish farms: Farm A, Farm B and Farm C.

The PCR-DGGE patterns of bacterial communities revealed different band profiles among fish from distinct fish farms. This approach also denoted a high reproducibility between fish sampled in the location, as a very similar overall pattern was recorded between replicates (Fig. 2). NMDS analysis revealed the three fish farms clearly separated in a three dimensional space, with a stress value of 0.06 (Fig. 3). Bacterial communities from the PCR-DGGE profile were significantly different (*p* < 0.001, R^2^ = 0.665) among the three geographic locations under study. Additional analysis using the Kruskal-Wallis test allowed to identify 23 DGGE bands with significant differences (*p* ≤ 0.05) and further investigation with Nemenyi test revealed (95% confidence limit) that the difference level between the bacterial communities profile of fish farms A and B was 60.9%, while between fish farms A and C was 43.5% and fish farms B and C 21.7% (Table S1 on supplementary information). These results support the NMDS representation which exhibits farm A as the most dissimilar in terms of bacterial communities displayed on the PCR-DGGE profile.

**Figure 4:**
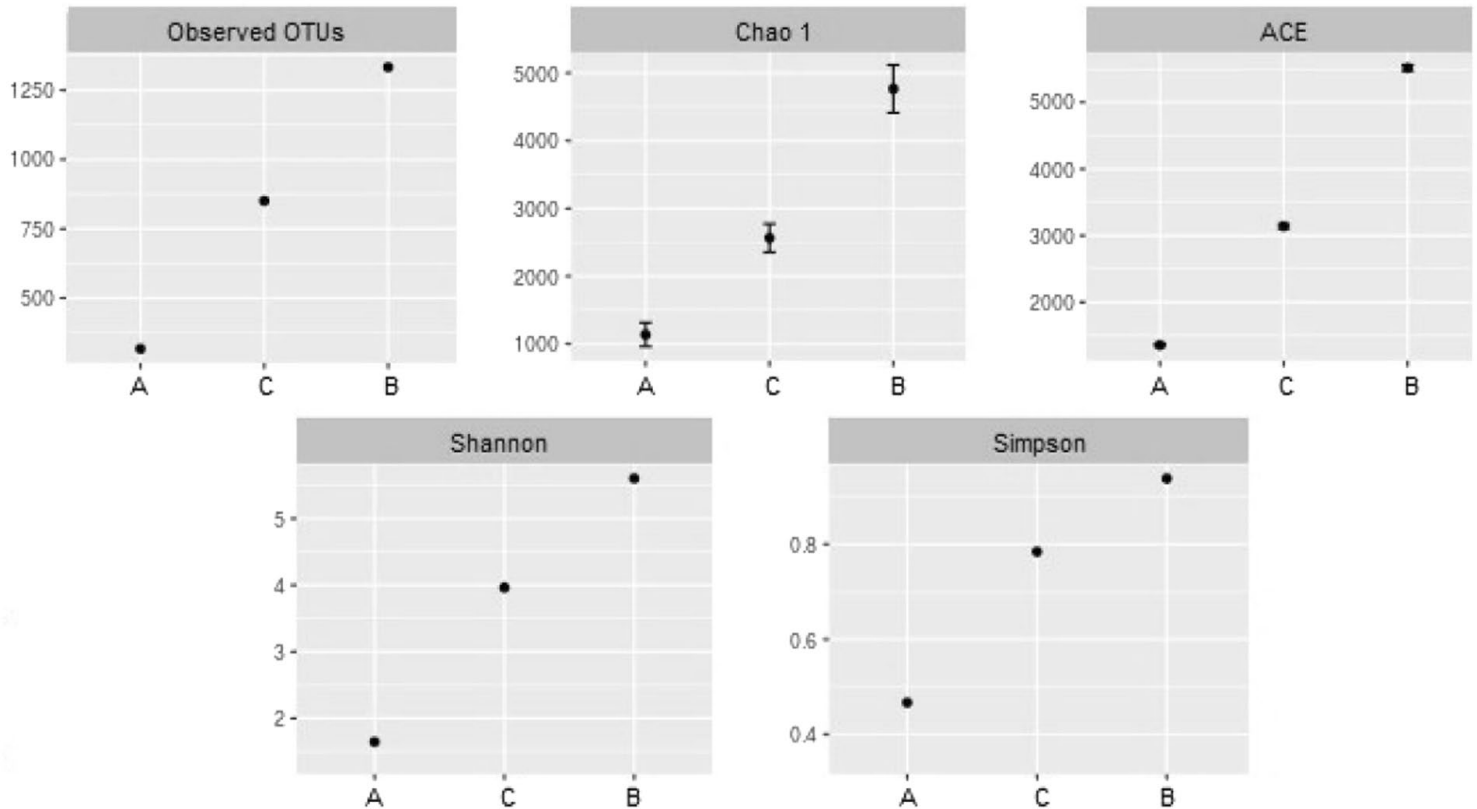
Alpha bacterial diversity based on 454 pyrosequencing data in fish mucus from three semi-intensive fish farms: Farm A, Farm B and Farm C.

The 454 pyrosequencing analysis yielded 4344, 3120 and 2844 sequence reads for fish farms A, B and C, respectively. These sequences were assigned to 1467 OTUs with a 3% level of genetic dissimilarity (Table S2 on supplementary information). The richness estimators Chao1 and ACE, as well Shannon’s and Simpson’s diversity indices, indicated that fish farm A was the location exhibiting the lowest bacterial taxonomic diversity, in opposition to fish farm B which displayed the highest values (Fig. 4). However, all the diversity parameters calculated were considerably higher than the observed OTUs numbers.

**Figure 5:**
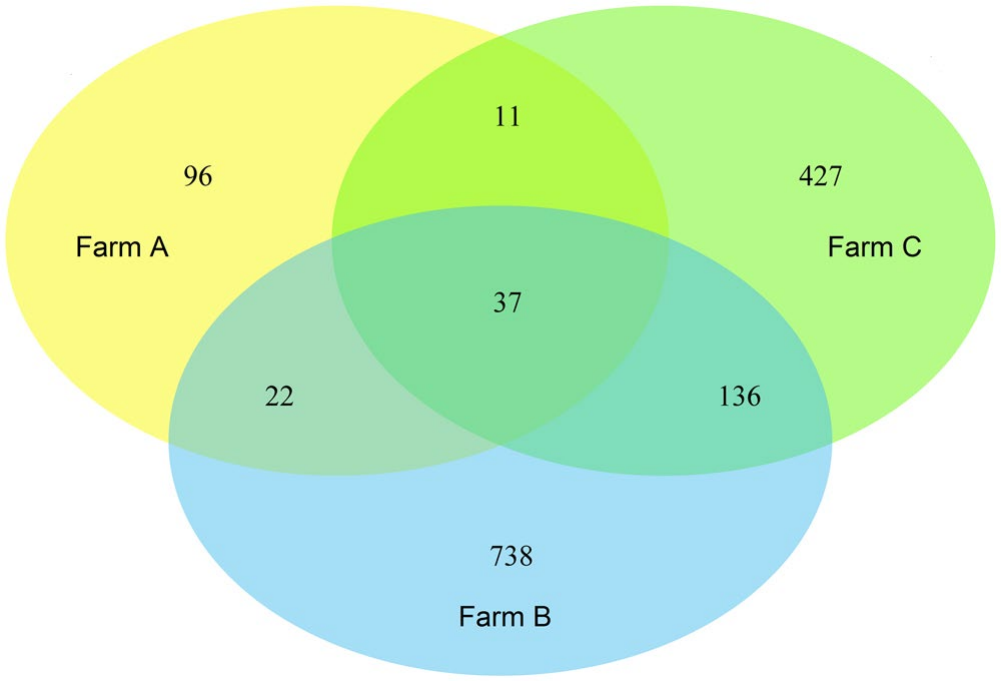
Venn diagram of shared and unique operational taxonomic units (OTUs) in fish mucus among three semi-intensive fish farms: Farm A, Farm B and Farm C.

Shared and unique OTUs between the different geographic locations can be visualized in the Venn diagram (Fig. 5), with fish farm A and B being clearly distinct. Fish farm A displayed the lowest number of unique OTUs -96- and also the lowermost amount of shared OTUs -70. In opposition, fish farm B presented the highest number of unique OTUs -738- and also the uppermost amount of shared OTUs 195. Among the three sampled locations only 37 OTUs were shared between them, although they were linked to 63% of the total number of sequence reads recorded in the study.

**Figure 6:**
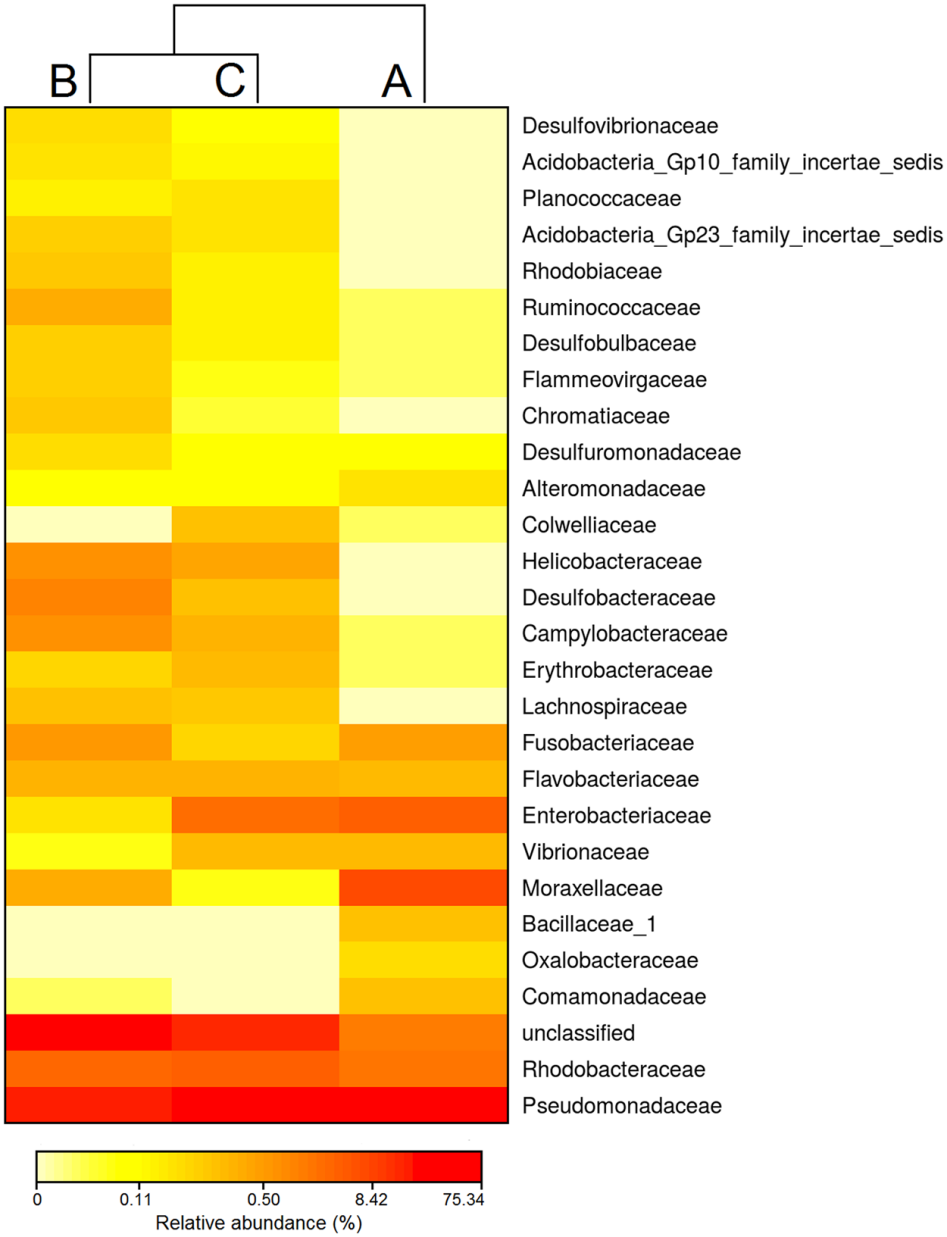
Heat map showing log(x + 1) transformed relative abundance values at family taxonomic level in three semi-intensive fish farms: Farm A, Farm B and Farm C.

**Figure 7:**
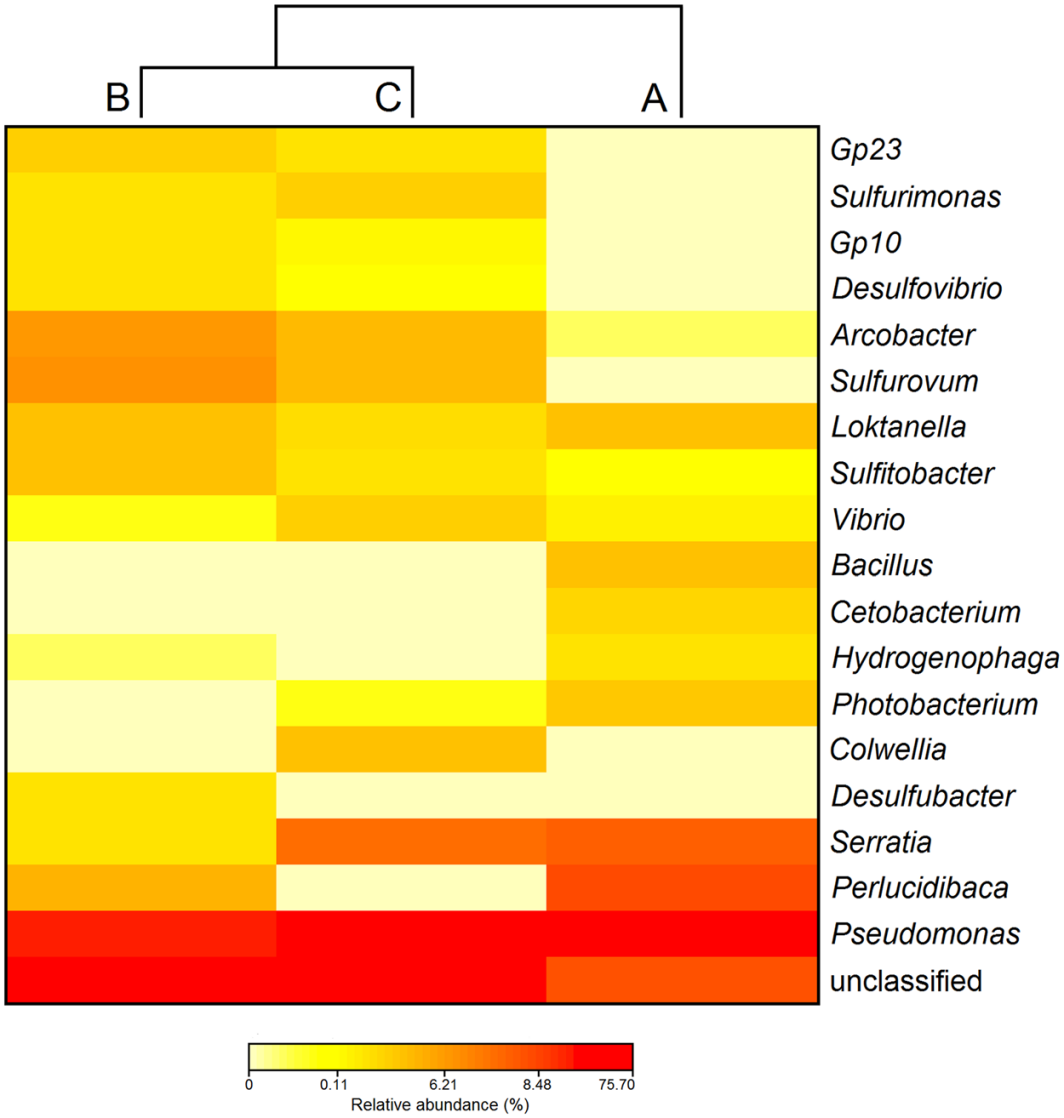
Heat map showing log(x + 1) transformed relative bacterial abundance values at genus taxonomic level in three semi-intensive fish farms: Farm A, Farm B and Farm C.

The relative bacterial community abundance, at family level, for each fish farm is presented in Fig. 6. Among the three sampling locations the most dominant and shared families were Pseudomonadaceae and Rhodobacteraceae, both belonging to phylum Proteobacteria. This core of dominant families represented 78%, 33%, and 57% of the total number of sequence reads in fish farms A, B and C, respectively. The dendrogram showed fish farms B and C as the most linked in abundance of bacterial composition. Concerning fish farm A, it was the most dissimilar one, even displaying two families not recorded in the other two fish farms: Bacillaceae and Oxalobacteraceae (3% and 2% of total abundance, respectively). The number of families shared between fish farm B and C was high, but the analysis of their bacterial community abundance at genus level (Fig. 7) allowed to discriminate fish farm B by the exclusive presence of genus *Desulfobacter* (4%) and fish farm C by the presence of genus *Colwellia* (7%). As already revealed by the approach employing a taxonomic resolution at family level, the analysis of bacterial composition at a lower taxonomic level (genus) showed an identical clustering dendrogram, with fish farm B and C being more closely related and fish farm A being the most distant (Fig. 7). Two genera were exclusively present in fish farm A, *Bacillus* and *Cetobacterium* (3% and 2% of total abundance, respectively). At genus level, *Pseudomonas* was the one most shared at the core of bacterial composition, representing 76%, 27%, and 49% of the total number of sequence reads recorded for fish farms A, B and C, respectively.

## Discussion

The present study investigated the bacterial communities fingerprint associated to seabass skin mucus in three semi-intensive fish farms using a PCR-DGGE and 454 pyrosequencing as an approach to discriminate their geographic origin. The PCR-DGGE clearly discriminated the geographic origin of sampled fish, with the effect promoted by the predictive factor fish-farm exceeding inter-individual microbial variability (Fig. 3). Indeed, it was possible to discriminate the fish farm of origin even for seabasses cultured in farms A and B, which were located solely 500 m apart. Thus, PCR-DGGE is a suitable approach to establish a bacterial fingerprint from a given geographic region prior to decide the need to gain further information on which bacterial taxa contribute to discriminate between different locations^36,37^.

Pyrosequencing data supported the findings recorded with the PCR-DGGE approach, allowing to discriminate between fish farms and confirming the existence of a core of bacterial communities present in the mucus of all specimens surveyed. In line with PCR-DGGE results, farm A stands apart from the other farms by displaying the lowest bacterial diversity (Fig. 4), as well the lowest number of shared OTUs (Fig. 5).

The richness estimators employed in the present study (Chao1 and ACE) predicted that a higher number of OTUs was likely to be present in the bacterial communities that were surveyed. Nonetheless, this finding does not invalidate the clear geographic discrimination achieved; an increase in the number of OTUs present in the fish mucus (e.g., by employing a higher sampling effort) will not erase the differences already recorded among fish farms, it will likely enhance their discrimination. Moreover, it should be noted that the highest diversity associated with fish farms B and C can be linked to the presence of more stable and resilient bacterial communities. Higher bacterial diversity is often positively correlated to microbial function stability and negatively correlated with susceptibility to pathogen invasion^38–40^.

Prior to an in depth discussion on the relevance of some of the bacterial taxa recorded in the present study, it must be highlighted that next generation sequencing technologies are yet to be routinely employed to study the microbiome associated with fish mucus^41^. Moreover, there is still a lack of standardize procedures (e.g., on sequence methodologies, use of DNA databases and pipelines to retrieve a reliable classification of bacteria taxonomy) to allow a reliable comparison of results between studies. Therefore, comparisons and generalizations on this topic must be performed with due care to avoid potential mistakes. The most abundant bacterial family in our study was Pseudomonadaceae (Fig. 6), being present in all fish farms surveyed; genus *Pseudomonas* was the most well represented (Fig. 7), with these results being in line with those from previous studies^22,40,42,43^. This results agree with those that have already specifically surveyed seabass mucus^23^ and thus we can consider that these bacteria are intrinsic components of the fish microbiome. The abundance of Rhodobacteraceae in this study has not been previously reported. However, members of this family are recognized by their potential probiotic properties, including the production of antibacterial compounds against members of family Vibrionaceae^44,45^. Whether the presence of this bacterial family is the result of the “more natural” farming practices employed in the semi-intensive production of European sea bass in earth ponds, or is supplied through the pelleted diet provided, remains an open issue that is certainly worth investigating.

The present study allowed to recognize key OTUs that were potentially relevant to discriminate the geographic origin of the fish being surveyed. Samples originating from farm A were the only ones exhibiting families Bacillaceae and Oxalobacteraceae (Fig. 6); at genus level, fish farm A was the only one were members of genus *Bacillus* (Bacillaceae) and *Cetobacterium* (Fusobacteriaceae) recorded (Fig. 7). While members of genus *Bacillus* are already routinely employed as probiotics in aquaculture^46,47^, the probiotic action of genus *Cetobacterium* is still being investigated^48^. On the other side, family Oxalobacteraceae is often associated with fish pathogens^49^. While the use of probiotics in aquaculture is a widely accepted practice, its implementation is still hampered by the lack of knowledge on the interaction that probiotics may have on the overall microbial community present in aquaculture systems^50^. In this context, taking into account the lower bacterial diversity recorded for fish farm A, the simultaneous presence of probiotic and pathogen bacterial taxa, it may be possible that the microbial community present in this environment may not functionally stable. Therefore, more or less dramatic shifts may be anticipated and likely affect the microbiome present in the fish mucus.

Concerning farms B and C, and although they shared a number of bacterial taxa, it was possible to discriminate both by the exclusive presence of genus *Desulfobacter* in farm B and genus *Colwellia* in farm C (Fig. 7). Previous studies reported *Desulfobacter* as an anaerobic sulphate-reducing bacteria associated with fish farm sediments, likely due to a high organic enrichment prompted by the accumulation of uneaten feed and faecal material^51,52^. Concerning genus *Colwellia*, it has been isolated from anaerobic tidal flat sediments^53,54^ and suggest that anaerobic processes may also be undergoing in fish farm C. These findings are likely to mirror the aquaculture practices employed by fish farmers. In other words, it is not uncommon to record anoxic areas in earth ponds employed for fish farming^55^, namely when aerators and automatic feeders are misplaced/misused and allow the accumulation of uneaten feed in the tank bottom^56^. These bacterial taxa acted as environmental markers of fish farms B and C, being promising markers when aiming to trace the geographic origin of farmed fish.

## Conclusions

The present study demonstrated that specific bacterial communities present in the skin mucus of European sea bass cultured in earth ponds yield unique signatures that allow to trace each fish to its respective geographic origin (fish farm of production). The combined use of PCR-DGGE and NGS are effective molecular tools that can make possible to pinpoint which bacterial taxa hold the potential to be used as natural and unique barcodes of farmed fish. These taxa (or their combination) have to be unique and not prone to dramatic temporal shifts during shelf life (which may blur or even erase such bacterial signatures). By gaining knowledge on which bacterial taxa are more likely to reveal the place of origin of the specimens being surveyed, screening can become faster and more cost-efficient. Future studies refining the approach employed in the present work may allow the implementation of more reliable traceability protocols along the first stages of supply chains trading fresh farmed fish.

## Electronic supplementary material

Supplementary Table S1 and S2
