# Supplementary material for: Bacterial communities 16S rDNA fingerprinting as a potential tracing tool for cultured seabass Dicentrarchus labrax
Source: Sci Rep. 2017 Sep 19;7:11862. doi: 10.1038/s41598-017-11552-y (PMC5605529; doi:10.1038/s41598-017-11552-y)
Supplement: Supplementary file 1 — Supplementary Table S1 and S2 [file 41598_2017_11552_MOESM1_ESM.pdf]

Potential use of bacterial communities fingerprint to trace the geographic origin of cultured seabass Dicentrarchus labrax

Tânia Pimentel[1*], Joana Marcelino[2], Fernando Ricardo[1], Amadeu M.V.M Soares[1] and Ricardo Calado[1*]

[1]Departamento de Biologia & CESAM, Universidade de Aveiro, Campus Universitário de Santiago, 3810-193 Aveiro, Portugal
[2]Instituto Universitário de Lisboa (ISCTE-IUL), DINÂMIA’CET, Avenida das Forças Armadas, 1649-026 Lisbon, Portugal

Corresponding authors:
* Tânia Pimentel (trpimentel@ua.pt)
* Ricardo Calado (rjcalado@hotmail.com)

Supplementary Table S1. Results of Kruskal-Wallis test and pairwise comparisons using Nemenyi test for bacterial communities (DGGE Band) among three semi-intensive fish farms: Farm A, Farm B and Farm C. Asterisks (*) highlight significant differences ($p \leq 0{,}05$).

	**Kruskal-Walllis test**		**Post Hoc Nemenyi test**		
			**Farm A *vs* Farm B**	**Farm A *vs* Farm C**	**Farm B *vs* Farm C**
**DGGE Band**	***Chi-Square* (df=2)**	***p*-value**	***p*-value**	***p*-value**	***p*-value**
1	8,112	**0,017***	**0,039***	**0,041***	0,994
2	0,943	0,624	-	-	-
3	2,693	0,260	-	-	-
4	4,286	0,117	-	-	-
5	7,020	**0,030***	0,800	0,136	**0,029***
6	0,781	0,677		-	-
7	2,000	0,368	-	-	-
8	2,756	0,252	-	-	-
9	5,749	0,056	-	-	-
10	2,660	0,265	-	-	-
11	9,882	**0,007***	1,000	**0,046***	0,086
12	7,578	**0,023***	**0,053***	0,890	0,193
13	13,291	**0,001***	1,000	**0,022***	**0,022***
14	11,022	**0,004***	**0,005***	0,495	0,117
15	2,000	0,368	-	-	-
16	11,594	**0,003***	**0,003***	0,371	0,117
17	2,602	0,272	-	-	-
18	9,882	**0,007***	1,000	0,086	**0,036***
19	12,465	**0,002***	0,157	**0,002***	0,235
20	10,261	**0,006***	0,874	**0,010***	**0,043***
21	9,380	**0,009***	**0,007***	0,157	0,452
22	3,140	0,208	-	-	-
23	1,086	0,581	-	-	-
24	0,787	0,675	-	-	-
25	8,207	**0,017***	**0,024***	0,933	0,061
26	3,963	0,138	-	-	-
27	1,570	0,455	-	-	-
28	4,022	0,134	-	-	-
29	9,125	**0,010***	**0,008***	0,136	0,539
30	5,139	0,077	-	-	-
31	9,448	**0,009***	**0,020***	**0,024***	0,997
32	8,732	**0,013***	**0,010***	0,270	0,370
33	1,089	0,580	-	-	-
34	5,139	0,077	-	-	-
35	0,020	0,990	-	-	-
36	7,317	**0,026***	**0,024***	0,452	0,333
37	5,040	0,080	-	-	-
38	5,829	0,056	-	-	-
39	1,689	0,430	-	-	-
40	3,648	0,161	-	-	-
41	9,551	**0,008***	**0,020***	**0,024***	0,997
42	3,339	0,188	-	-	-
43	1,219	0,544	-	-	-
44	2,022	0,364	-	-	-
45	1,563	0,458	-	-	-
46	4,286	0,117	-	-	-
47	7,280	**0,026***	**0,020***	0,270	0,490
48	4,286	0,117	-	-	-
49	5,780	0,056	-	-	-
50	2,000	0,368	-	-	-
51	1,079	0,583	-	-	-
52	0,943	0,624	-	-	-
53	7,356	**0,025***	0,997	0,073	**0,051***
54	8,301	**0,016***	**0,051***	0,920	0,146
55	1,049	0,592	-	-	-

56	9,727	**0,008***	**0,020***	**0,024***	0,997
57	4,615	0,100	-	-	-
58	8,112	**0,017***	0,051	**0,039***	0,994
59	2,602	0,272	-	-	-
60	3,722	0,156	-	-	-
61	2,000	0,368	-	-	-
62	2,600	0,272	-	-	-
63	7,006	**0,030***	0,411	**0,039***	0,473
64	2,299	0,317	-	-	-

Supplementary Table S2. Absolute abundance and taxonomic assignment of bacterial quality-filtered OTUs.

**OTU ID**	**Samples**			**Taxonomy**
	**Farm A**	**Farm B**	**Farm C**	**(Kingdom, Phylum, Class, Order, Family, Genus)**
1	3211	788	1340	Bacteria;Proteobacteria;Gammaproteobacteria;Pseudomonadales;Pseudomonadaceae;Pseudomonas;
2	237	10	168	Bacteria;Proteobacteria;Gammaproteobacteria;Enterobacteriales;Enterobacteriaceae;Serratia;
3	362	35	0	Bacteria;Proteobacteria;Gammaproteobacteria;Pseudomonadales;Moraxellaceae;Perlucidibaca;
4	0	70	27	Bacteria;Proteobacteria;Epsilonproteobacteria;Campylobacterales;Helicobacteraceae;Sulfurovum;
5	1	89	0	Bacteria;Bacteroidetes;unclassified;unclassified;unclassified;unclassified;
6	4	5	67	Bacteria;Proteobacteria;Alphaproteobacteria;Rhodobacterales;Rhodobacteraceae;unclassified;
7	44	11	21	Bacteria;Proteobacteria;Alphaproteobacteria;Rhodobacterales;Rhodobacteraceae;unclassified;
8	28	18	18	Bacteria;Proteobacteria;Alphaproteobacteria;Rhodobacterales;Rhodobacteraceae;unclassified;
9	25	24	12	Bacteria;Proteobacteria;Alphaproteobacteria;Rhodobacterales;Rhodobacteraceae;Loktanella;
10	40	4	13	Bacteria;Fusobacteria;Fusobacteria;Fusobacteriales;Fusobacteriaceae;unclassified;
11	0	56	0	Bacteria;Fusobacteria;Fusobacteria;Fusobacteriales;Fusobacteriaceae;unclassified;
12	3	33	11	Bacteria;Proteobacteria;Deltaproteobacteria;Desulfuromonadales;unclassified;unclassified;
13	1	36	6	Bacteria;Firmicutes;Clostridia;Clostridiales;Ruminococcaceae;unclassified;
14	0	39	3	Bacteria;Proteobacteria;Epsilonproteobacteria;Campylobacterales;Campylobacteraceae;Arcobacter;
15	1	29	11	Bacteria;Proteobacteria;Gammaproteobacteria;unclassified;unclassified;unclassified;
16	0	19	13	Bacteria;Firmicutes;Clostridia;Clostridiales;unclassified;unclassified;
17	3	18	9	Bacteria;Proteobacteria;Alphaproteobacteria;Rhodobacterales;Rhodobacteraceae;Sulfitobacter;
18	0	23	5	Bacteria;Proteobacteria;Epsilonproteobacteria;Campylobacterales;unclassified;unclassified;
19	1	25	1	Bacteria;unclassified;unclassified;unclassified;unclassified;unclassified;
20	0	17	9	Bacteria;Proteobacteria;Alphaproteobacteria;Rhodobacterales;Rhodobacteraceae;unclassified;
21	12	1	13	Bacteria;Bacteroidetes;Flavobacteria;Flavobacteriales;Flavobacteriaceae;unclassified;
22	0	8	18	Bacteria;Proteobacteria;Epsilonproteobacteria;Campylobacterales;unclassified;unclassified;
23	0	16	8	Bacteria;Proteobacteria;Alphaproteobacteria;Rhizobiales;unclassified;unclassified;
24	6	3	15	Bacteria;Proteobacteria;Gammaproteobacteria;Vibrionales;Vibrionaceae;Vibrio;
25	0	17	6	Bacteria;Proteobacteria;Alphaproteobacteria;Rhizobiales;Rhodobiaceae;unclassified;
26	0	21	2	Bacteria;Bacteroidetes;unclassified;unclassified;unclassified;unclassified;
27	0	23	0	Bacteria;Firmicutes;Clostridia;Clostridiales;unclassified;unclassified;
28	0	4	17	Bacteria;Proteobacteria;Alphaproteobacteria;Rhodobacterales;Rhodobacteraceae;unclassified;
29	0	14	7	Bacteria;Proteobacteria;Gammaproteobacteria;unclassified;unclassified;unclassified;
30	0	10	10	Bacteria;Proteobacteria;Gammaproteobacteria;unclassified;unclassified;unclassified;
31	0	20	0	Bacteria;Bacteroidetes;unclassified;unclassified;unclassified;unclassified;
32	0	5	14	Bacteria;Proteobacteria;Epsilonproteobacteria;Campylobacterales;Campylobacteraceae;Arcobacter;
33	0	19	0	Bacteria;Proteobacteria;Deltaproteobacteria;Desulfobacterales;Desulfobacteraceae;unclassified;
34	0	16	3	Bacteria;Bacteroidetes;unclassified;unclassified;unclassified;unclassified;
35	19	0	0	Bacteria;Firmicutes;Bacilli;Bacillales;Bacillaceae_1;Bacillus;
36	0	8	10	Bacteria;Proteobacteria;Alphaproteobacteria;Sphingomonadales;Erythrobacteraceae;unclassified;
37	16	1	1	Bacteria;Proteobacteria;Betaproteobacteria;unclassified;unclassified;unclassified;

38	1	9	8	Bacteria;Proteobacteria;Gammaproteobacteria;unclassified;unclassified;unclassified;
39	18	0	0	Bacteria;Proteobacteria;Gammaproteobacteria;Vibrionales;Vibrionaceae;Photobacterium;
40	9	5	3	Bacteria;Proteobacteria;Alphaproteobacteria;Rhodobacterales;Rhodobacteraceae;unclassified;
41	4	2	11	Bacteria;Proteobacteria;Gammaproteobacteria;unclassified;unclassified;unclassified;
42	5	5	6	Bacteria;Proteobacteria;Gammaproteobacteria;unclassified;unclassified;unclassified;
43	1	11	4	Bacteria;Actinobacteria;Actinobacteria;unclassified;unclassified;unclassified;
44	0	8	8	Bacteria;Proteobacteria;Epsilonproteobacteria;Campylobacterales;Helicobacteraceae;Sulfurimonas;
45	0	10	6	Bacteria;Proteobacteria;Gammaproteobacteria;unclassified;unclassified;unclassified;
46	3	12	0	Bacteria;Proteobacteria;Gammaproteobacteria;unclassified;unclassified;unclassified;
47	15	0	0	Bacteria;Fusobacteria;Fusobacteria;Fusobacteriales;Fusobacteriaceae;Cetobacterium;
48	1	13	0	Bacteria;Bacteroidetes;unclassified;unclassified;unclassified;unclassified;
49	0	0	14	Bacteria;Proteobacteria;unclassified;unclassified;unclassified;unclassified;
50	0	8	6	Bacteria;Firmicutes;Bacilli;Bacillales;Planococcaceae;Planococcus;
51	0	14	0	Bacteria;Bacteroidetes;unclassified;unclassified;unclassified;unclassified;
52	0	9	5	Bacteria;Proteobacteria;Gammaproteobacteria;unclassified;unclassified;unclassified;
53	3	6	4	Bacteria;Bacteroidetes;Flavobacteria;Flavobacteriales;Flavobacteriaceae;unclassified;
54	0	3	10	Bacteria;Proteobacteria;Alphaproteobacteria;Sphingomonadales;Erythrobacteraceae;unclassified;
55	0	0	13	Bacteria;Proteobacteria;Gammaproteobacteria;Alteromonadales;unclassified;unclassified;
56	0	6	7	Bacteria;Firmicutes;Clostridia;Clostridiales;Lachnospiraceae;unclassified;
57	0	6	6	Bacteria;Proteobacteria;Gammaproteobacteria;unclassified;unclassified;unclassified;
58	12	0	0	Bacteria;Proteobacteria;Gammaproteobacteria;Pseudomonadales;Pseudomonadaceae;Pseudomonas;
59	1	11	0	Bacteria;Bacteroidetes;unclassified;unclassified;unclassified;unclassified;
60	2	4	6	Bacteria;Proteobacteria;Alphaproteobacteria;Sneathiellales;Sneathiellaceae;Sneathiella;
61	7	5	0	Bacteria;Proteobacteria;unclassified;unclassified;unclassified;unclassified;
62	1	7	4	Bacteria;Proteobacteria;Deltaproteobacteria;Desulfobacterales;unclassified;unclassified;
63	1	10	0	Bacteria;Proteobacteria;Deltaproteobacteria;unclassified;unclassified;unclassified;
64	8	2	1	Bacteria;Actinobacteria;Actinobacteria;Actinomycetales;Microbacteriaceae;Agrococcus;
65	0	0	11	Bacteria;Proteobacteria;Gammaproteobacteria;Alteromonadales;Colwelliaceae;Colwellia;
66	0	10	1	Bacteria;Bacteroidetes;Sphingobacteria;Sphingobacteriales;Flammeovirgaceae;unclassified;
67	10	1	0	Bacteria;Proteobacteria;Betaproteobacteria;Burkholderiales;Comamonadaceae;Hydrogenophaga;
68	1	6	4	Bacteria;Actinobacteria;Actinobacteria;Acidimicrobiales;Acidimicrobiaceae;Ilumatobacter;
69	0	11	0	Bacteria;Firmicutes;unclassified;unclassified;unclassified;unclassified;
70	0	6	5	Bacteria;Bacteroidetes;unclassified;unclassified;unclassified;unclassified;
71	0	3	7	Bacteria;Proteobacteria;Alphaproteobacteria;Rhodobacterales;Rhodobacteraceae;unclassified;
72	0	8	2	Bacteria;Proteobacteria;Gammaproteobacteria;unclassified;unclassified;unclassified;
73	0	10	0	Bacteria;Proteobacteria;Epsilonproteobacteria;Campylobacterales;Campylobacteraceae;Arcobacter;
74	0	10	0	Bacteria;Firmicutes;Clostridia;Clostridiales;unclassified;unclassified;
75	0	2	8	Bacteria;Proteobacteria;Epsilonproteobacteria;Campylobacterales;Helicobacteraceae;Sulfurimonas;
76	0	0	10	Bacteria;Proteobacteria;Alphaproteobacteria;unclassified;unclassified;unclassified;
77	0	8	2	Bacteria;Firmicutes;Clostridia;Clostridiales;Lachnospiraceae;unclassified;
78	0	10	0	Bacteria;Bacteroidetes;unclassified;unclassified;unclassified;unclassified;
79	0	10	0	Bacteria;Proteobacteria;Epsilonproteobacteria;Campylobacterales;unclassified;unclassified;

80	0	7	2	Bacteria;Proteobacteria;Gammaproteobacteria;Chromatiales;Chromatiaceae;unclassified;
81	4	2	3	Bacteria;Proteobacteria;Alphaproteobacteria;Rhodobacterales;Rhodobacteraceae;Paracoccus;
82	0	0	9	Bacteria;Proteobacteria;Gammaproteobacteria;Alteromonadales;Colwelliaceae;Colwellia;
83	0	9	0	Bacteria;Proteobacteria;Alphaproteobacteria;unclassified;unclassified;unclassified;
84	0	1	8	Bacteria;Firmicutes;Clostridia;Clostridiales;Lachnospiraceae;unclassified;
85	9	0	0	Bacteria;Proteobacteria;Betaproteobacteria;Burkholderiales;Oxalobacteraceae;unclassified;
86	0	9	0	Bacteria;unclassified;unclassified;unclassified;unclassified;unclassified;
87	0	1	8	Bacteria;Proteobacteria;Alphaproteobacteria;Rhizobiales;unclassified;unclassified;
88	0	6	2	Bacteria;Proteobacteria;Gammaproteobacteria;Chromatiales;unclassified;unclassified;
89	0	8	0	Bacteria;Proteobacteria;Alphaproteobacteria;Rhodobacterales;Rhodobacteraceae;Jannaschia;
90	0	5	3	Bacteria;Proteobacteria;Gammaproteobacteria;unclassified;unclassified;unclassified;
91	0	4	4	Bacteria;Acidobacteria;Acidobacteria;Acidobacteriales;Acidobacteriaceae;Gp23
92	0	8	0	Bacteria;Proteobacteria;Deltaproteobacteria;Desulfobacterales;Desulfobacteraceae;unclassified;
93	8	0	0	Bacteria;Proteobacteria;Betaproteobacteria;Methylophilales;Methylophilaceae;unclassified;
94	0	8	0	Bacteria;Proteobacteria;Epsilonproteobacteria;Campylobacterales;unclassified;unclassified;
95	0	8	0	Bacteria;Bacteroidetes;unclassified;unclassified;unclassified;unclassified;
96	0	6	2	Bacteria;Proteobacteria;Alphaproteobacteria;Rhodobacterales;Rhodobacteraceae;unclassified;
97	7	0	1	Bacteria;Proteobacteria;Gammaproteobacteria;Alteromonadales;Alteromonadaceae;unclassified;
98	0	8	0	Bacteria;Bacteroidetes;unclassified;unclassified;unclassified;unclassified;
99	8	0	0	Bacteria;Proteobacteria;Gammaproteobacteria;Pseudomonadales;Pseudomonadaceae;Pseudomonas;
100	6	1	0	Bacteria;Proteobacteria;Betaproteobacteria;unclassified;unclassified;unclassified;
101	0	4	3	Bacteria;Proteobacteria;Gammaproteobacteria;unclassified;unclassified;unclassified;
102	0	1	6	Bacteria;Proteobacteria;Alphaproteobacteria;Sphingomonadales;Erythrobacteraceae;unclassified;
103	6	1	0	Bacteria;Proteobacteria;Alphaproteobacteria;Rhodobacterales;Rhodobacteraceae;unclassified;
104	0	4	3	Bacteria;unclassified;unclassified;unclassified;unclassified;unclassified;
105	0	4	3	Bacteria;Proteobacteria;Alphaproteobacteria;Rhodobacterales;Rhodobacteraceae;unclassified;
106	4	3	0	Bacteria;Proteobacteria;Gammaproteobacteria;Pseudomonadales;Moraxellaceae;Psychrobacter;
107	0	6	1	Bacteria;Bacteroidetes;unclassified;unclassified;unclassified;unclassified;
108	1	1	5	Bacteria;Proteobacteria;Alphaproteobacteria;Rhodobacterales;Rhodobacteraceae;unclassified;
109	0	0	7	Bacteria;unclassified;unclassified;unclassified;unclassified;unclassified;
110	0	7	0	Bacteria;Bacteroidetes;unclassified;unclassified;unclassified;unclassified;
111	0	0	7	Bacteria;Bacteroidetes;unclassified;unclassified;unclassified;unclassified;
112	0	7	0	Bacteria;Proteobacteria;Deltaproteobacteria;Desulfobacterales;Desulfobacteraceae;unclassified;
113	0	4	3	Bacteria;Proteobacteria;Gammaproteobacteria;unclassified;unclassified;unclassified;
114	0	6	0	Bacteria;Proteobacteria;Alphaproteobacteria;Rhizobiales;Phyllobacteriaceae;unclassified;
115	2	2	2	Bacteria;Proteobacteria;Alphaproteobacteria;Rhodospirillales;Rhodospirillaceae;unclassified;
116	1	4	1	Bacteria;Proteobacteria;unclassified;unclassified;unclassified;unclassified;
117	2	0	4	Bacteria;Proteobacteria;Gammaproteobacteria;unclassified;unclassified;unclassified;
118	0	6	0	Bacteria;Proteobacteria;Deltaproteobacteria;Desulfovibrionales;Desulfovibrionaceae;Desulfovibrio;
119	0	6	0	Bacteria;Firmicutes;Clostridia;Clostridiales;unclassified;unclassified;
120	0	5	1	Bacteria;Bacteroidetes;unclassified;unclassified;unclassified;unclassified;
121	2	4	0	Bacteria;Proteobacteria;Alphaproteobacteria;Rhodobacterales;Rhodobacteraceae;Sulfitobacter;

122	0	6	0	Bacteria;Bacteroidetes;unclassified;unclassified;unclassified;unclassified;
123	0	2	4	Bacteria;Proteobacteria;Deltaproteobacteria;Desulfovibrionales;Desulfovibrionaceae;Desulfovibrio;
124	0	1	5	Bacteria;Proteobacteria;Epsilonproteobacteria;Campylobacterales;Campylobacteraceae;unclassified;
125	0	5	1	Bacteria;Proteobacteria;Alphaproteobacteria;Rhodobacterales;Rhodobacteraceae;unclassified;
126	0	5	1	Bacteria;Proteobacteria;Deltaproteobacteria;Desulfobacterales;Desulfobacteraceae;unclassified;
127	6	0	0	Bacteria;Proteobacteria;Betaproteobacteria;Burkholderiales;Comamonadaceae;Polaromonas;
128	0	0	6	Bacteria;Proteobacteria;Deltaproteobacteria;Myxococcales;unclassified;unclassified;
129	0	1	5	Bacteria;Bacteroidetes;Flavobacteria;Flavobacteriales;Flavobacteriaceae;Polaribacter;
130	0	1	5	Bacteria;Proteobacteria;Alphaproteobacteria;Rhodobacterales;Rhodobacteraceae;unclassified;
131	0	3	3	Bacteria;Spirochaetes;Spirochaetes;Spirochaetales;Spirochaetaceae;Spirochaeta;
132	0	3	3	Bacteria;Proteobacteria;Epsilonproteobacteria;Campylobacterales;Campylobacteraceae;Arcobacter;
133	1	1	4	Bacteria;Proteobacteria;Alphaproteobacteria;Rhodobacterales;Rhodobacteraceae;unclassified;
134	0	5	0	Bacteria;Acidobacteria;Acidobacteria;Acidobacteriales;Acidobacteriaceae;Gp23
135	0	2	3	Bacteria;Proteobacteria;Gammaproteobacteria;unclassified;unclassified;unclassified;
136	1	2	2	Bacteria;Proteobacteria;Alphaproteobacteria;Rhodobacterales;Rhodobacteraceae;unclassified;
137	0	5	0	Bacteria;Proteobacteria;Alphaproteobacteria;Rhodobacterales;Rhodobacteraceae;unclassified;
138	1	3	1	Bacteria;Proteobacteria;Deltaproteobacteria;Desulfobacterales;Desulfobulbaceae;unclassified;
139	2	1	2	Bacteria;Proteobacteria;Alphaproteobacteria;Rhodobacterales;Rhodobacteraceae;unclassified;
140	0	4	1	Bacteria;Actinobacteria;Actinobacteria;unclassified;unclassified;unclassified;
141	0	5	0	Bacteria;Bacteroidetes;unclassified;unclassified;unclassified;unclassified;
142	0	3	2	Bacteria;unclassified;unclassified;unclassified;unclassified;unclassified;
143	1	2	2	Bacteria;Proteobacteria;Alphaproteobacteria;Sphingomonadales;Sphingomonadaceae;unclassified;
144	0	4	1	Bacteria;Proteobacteria;Alphaproteobacteria;unclassified;unclassified;unclassified;
145	0	4	1	Bacteria;Proteobacteria;Alphaproteobacteria;Rhizobiales;Rhodobiaceae;Anderseniella;
146	0	2	3	Bacteria;Proteobacteria;Alphaproteobacteria;Rhodobacterales;Rhodobacteraceae;unclassified;
147	0	0	5	Bacteria;Bacteroidetes;unclassified;unclassified;unclassified;unclassified;
148	0	5	0	Bacteria;unclassified;unclassified;unclassified;unclassified;unclassified;
149	0	4	1	Bacteria;unclassified;unclassified;unclassified;unclassified;unclassified;
150	5	0	0	Bacteria;unclassified;unclassified;unclassified;unclassified;unclassified;
151	0	5	0	Bacteria;Firmicutes;unclassified;unclassified;unclassified;unclassified;
152	0	2	3	Bacteria;Bacteroidetes;Flavobacteria;Flavobacteriales;Flavobacteriaceae;Krokinobacter;
153	0	3	2	Bacteria;Proteobacteria;Alphaproteobacteria;Rhodobacterales;Rhodobacteraceae;unclassified;
154	0	5	0	Bacteria;unclassified;unclassified;unclassified;unclassified;unclassified;
155	0	5	0	Bacteria;Bacteroidetes;unclassified;unclassified;unclassified;unclassified;
156	0	3	2	Bacteria;Bacteroidetes;Sphingobacteria;Sphingobacteriales;unclassified;unclassified;
157	0	3	2	Bacteria;Proteobacteria;Epsilonproteobacteria;Campylobacterales;Campylobacteraceae;Sulfurospirillum;
158	0	5	0	Bacteria;Bacteroidetes;unclassified;unclassified;unclassified;unclassified;
159	0	4	1	Bacteria;Bacteroidetes;unclassified;unclassified;unclassified;unclassified;
160	0	5	0	Bacteria;unclassified;unclassified;unclassified;unclassified;unclassified;
161	0	5	0	Bacteria;Proteobacteria;Epsilonproteobacteria;Campylobacterales;unclassified;unclassified;
162	0	0	5	Bacteria;Proteobacteria;Gammaproteobacteria;Alteromonadales;unclassified;unclassified;
163	0	5	0	Bacteria;Bacteroidetes;unclassified;unclassified;unclassified;unclassified;

164	0	4	1	Bacteria;Bacteroidetes;unclassified;unclassified;unclassified;unclassified;
165	0	0	4	Bacteria;Acidobacteria;Acidobacteria;Acidobacteriales;Acidobacteriaceae;Gp23
166	0	4	0	Bacteria;Proteobacteria;Deltaproteobacteria;Desulfobacterales;Desulfobacteraceae;unclassified;
167	0	4	0	Bacteria;Proteobacteria;Alphaproteobacteria;Rhodobacterales;Rhodobacteraceae;unclassified;
168	0	2	2	Bacteria;Proteobacteria;Alphaproteobacteria;Rhodobacterales;Rhodobacteraceae;Rhodovulum;
169	0	3	1	Bacteria;Acidobacteria;Acidobacteria;Acidobacteriales;Acidobacteriaceae;Gp10
170	1	2	1	Bacteria;Bacteroidetes;unclassified;unclassified;unclassified;unclassified;
171	0	1	3	Bacteria;Proteobacteria;Deltaproteobacteria;unclassified;unclassified;unclassified;
172	0	2	2	Bacteria;Firmicutes;Clostridia;Clostridiales;Lachnospiraceae;unclassified;
173	0	4	0	Bacteria;unclassified;unclassified;unclassified;unclassified;unclassified;
174	0	2	2	Bacteria;Proteobacteria;Alphaproteobacteria;unclassified;unclassified;unclassified;
175	0	0	4	Bacteria;Proteobacteria;Epsilonproteobacteria;Campylobacterales;unclassified;unclassified;
176	0	1	3	Bacteria;Proteobacteria;Gammaproteobacteria;Alteromonadales;Psychromonadaceae;Psychromonas;
177	3	1	0	Bacteria;Bacteroidetes;Flavobacteria;Flavobacteriales;Flavobacteriaceae;unclassified;
178	0	4	0	Bacteria;Proteobacteria;Deltaproteobacteria;unclassified;unclassified;unclassified;
179	0	4	0	Bacteria;Bacteroidetes;unclassified;unclassified;unclassified;unclassified;
180	1	0	3	Bacteria;Proteobacteria;Epsilonproteobacteria;Campylobacterales;Campylobacteraceae;Arcobacter;
181	0	4	0	Bacteria;Proteobacteria;Deltaproteobacteria;Desulfuromonadales;Desulfuromonadaceae;Desulfuromonas;
182	0	4	0	Bacteria;Bacteroidetes;unclassified;unclassified;unclassified;unclassified;
183	0	1	3	Bacteria;Proteobacteria;Alphaproteobacteria;Rhodobacterales;Rhodobacteraceae;Octadecabacter;
184	0	4	0	Bacteria;Bacteroidetes;unclassified;unclassified;unclassified;unclassified;
185	0	0	4	Bacteria;Proteobacteria;Alphaproteobacteria;unclassified;unclassified;unclassified;
186	0	4	0	Bacteria;Bacteroidetes;unclassified;unclassified;unclassified;unclassified;
187	0	0	4	Bacteria;Proteobacteria;Gammaproteobacteria;Vibrionales;Vibrionaceae;unclassified;
188	0	3	1	Bacteria;Proteobacteria;Gammaproteobacteria;Chromatiales;unclassified;unclassified;
189	0	4	0	Bacteria;Bacteroidetes;unclassified;unclassified;unclassified;unclassified;
190	0	4	0	Bacteria;Proteobacteria;Gammaproteobacteria;unclassified;unclassified;unclassified;
191	2	2	0	Bacteria;Bacteroidetes;unclassified;unclassified;unclassified;unclassified;
192	4	0	0	Bacteria;Proteobacteria;Gammaproteobacteria;Vibrionales;Vibrionaceae;Photobacterium;
193	0	3	1	Bacteria;Bacteroidetes;Sphingobacteria;Sphingobacteriales;unclassified;unclassified;
194	0	4	0	Bacteria;Firmicutes;Clostridia;Clostridiales;unclassified;unclassified;
195	2	2	0	Bacteria;Proteobacteria;Deltaproteobacteria;Desulfuromonadales;Desulfuromonadaceae;unclassified;
196	0	4	0	Bacteria;Bacteroidetes;unclassified;unclassified;unclassified;unclassified;
197	1	1	2	Bacteria;Proteobacteria;Alphaproteobacteria;Sphingomonadales;Erythrobacteraceae;unclassified;
198	1	3	0	Bacteria;Bacteroidetes;unclassified;unclassified;unclassified;unclassified;
199	0	4	0	Bacteria;Proteobacteria;Deltaproteobacteria;Bdellovibrionales;Bacteriovoracaceae;unclassified;
200	4	0	0	Bacteria;Bacteroidetes;Flavobacteria;Flavobacteriales;Flavobacteriaceae;Chryseobacterium;
201	0	4	0	Bacteria;Proteobacteria;Epsilonproteobacteria;Campylobacterales;Campylobacteraceae;unclassified;
202	1	3	0	Bacteria;Firmicutes;Bacilli;Bacillales;Bacillales_Incertae_Sedis_XII;Exiguobacterium;
203	0	4	0	Bacteria;unclassified;unclassified;unclassified;unclassified;unclassified;
204	1	0	3	Bacteria;Proteobacteria;Alphaproteobacteria;Caulobacterales;Hyphomonadaceae;Litorimonas;
205	0	4	0	Bacteria;Proteobacteria;Alphaproteobacteria;Rhodobacterales;Rhodobacteraceae;unclassified;

206	1	2	1	Bacteria;Proteobacteria;Betaproteobacteria;unclassified;unclassified;unclassified;
207	0	2	2	Bacteria;Proteobacteria;Gammaproteobacteria;unclassified;unclassified;unclassified;
208	4	0	0	Bacteria;Proteobacteria;Betaproteobacteria;Burkholderiales;Comamonadaceae;unclassified;
209	0	0	4	Bacteria;Firmicutes;Bacilli;Bacillales;Planococcaceae;Planomicrobium;
210	3	0	1	Bacteria;Bacteroidetes;Bacteroidia;Bacteroidales;unclassified;unclassified;
211	0	1	3	Bacteria;unclassified;unclassified;unclassified;unclassified;unclassified;
212	0	2	2	Bacteria;Proteobacteria;Alphaproteobacteria;Rhodobacterales;Rhodobacteraceae;unclassified;
213	0	3	1	Bacteria;Proteobacteria;Gammaproteobacteria;Alteromonadales;Alteromonadaceae;Alteromonas;
214	0	3	0	Bacteria;Proteobacteria;Deltaproteobacteria;Myxococcales;unclassified;unclassified;
215	0	3	0	Bacteria;Proteobacteria;Alphaproteobacteria;Rhodobacterales;Rhodobacteraceae;Sulfitobacter;
216	0	3	0	Bacteria;unclassified;unclassified;unclassified;unclassified;unclassified;
217	0	3	0	Bacteria;Proteobacteria;Gammaproteobacteria;Chromatiales;Chromatiaceae;unclassified;
218	0	2	1	Bacteria;Proteobacteria;Alphaproteobacteria;Rhizobiales;Hyphomicrobiaceae;unclassified;
219	0	1	2	Bacteria;Proteobacteria;Deltaproteobacteria;unclassified;unclassified;unclassified;
220	0	0	3	Bacteria;Firmicutes;Bacilli;Lactobacillales;Streptococcaceae;Streptococcus;
221	0	0	3	Bacteria;Proteobacteria;Gammaproteobacteria;Vibrionales;Vibrionaceae;unclassified;
222	0	0	3	Bacteria;Bacteroidetes;unclassified;unclassified;unclassified;unclassified;
223	0	3	0	Bacteria;Proteobacteria;Deltaproteobacteria;Desulfobacterales;Desulfobacteraceae;Desulfobacter;
224	1	0	2	Bacteria;Proteobacteria;Alphaproteobacteria;Rhodobacterales;Rhodobacteraceae;Ruegeria;
225	0	3	0	Bacteria;Proteobacteria;Alphaproteobacteria;Rhodobacterales;Rhodobacteraceae;unclassified;
226	1	2	0	Bacteria;Bacteroidetes;unclassified;unclassified;unclassified;unclassified;
227	3	0	0	Bacteria;Proteobacteria;Alphaproteobacteria;Sphingomonadales;Sphingomonadaceae;Sphingomonas;
228	0	2	1	Bacteria;Bacteroidetes;Flavobacteria;Flavobacteriales;Flavobacteriaceae;unclassified;
229	0	0	3	Bacteria;Proteobacteria;Gammaproteobacteria;unclassified;unclassified;unclassified;
230	3	0	0	Bacteria;Bacteroidetes;unclassified;unclassified;unclassified;unclassified;
231	0	3	0	Bacteria;Proteobacteria;Deltaproteobacteria;Desulfobacterales;Desulfobacteraceae;Desulfobacula;
232	0	3	0	Bacteria;Bacteroidetes;unclassified;unclassified;unclassified;unclassified;
233	3	0	0	Bacteria;Proteobacteria;Gammaproteobacteria;unclassified;unclassified;unclassified;
234	0	0	3	Bacteria;Proteobacteria;Deltaproteobacteria;Desulfobacterales;Desulfobacteraceae;unclassified;
235	0	1	2	Bacteria;Proteobacteria;Gammaproteobacteria;unclassified;unclassified;unclassified;
236	0	3	0	Bacteria;Proteobacteria;Deltaproteobacteria;Desulfobacterales;Desulfobacteraceae;unclassified;
237	0	2	1	Bacteria;Firmicutes;Clostridia;Clostridiales;unclassified;unclassified;
238	3	0	0	Bacteria;Proteobacteria;Gammaproteobacteria;unclassified;unclassified;unclassified;
239	0	2	1	Bacteria;Proteobacteria;Gammaproteobacteria;unclassified;unclassified;unclassified;
240	0	1	2	Bacteria;Proteobacteria;Gammaproteobacteria;unclassified;unclassified;unclassified;
241	0	0	3	Bacteria;Proteobacteria;Gammaproteobacteria;unclassified;unclassified;unclassified;
242	0	2	1	Bacteria;Bacteroidetes;unclassified;unclassified;unclassified;unclassified;
243	2	1	0	Bacteria;Bacteroidetes;Flavobacteria;Flavobacteriales;Cryomorphaceae;unclassified;
244	0	3	0	Bacteria;Bacteroidetes;Flavobacteria;Flavobacteriales;Flavobacteriaceae;unclassified;
245	0	0	3	Bacteria;Proteobacteria;Deltaproteobacteria;Desulfobacterales;Desulfobulbaceae;unclassified;
246	0	3	0	Bacteria;Proteobacteria;Betaproteobacteria;unclassified;unclassified;unclassified;
247	0	0	3	Bacteria;Firmicutes;Clostridia;unclassified;unclassified;unclassified;

248	0	3	0	Bacteria;Proteobacteria;Deltaproteobacteria;Myxococcales;unclassified;unclassified;
249	0	3	0	Bacteria;Proteobacteria;Deltaproteobacteria;Desulfobacterales;Desulfobacteraceae;unclassified;
250	0	1	2	Bacteria;Bacteroidetes;Sphingobacteria;Sphingobacteriales;unclassified;unclassified;
251	0	0	3	Bacteria;Proteobacteria;Gammaproteobacteria;Pseudomonadales;Moraxellaceae;Psychrobacter;
252	0	3	0	Bacteria;Bacteroidetes;unclassified;unclassified;unclassified;unclassified;
253	0	3	0	Bacteria;Bacteroidetes;unclassified;unclassified;unclassified;unclassified;
254	0	3	0	Bacteria;Bacteroidetes;unclassified;unclassified;unclassified;unclassified;
255	0	3	0	Bacteria;Bacteroidetes;Flavobacteria;Flavobacteriales;unclassified;unclassified;
256	0	0	3	Bacteria;Bacteroidetes;unclassified;unclassified;unclassified;unclassified;
257	0	0	3	Bacteria;Proteobacteria;Alphaproteobacteria;Rhodobacterales;Rhodobacteraceae;unclassified;
258	0	3	0	Bacteria;Proteobacteria;Gammaproteobacteria;Chromatiales;Chromatiaceae;unclassified;
259	0	3	0	Bacteria;Proteobacteria;Deltaproteobacteria;Desulfuromonadales;unclassified;unclassified;
260	0	0	3	Bacteria;Proteobacteria;Gammaproteobacteria;Alteromonadales;Colwelliaceae;Thalassomonas;
261	0	2	1	Bacteria;Bacteroidetes;Sphingobacteria;Sphingobacteriales;unclassified;unclassified;
262	0	3	0	Bacteria;Firmicutes;Clostridia;Clostridiales;Lachnospiraceae;unclassified;
263	0	0	3	Bacteria;Proteobacteria;Gammaproteobacteria;unclassified;unclassified;unclassified;
264	0	0	3	Bacteria;unclassified;unclassified;unclassified;unclassified;unclassified;
265	0	3	0	Bacteria;Firmicutes;Clostridia;Clostridiales;Ruminococcaceae;unclassified;
266	0	3	0	Bacteria;unclassified;unclassified;unclassified;unclassified;unclassified;
267	0	3	0	Bacteria;Proteobacteria;Deltaproteobacteria;Desulfobacterales;Desulfobacteraceae;Desulfobacter;
268	0	3	0	Bacteria;unclassified;unclassified;unclassified;unclassified;unclassified;
269	0	2	1	Bacteria;Proteobacteria;Epsilonproteobacteria;Campylobacterales;Campylobacteraceae;Arcobacter;
270	0	1	2	Bacteria;Proteobacteria;Alphaproteobacteria;unclassified;unclassified;unclassified;
271	0	0	3	Bacteria;Firmicutes;Clostridia;Clostridiales;unclassified;unclassified;
272	0	3	0	Bacteria;Bacteroidetes;unclassified;unclassified;unclassified;unclassified;
273	3	0	0	Bacteria;Proteobacteria;Gammaproteobacteria;Pseudomonadales;Moraxellaceae;Psychrobacter;
274	0	3	0	Bacteria;Proteobacteria;Deltaproteobacteria;Desulfuromonadales;Desulfuromonadaceae;Desulfuromonas;
275	0	3	0	Bacteria;unclassified;unclassified;unclassified;unclassified;unclassified;
276	0	1	2	Bacteria;Firmicutes;Clostridia;Clostridiales;Lachnospiraceae;unclassified;
277	0	3	0	Bacteria;unclassified;unclassified;unclassified;unclassified;unclassified;
278	0	0	3	Bacteria;Firmicutes;Bacilli;Bacillales;Paenibacillaceae_1;Paenibacillus;
279	2	0	1	Bacteria;Proteobacteria;Gammaproteobacteria;Alteromonadales;Alteromonadaceae;Agarivorans;
280	0	1	2	Bacteria;Bacteroidetes;unclassified:unclassified;unclassified;unclassified;
281	0	3	0	Bacteria;Proteobacteria;Gammaproteobacteria;unclassified;unclassified;unclassified;
282	0	2	1	Bacteria;Actinobacteria;Actinobacteria;Acidimicrobiales;Acidimicrobiaceae;Ilumatobacter;
283	0	0	3	Bacteria;Proteobacteria;Gammaproteobacteria;unclassified;unclassified;unclassified;
284	0	2	1	Bacteria;Proteobacteria;Gammaproteobacteria;unclassified;unclassified;unclassified;
285	0	1	2	Bacteria;Bacteroidetes;Sphingobacteria;Sphingobacteriales;unclassified;unclassified;
286	0	2	1	Bacteria;Proteobacteria;Gammaproteobacteria;unclassified;unclassified;unclassified;
287	0	2	1	Bacteria;Proteobacteria;Deltaproteobacteria;Desulfuromonadales;unclassified;unclassified;
288	2	0	1	Bacteria;Bacteroidetes;unclassified;unclassified;unclassified;unclassified;
289	0	1	2	Bacteria;Proteobacteria;Alphaproteobacteria;unclassified;unclassified;unclassified;

290	0	2	0	Bacteria;Proteobacteria;Alphaproteobacteria;Rhodobacterales;Rhodobacteraceae;unclassified;
291	0	0	2	Bacteria;Proteobacteria;Gammaproteobacteria;Vibrionales;Vibrionaceae;Photobacterium;
292	0	1	1	Bacteria;Proteobacteria;Alphaproteobacteria;Rhodobacterales;Rhodobacteraceae;unclassified;
293	0	0	2	Bacteria;Proteobacteria;Gammaproteobacteria;Vibrionales;Vibrionaceae;Aliivibrio;
294	0	1	1	Bacteria;Actinobacteria;Actinobacteria;Acidimicrobiales;Acidimicrobiaceae;Ilumatobacter;
295	0	1	1	Bacteria;Proteobacteria;Gammaproteobacteria;unclassified;unclassified;unclassified;
296	2	0	0	Bacteria;Actinobacteria;Actinobacteria;Actinomycetales;Micrococcaceae;Kocuria;
297	2	0	0	Bacteria;Proteobacteria;Alphaproteobacteria;unclassified;unclassified;unclassified;
298	0	1	1	Bacteria;Bacteroidetes;unclassified;unclassified;unclassified;unclassified;
299	0	1	1	Bacteria;Proteobacteria;Gammaproteobacteria;unclassified;unclassified;unclassified;
300	0	2	0	Bacteria;Proteobacteria;Alphaproteobacteria;Rhodobacterales;Rhodobacteraceae;unclassified;
301	0	0	2	Bacteria;unclassified;unclassified;unclassified;unclassified;unclassified;
302	0	2	0	Bacteria;Bacteroidetes;unclassified;unclassified;unclassified;unclassified;
303	0	1	1	Bacteria;Proteobacteria;Gammaproteobacteria;Chromatiales;unclassified;unclassified;
304	0	0	2	Bacteria;Acidobacteria;Acidobacteria;Acidobacteriales;Acidobacteriaceae;Gp10
305	0	2	0	Bacteria;Proteobacteria;Alphaproteobacteria;Rhodobacterales;Rhodobacteraceae;unclassified;
306	0	0	2	Bacteria;Proteobacteria;Alphaproteobacteria;Rhodobacterales;Rhodobacteraceae;unclassified;
307	0	0	2	Bacteria;Proteobacteria;Gammaproteobacteria;unclassified;unclassified;unclassified;
308	0	0	2	Bacteria;Proteobacteria;Deltaproteobacteria;Desulfobacterales;Desulfobacteraceae;unclassified;
309	0	2	0	Bacteria;unclassified;unclassified;unclassified;unclassified;unclassified;
310	0	2	0	Bacteria;Proteobacteria;Gammaproteobacteria;unclassified;unclassified;unclassified;
311	0	0	2	Bacteria;Proteobacteria;Deltaproteobacteria;Desulfobacterales;Desulfobacteraceae;unclassified;
312	0	1	1	Bacteria;Proteobacteria;Alphaproteobacteria;Rhodobacterales;Rhodobacteraceae;unclassified;
313	0	2	0	Bacteria;Proteobacteria;unclassified;unclassified;unclassified;unclassified;
314	0	1	1	Bacteria;Bacteroidetes;unclassified;unclassified;unclassified;unclassified;
315	0	1	1	Bacteria;Bacteroidetes;Flavobacteria;Flavobacteriales;Flavobacteriaceae;Maribacter;
316	0	0	2	Bacteria;Proteobacteria;Alphaproteobacteria;unclassified;unclassified;unclassified;
317	0	2	0	Bacteria;unclassified;unclassified;unclassified;unclassified;unclassified;
318	0	2	0	Bacteria;Proteobacteria;unclassified;unclassified;unclassified;unclassified;
319	0	2	0	Bacteria;Proteobacteria;unclassified;unclassified;unclassified;unclassified;
320	0	0	2	Bacteria;Bacteroidetes;unclassified;unclassified;unclassified;unclassified;
321	0	0	2	Bacteria;unclassified;unclassified;unclassified;unclassified;unclassified;
322	0	2	0	Bacteria;Proteobacteria;Deltaproteobacteria;Desulfobacterales;Desulfobulbaceae;unclassified;
323	0	0	2	Bacteria;Proteobacteria;Deltaproteobacteria;Desulfuromonadales;Desulfuromonadaceae;Desulfuromonas;
324	0	2	0	Bacteria;Proteobacteria;Alphaproteobacteria;Sphingomonadales;unclassified;unclassified;
325	1	1	0	Bacteria;Bacteroidetes;Sphingobacteria;Sphingobacteriales;unclassified;unclassified;
326	0	1	1	Bacteria;Proteobacteria;Deltaproteobacteria;Desulfobacterales;Desulfobacteraceae;Desulfosarcina;
327	0	2	0	Bacteria;Proteobacteria;Gammaproteobacteria;unclassified;unclassified;unclassified;
328	0	1	1	Bacteria;Bacteroidetes;Sphingobacteria;Sphingobacteriales;unclassified;unclassified;
329	0	1	1	Bacteria;Proteobacteria;Gammaproteobacteria;unclassified;unclassified;unclassified;
330	0	0	2	Bacteria;unclassified;unclassified;unclassified;unclassified;unclassified;
331	0	2	0	Bacteria;Proteobacteria;Gammaproteobacteria;Oceanospirillales;Oceanospirillaceae;Oleispira;

332	0	0	2	Bacteria;Proteobacteria;Gammaproteobacteria;unclassified;unclassified;unclassified;
333	0	1	1	Bacteria;Acidobacteria;Acidobacteria;Acidobacteriales;Acidobacteriaceae;Gp26
334	0	2	0	Bacteria;Proteobacteria;Deltaproteobacteria;Desulfobacterales;Desulfobacteraceae;unclassified;
335	0	2	0	Bacteria;Bacteroidetes;unclassified;unclassified;unclassified;unclassified;
336	0	1	1	Bacteria;unclassified;unclassified;unclassified;unclassified;unclassified;
337	0	2	0	Bacteria;Proteobacteria;Gammaproteobacteria;unclassified;unclassified;unclassified;
338	0	2	0	Bacteria;Proteobacteria;Deltaproteobacteria;Desulfobacterales;Desulfobacteraceae;Desulfobacter;
339	0	0	2	Bacteria;Acidobacteria;unclassified;unclassified;unclassified;unclassified;
340	0	1	1	Bacteria;Bacteroidetes;Flavobacteria;Flavobacteriales;Flavobacteriaceae;unclassified;
341	0	0	2	Bacteria;Firmicutes;unclassified;unclassified;unclassified;unclassified;
342	0	0	2	Bacteria;Proteobacteria;Alphaproteobacteria;Rhizobiales;unclassified;unclassified;
343	0	2	0	Bacteria;Proteobacteria;unclassified;unclassified;unclassified;unclassified;
344	0	0	2	Bacteria;Proteobacteria;Gammaproteobacteria;unclassified;unclassified;unclassified;
345	0	2	0	Bacteria;Proteobacteria;Alphaproteobacteria;unclassified;unclassified;unclassified;
346	0	1	1	Bacteria;unclassified;unclassified;unclassified;unclassified;unclassified;
347	0	2	0	Bacteria;Proteobacteria;Gammaproteobacteria;cellvibrionales;Halieaceae;Congregibacter;
348	0	1	1	Bacteria;Bacteroidetes;Sphingobacteria;Sphingobacteriales;unclassified;unclassified;
349	0	1	1	Bacteria;Bacteroidetes;unclassified;unclassified;unclassified;unclassified;
350	0	2	0	Bacteria;Bacteroidetes;Flavobacteria;Flavobacteriales;Flavobacteriaceae;Salegentibacter;
351	0	2	0	Bacteria;Bacteroidetes;unclassified;unclassified;unclassified;unclassified;
352	0	2	0	Bacteria;Firmicutes;Clostridia;Clostridiales;unclassified;unclassified;
353	0	2	0	Bacteria;Proteobacteria;Alphaproteobacteria;unclassified;unclassified;unclassified;
354	0	2	0	Bacteria;Bacteroidetes;unclassified;unclassified;unclassified;unclassified;
355	0	2	0	Bacteria;Bacteroidetes;Flavobacteria;Flavobacteriales;Flavobacteriaceae;unclassified;
356	0	0	2	Bacteria;Bacteroidetes;unclassified;unclassified;unclassified;unclassified;
357	0	1	1	Bacteria;Bacteroidetes;Sphingobacteria;Sphingobacteriales;Flammeovirgaceae;unclassified;
358	0	2	0	Bacteria;Proteobacteria;Gammaproteobacteria;Chromatiales;Chromatiaceae;unclassified;
359	0	2	0	Bacteria;Proteobacteria;Epsilonproteobacteria;Campylobacterales;Campylobacteraceae;Sulfurospirillum;
360	0	1	1	Bacteria;Proteobacteria;Deltaproteobacteria;Desulfobacterales;Desulfobulbaceae;unclassified;
361	0	2	0	Bacteria;Bacteroidetes;unclassified;unclassified;unclassified;unclassified;
362	0	0	2	Bacteria;Bacteroidetes;unclassified;unclassified;unclassified;unclassified;
363	0	0	2	Bacteria;unclassified;unclassified;unclassified;unclassified;unclassified;
364	0	1	1	Bacteria;unclassified;unclassified;unclassified;unclassified;unclassified;
365	0	0	2	Bacteria;Proteobacteria;Alphaproteobacteria;Rhizobiales;unclassified;unclassified;
366	0	2	0	Bacteria;Proteobacteria;Deltaproteobacteria;Desulfuromonadales;Desulfuromonadaceae;unclassified;
367	0	0	2	Bacteria;Proteobacteria;Gammaproteobacteria;unclassified;unclassified;unclassified;
368	0	2	0	Bacteria;Proteobacteria;Gammaproteobacteria;unclassified;unclassified;unclassified;
369	0	0	2	Bacteria;Bacteroidetes;unclassified;unclassified;unclassified;unclassified;
370	0	2	0	Bacteria;unclassified;unclassified;unclassified;unclassified;unclassified;
371	0	2	0	Bacteria;Proteobacteria;Alphaproteobacteria;Rhodobacterales;Rhodobacteraceae;unclassified;
372	0	2	0	Bacteria;Bacteroidetes;Bacteroidia;Bacteroidales;unclassified;unclassified;
373	0	2	0	Bacteria;Acidobacteria;Acidobacteria;Acidobacteriales;Acidobacteriaceae;Gp23

374	0	2	0	Bacteria;Bacteroidetes;unclassified;unclassified;unclassified;unclassified;
375	0	2	0	Bacteria;Bacteroidetes;unclassified;unclassified;unclassified;unclassified;
376	0	2	0	Bacteria;Bacteroidetes;unclassified;unclassified;unclassified;unclassified;
377	0	2	0	Bacteria;Proteobacteria;Gammaproteobacteria;unclassified;unclassified;unclassified;
378	0	2	0	Bacteria;Firmicutes;unclassified;unclassified;unclassified;unclassified;
379	0	2	0	Bacteria;unclassified;unclassified;unclassified;unclassified;unclassified;
380	0	2	0	Bacteria;Proteobacteria;Deltaproteobacteria;Myxococcales;unclassified;unclassified;
381	0	0	2	Bacteria;Actinobacteria;Actinobacteria;Actinomycetales;unclassified;unclassified;
382	0	2	0	Bacteria;Bacteroidetes;unclassified;unclassified;unclassified;unclassified;
383	0	1	1	Bacteria;Actinobacteria;Actinobacteria;unclassified;unclassified;unclassified;
384	0	1	1	Bacteria;Proteobacteria;Alphaproteobacteria;Rhodobacterales;Rhodobacteraceae;unclassified;
385	0	2	0	Bacteria;Proteobacteria;Deltaproteobacteria;Desulfobacterales;Desulfobacteraceae;unclassified;
386	0	1	1	Bacteria;Bacteroidetes;unclassified;unclassified;unclassified;unclassified;
387	0	2	0	Bacteria;Proteobacteria;Deltaproteobacteria;Desulfobacterales;Desulfobulbaceae;unclassified;
388	0	2	0	Bacteria;Firmicutes;Clostridia;Clostridiales;Lachnospiraceae;unclassified;
389	0	0	2	Bacteria;Actinobacteria;Actinobacteria;unclassified;unclassified;unclassified;
390	0	0	2	Bacteria;Bacteroidetes;Sphingobacteria;Sphingobacteriales;unclassified;unclassified;
391	1	1	0	Bacteria;Proteobacteria;Gammaproteobacteria;unclassified;unclassified;unclassified;
392	0	0	2	Bacteria;Proteobacteria;Epsilonproteobacteria;Campylobacterales;unclassified;unclassified;
393	2	0	0	Bacteria;Proteobacteria;Gammaproteobacteria;Pseudomonadales;Pseudomonadaceae;unclassified;
394	2	0	0	Bacteria;Proteobacteria;Gammaproteobacteria;Pseudomonadales;Pseudomonadaceae;Pseudomonas;
395	0	1	1	Bacteria;Proteobacteria;Gammaproteobacteria;unclassified;unclassified;unclassified;
396	0	0	2	Bacteria;Proteobacteria;Alphaproteobacteria;unclassified;unclassified;unclassified;
397	0	1	1	Bacteria;Proteobacteria;Alphaproteobacteria;Rhodobacterales;Rhodobacteraceae;unclassified;
398	0	2	0	Bacteria;Bacteroidetes;unclassified;unclassified;unclassified;unclassified;
399	0	2	0	Bacteria;Proteobacteria;Alphaproteobacteria;Sneathiellales;Sneathiellaceae;Sneathiella;
400	0	2	0	Bacteria;Bacteroidetes;unclassified;unclassified;unclassified;unclassified;
401	0	0	2	Bacteria;Proteobacteria;Alphaproteobacteria;Rhodobacterales;Rhodobacteraceae;unclassified;
402	0	2	0	Bacteria;Bacteroidetes;Flavobacteria;Flavobacteriales;unclassified;unclassified;
403	0	2	0	Bacteria;Bacteroidetes;unclassified;unclassified;unclassified;unclassified;
404	2	0	0	Bacteria;Proteobacteria;Gammaproteobacteria;Alteromonadales;Shewanellaceae;Shewanella;
405	0	0	2	Bacteria;Bacteroidetes;unclassified;unclassified;unclassified;unclassified;
406	0	2	0	Bacteria;Bacteroidetes;unclassified;unclassified;unclassified;unclassified;
407	0	2	0	Bacteria;Proteobacteria;Gammaproteobacteria;unclassified;unclassified;unclassified;
408	0	2	0	Bacteria;Firmicutes;Clostridia;Clostridiales;unclassified;unclassified;
409	0	0	2	Bacteria;Proteobacteria;unclassified;unclassified;unclassified;unclassified;
410	2	0	0	Bacteria;Firmicutes;Bacilli;Bacillales;Bacillaceae_1;Bacillus;
411	0	2	0	Bacteria;unclassified;unclassified;unclassified;unclassified;unclassified;
412	2	0	0	Bacteria;Proteobacteria;Gammaproteobacteria;unclassified;unclassified;unclassified;
413	0	1	1	Bacteria;Proteobacteria;Gammaproteobacteria;Chromatiales;Granulosicoccaceae;Granulosicoccus;
414	0	0	2	Bacteria;Proteobacteria;Gammaproteobacteria;unclassified;unclassified;unclassified;
415	0	1	1	Bacteria;Proteobacteria;Deltaproteobacteria;Desulfobacterales;Desulfobacteraceae;unclassified;

416	0	2	0	Bacteria;Bacteroidetes;Sphingobacteria;Sphingobacteriales;Flammeovirgaceae;Fulvivirga;
417	0	0	2	Bacteria;Proteobacteria;Epsilonproteobacteria;Campylobacterales;Helicobacteraceae;Sulfurovum;
418	0	0	2	Bacteria;Proteobacteria;Epsilonproteobacteria;Campylobacterales;Campylobacteraceae;Arcobacter;
419	0	2	0	Bacteria;Proteobacteria;Deltaproteobacteria;Desulfobacterales;Desulfobacteraceae;unclassified;
420	0	2	0	Bacteria;Proteobacteria;Gammaproteobacteria;unclassified;unclassified;unclassified;
421	0	1	1	Bacteria;Proteobacteria;Gammaproteobacteria;unclassified;unclassified;unclassified;
422	0	2	0	Bacteria;unclassified;unclassified;unclassified;unclassified;unclassified;
423	0	2	0	Bacteria;unclassified;unclassified;unclassified;unclassified;unclassified;
424	0	2	0	Bacteria;unclassified;unclassified;unclassified;unclassified;unclassified;
425	2	0	0	Bacteria;Proteobacteria;Deltaproteobacteria;Desulfuromonadales;Desulfuromonadaceae;Desulfuromonas;
426	0	0	2	Bacteria;Proteobacteria;Gammaproteobacteria;unclassified;unclassified;unclassified;
427	2	0	0	Bacteria;Firmicutes;Bacilli;Bacillales;Bacillaceae_1;Bacillus;
428	1	1	0	Bacteria;Bacteroidetes;Flavobacteria;Flavobacteriales;unclassified;unclassified;
429	2	0	0	Bacteria;Proteobacteria;Gammaproteobacteria;Xanthomonadales;Xanthomonadaceae;unclassified;
430	0	2	0	Bacteria;Bacteroidetes;unclassified;unclassified;unclassified;unclassified;
431	0	2	0	Bacteria;Bacteroidetes;unclassified;unclassified;unclassified;unclassified;
432	0	1	1	Bacteria;Bacteroidetes;Sphingobacteria;Sphingobacteriales;Flammeovirgaceae;unclassified;
433	0	2	0	Bacteria;Bacteroidetes;unclassified;unclassified;unclassified;unclassified;
434	0	0	2	Bacteria;Proteobacteria;Alphaproteobacteria;Rhodobacterales;Rhodobacteraceae;unclassified;
435	0	0	2	Bacteria;Proteobacteria;Gammaproteobacteria;unclassified;unclassified;unclassified;
436	0	2	0	Bacteria;Proteobacteria;Deltaproteobacteria;Desulfobacterales;unclassified;unclassified;
437	0	1	1	Bacteria;unclassified;unclassified;unclassified;unclassified;unclassified;
438	0	0	2	Bacteria;Bacteroidetes;unclassified;unclassified;unclassified;unclassified;
439	0	0	2	Bacteria;Proteobacteria;Gammaproteobacteria;unclassified;unclassified;unclassified;
440	0	1	1	Bacteria;unclassified;unclassified;unclassified;unclassified;unclassified;
441	0	1	1	Bacteria;WS3;unclassified;unclassified;unclassified;unclassified;
442	0	2	0	Bacteria;unclassified;unclassified;unclassified;unclassified;unclassified;
443	0	0	2	Bacteria;Proteobacteria;Gammaproteobacteria;Alteromonadales;Colwelliaceae;Colwellia;
444	0	0	2	Bacteria;Bacteroidetes;Flavobacteria;Flavobacteriales;Flavobacteriaceae;unclassified;
445	0	2	0	Bacteria;Proteobacteria;Alphaproteobacteria;Rhodobacterales;Rhodobacteraceae;unclassified;
446	0	0	2	Bacteria;unclassified;unclassified;unclassified;unclassified;unclassified;
447	0	2	0	Bacteria;unclassified;unclassified;unclassified;unclassified;unclassified;
448	0	2	0	Bacteria;Bacteroidetes;unclassified;unclassified;unclassified;unclassified;
449	0	0	2	Bacteria;unclassified;unclassified;unclassified;unclassified;unclassified;
450	0	2	0	Bacteria;Bacteroidetes;unclassified;unclassified;unclassified;unclassified;
451	0	2	0	Bacteria;Proteobacteria;Deltaproteobacteria;Desulfobacterales;Desulfobulbaceae;unclassified;
452	0	0	2	Bacteria;unclassified;unclassified;unclassified;unclassified;unclassified;
453	0	2	0	Bacteria;unclassified;unclassified;unclassified;unclassified;unclassified;
454	0	2	0	Bacteria;Proteobacteria;Gammaproteobacteria;unclassified;unclassified;unclassified;
455	0	2	0	Bacteria;Bacteroidetes;unclassified;unclassified;unclassified;unclassified;
456	0	0	2	Bacteria;unclassified;unclassified;unclassified;unclassified;unclassified;
457	0	2	0	Bacteria;unclassified;unclassified;unclassified;unclassified;unclassified;

458	0	0	2	Bacteria;Proteobacteria;Gammaproteobacteria;unclassified;unclassified;unclassified;
459	0	1	1	Bacteria;Acidobacteria;Acidobacteria;Acidobacteriales;Acidobacteriaceae;Gp23
460	0	0	2	Bacteria;Proteobacteria;Gammaproteobacteria;unclassified;unclassified;unclassified;
461	0	0	2	Bacteria;Proteobacteria;Alphaproteobacteria;unclassified;unclassified;unclassified;
462	0	0	2	Bacteria;Bacteroidetes;unclassified;unclassified;unclassified;unclassified;
463	0	1	1	Bacteria;Proteobacteria;Deltaproteobacteria;Desulfobacterales;unclassified;unclassified;
464	0	2	0	Bacteria;Bacteroidetes;unclassified;unclassified;unclassified;unclassified;
465	0	2	0	Bacteria;unclassified;unclassified;unclassified;unclassified;unclassified;
466	0	0	2	Bacteria;Firmicutes;Bacilli;Bacillales;Staphylococcaceae;Staphylococcus;
467	0	0	2	Bacteria;Bacteroidetes;unclassified;unclassified;unclassified;unclassified;
468	0	0	2	Bacteria;Proteobacteria;Alphaproteobacteria;unclassified;unclassified;unclassified;
469	0	0	2	Bacteria;Proteobacteria;Gammaproteobacteria;unclassified;unclassified;unclassified;
470	0	2	0	Bacteria;unclassified;unclassified;unclassified;unclassified;unclassified;
471	0	0	2	Bacteria;Proteobacteria;Gammaproteobacteria;unclassified;unclassified;unclassified;
472	0	0	2	Bacteria;Bacteroidetes;unclassified;unclassified;unclassified;unclassified;
473	0	2	0	Bacteria;Proteobacteria;Deltaproteobacteria;unclassified;unclassified;unclassified;
474	0	1	1	Bacteria;Proteobacteria;Betaproteobacteria;unclassified;unclassified;unclassified;
475	0	2	0	Bacteria;Proteobacteria;Epsilonproteobacteria;unclassified;unclassified;unclassified;
476	1	0	1	Bacteria;Proteobacteria;Alphaproteobacteria;Rhizobiales;Methylocystaceae;Terasakiella;
477	0	1	1	Bacteria;Proteobacteria;Deltaproteobacteria;Desulfobacterales;Desulfobacteraceae;Desulfobacula;
478	0	1	1	Bacteria;Actinobacteria;Actinobacteria;Actinomycetales;unclassified;unclassified;
479	0	2	0	Bacteria;Proteobacteria;Gammaproteobacteria;unclassified;unclassified;unclassified;
480	0	2	0	Bacteria;Proteobacteria;Deltaproteobacteria;Desulfobacterales;Desulfobulbaceae;unclassified;
481	0	0	2	Bacteria;unclassified;unclassified;unclassified;unclassified;unclassified;
482	0	2	0	Bacteria;Proteobacteria;Gammaproteobacteria;Thiotrichales;Thiotrichaceae;Cocleimonas;
483	0	2	0	Bacteria;Proteobacteria;Deltaproteobacteria;Desulfobacterales;Desulfobacteraceae;Desulfobacterium;
484	0	2	0	Bacteria;Bacteroidetes;Flavobacteria;Flavobacteriales;Flavobacteriaceae;Eudoraea;
485	0	2	0	Bacteria;Proteobacteria;Deltaproteobacteria;Desulfuromonadales;unclassified;unclassified;
486	0	2	0	Bacteria;unclassified;unclassified;unclassified;unclassified;unclassified;
487	1	0	1	Bacteria;Proteobacteria;Alphaproteobacteria;Rhodobacterales;Rhodobacteraceae;unclassified;
488	2	0	0	Bacteria;Proteobacteria;Gammaproteobacteria;unclassified;unclassified;unclassified;
489	0	0	2	Bacteria;Proteobacteria;Deltaproteobacteria;Desulfuromonadales;Desulfuromonadaceae;unclassified;
490	0	2	0	Bacteria;unclassified;unclassified;unclassified;unclassified;unclassified;
491	2	0	0	Bacteria;Proteobacteria;Betaproteobacteria;Burkholderiales;Comamonadaceae;Acidovorax;
492	0	0	2	Bacteria;Proteobacteria;Gammaproteobacteria;unclassified;unclassified;unclassified;
493	0	2	0	Bacteria;unclassified;unclassified;unclassified;unclassified;unclassified;
494	0	2	0	Bacteria;Bacteroidetes;unclassified;unclassified;unclassified;unclassified;
495	0	0	2	Bacteria;Proteobacteria;Epsilonproteobacteria;Campylobacterales;Helicobacteraceae;Sulfurimonas;
496	0	0	2	Bacteria;Proteobacteria;Deltaproteobacteria;unclassified;unclassified;unclassified;
497	2	0	0	Bacteria;Bacteroidetes;Flavobacteria;Flavobacteriales;Flavobacteriaceae;Flavobacterium;
498	0	1	1	Bacteria;Proteobacteria;Gammaproteobacteria;Oceanospirillales;Oceanospirillaceae;Spongiispira;
499	0	0	2	Bacteria;Proteobacteria;Gammaproteobacteria;unclassified;unclassified;unclassified;

500	0	0	2	Bacteria;Spirochaetes;Spirochaetes;Spirochaetales;Spirochaetaceae;Spirochaeta;
501	0	0	2	Bacteria;Firmicutes;Bacilli;Lactobacillales;Streptococcaceae;Lactococcus;
502	0	0	2	Bacteria;Bacteroidetes;unclassified;unclassified;unclassified;unclassified;
503	0	0	2	Bacteria;Proteobacteria;unclassified;unclassified;unclassified;unclassified;
504	0	2	0	Bacteria;Proteobacteria;Gammaproteobacteria;unclassified;unclassified;unclassified;
505	0	0	2	Bacteria;Proteobacteria;Deltaproteobacteria;Myxococcales;Nannocystaceae;unclassified;
506	0	0	2	Bacteria;unclassified;unclassified;unclassified;unclassified;unclassified;
507	0	0	2	Bacteria;Proteobacteria;Deltaproteobacteria;Desulfobacterales;Desulfobacteraceae;unclassified;
508	0	1	1	Bacteria;Bacteroidetes;unclassified;unclassified;unclassified;unclassified;
509	0	0	2	Bacteria;Proteobacteria;Deltaproteobacteria;Desulfuromonadales;unclassified;unclassified;
510	1	0	1	Bacteria;Proteobacteria;Gammaproteobacteria;Vibrionales;Vibrionaceae;Vibrio;
511	0	2	0	Bacteria;unclassified;unclassified;unclassified;unclassified;unclassified;
512	0	1	0	Bacteria;Proteobacteria;Alphaproteobacteria;unclassified;unclassified;unclassified;
513	0	1	0	Bacteria;Proteobacteria;Gammaproteobacteria;unclassified;unclassified;unclassified;
514	0	0	1	Bacteria;Firmicutes;Bacilli;Lactobacillales;Streptococcaceae;Lactococcus;
515	0	0	1	Bacteria;Proteobacteria;Alphaproteobacteria;Rhizobiales;unclassified;unclassified;
516	0	1	0	Bacteria;Proteobacteria;Deltaproteobacteria;unclassified;unclassified;unclassified;
517	0	0	1	Bacteria;Proteobacteria;Gammaproteobacteria;unclassified;unclassified;unclassified;
518	0	0	1	Bacteria;Proteobacteria;Alphaproteobacteria;Rhodospirillales;Rhodospirillaceae;Pelagibius;
519	0	1	0	Bacteria;Proteobacteria;Betaproteobacteria;unclassified;unclassified;unclassified;
520	0	1	0	Bacteria;Proteobacteria;Alphaproteobacteria;Rhodobacterales;Rhodobacteraceae;Hwanghaeicola;
521	1	0	0	Bacteria;Proteobacteria;Gammaproteobacteria;unclassified;unclassified;unclassified;
522	0	1	0	Bacteria;Proteobacteria;Epsilonproteobacteria;Campylobacterales;unclassified;unclassified;
523	0	1	0	Bacteria;Proteobacteria;Gammaproteobacteria;unclassified;unclassified;unclassified;
524	1	0	0	Bacteria;Proteobacteria;Betaproteobacteria;unclassified;unclassified;unclassified;
525	0	1	0	Bacteria;Proteobacteria;Alphaproteobacteria;Rhodospirillales;Rhodospirillaceae;unclassified;
526	0	1	0	Bacteria;Proteobacteria;Alphaproteobacteria;Rhodobacterales;Rhodobacteraceae;unclassified;
527	0	0	1	Bacteria;Proteobacteria;Deltaproteobacteria;Myxococcales;unclassified;unclassified;
528	0	1	0	Bacteria;Proteobacteria;Alphaproteobacteria;Rhodobacterales;Rhodobacteraceae;unclassified;
529	0	0	1	Bacteria;Bacteroidetes;Sphingobacteria;Sphingobacteriales;unclassified;unclassified;
530	0	1	0	Bacteria;Proteobacteria;Deltaproteobacteria;unclassified;unclassified;unclassified;
531	0	1	0	Bacteria;Proteobacteria;Betaproteobacteria;unclassified;unclassified;unclassified;
532	0	1	0	Bacteria;Proteobacteria;Deltaproteobacteria;unclassified;unclassified;unclassified;
533	0	0	1	Bacteria;Proteobacteria;Alphaproteobacteria;Rhodobacterales;Rhodobacteraceae;unclassified;
534	1	0	0	Bacteria;Proteobacteria;Gammaproteobacteria;Enterobacteriales;Enterobacteriaceae;Pantoea;
535	0	0	1	Bacteria;Proteobacteria;Gammaproteobacteria;unclassified;unclassified;unclassified;
536	0	1	0	Bacteria;Proteobacteria;Gammaproteobacteria;unclassified;unclassified;unclassified;
537	0	1	0	Bacteria;Proteobacteria;Alphaproteobacteria;Rhizobiales;unclassified;unclassified;
538	0	0	1	Bacteria;Proteobacteria;Gammaproteobacteria;Vibrionales;Vibrionaceae;Photobacterium;
539	0	1	0	Bacteria;unclassified;unclassified;unclassified;unclassified;unclassified;
540	0	0	1	Bacteria;Proteobacteria;Alphaproteobacteria;Rhodobacterales;Rhodobacteraceae;unclassified;
541	0	1	0	Bacteria;Proteobacteria;Gammaproteobacteria;unclassified;unclassified;unclassified;

542	0	0	1	Bacteria;Proteobacteria;Alphaproteobacteria;unclassified;unclassified;unclassified;
543	0	1	0	Bacteria;Proteobacteria;Deltaproteobacteria;Syntrophobacterales;unclassified;unclassified;
544	0	1	0	Bacteria;Proteobacteria;Gammaproteobacteria;unclassified;unclassified;unclassified;
545	0	1	0	Bacteria;Proteobacteria;Alphaproteobacteria;unclassified;unclassified;unclassified;
546	0	0	1	Bacteria;Proteobacteria;Gammaproteobacteria;unclassified;unclassified;unclassified;
547	0	1	0	Bacteria;Bacteroidetes;Sphingobacteria;Sphingobacteriales;Flammeovirgaceae;unclassified;
548	0	0	1	Bacteria;Proteobacteria;Gammaproteobacteria;unclassified;unclassified;unclassified;
549	0	0	1	Bacteria;Proteobacteria;Deltaproteobacteria;Myxococcales;unclassified;unclassified;
550	0	1	0	Bacteria;Bacteroidetes;unclassified;unclassified;unclassified;unclassified;
551	0	0	1	Bacteria;unclassified;unclassified;unclassified;unclassified;unclassified;
552	0	1	0	Bacteria;Proteobacteria;Deltaproteobacteria;Desulfovibrionales;Desulfovibrionaceae;Desulfovibrio;
553	0	0	1	Bacteria;Proteobacteria;Deltaproteobacteria;Myxococcales;Haliangiaceae;Haliangium;
554	0	1	0	Bacteria;unclassified;unclassified;unclassified;unclassified;unclassified;
555	0	1	0	Bacteria;Proteobacteria;Deltaproteobacteria;unclassified;unclassified;unclassified;
556	0	1	0	Bacteria;Bacteroidetes;Flavobacteria;Flavobacteriales;Flavobacteriaceae;Robiginitalea;
557	0	1	0	Bacteria;Proteobacteria;Deltaproteobacteria;Desulfobacterales;Desulfobacteraceae;unclassified;
558	0	1	0	Bacteria;Bacteroidetes;unclassified;unclassified;unclassified;unclassified;
559	0	1	0	Bacteria;Bacteroidetes;unclassified;unclassified;unclassified;unclassified;
560	0	1	0	Bacteria;Bacteroidetes;unclassified;unclassified;unclassified;unclassified;
561	0	0	1	Bacteria;WS3;unclassified;unclassified;unclassified;unclassified;
562	0	1	0	Bacteria;unclassified;unclassified;unclassified;unclassified;unclassified;
563	0	1	0	Bacteria;Proteobacteria;Gammaproteobacteria;unclassified;unclassified;unclassified;
564	0	0	1	Bacteria;Proteobacteria;Gammaproteobacteria;unclassified;unclassified;unclassified;
565	0	1	0	Bacteria;Proteobacteria;Deltaproteobacteria;Desulfobacterales;Desulfobacteraceae;unclassified;
566	0	1	0	Bacteria;Bacteroidetes;unclassified;unclassified;unclassified;unclassified;
567	0	0	1	Bacteria;Proteobacteria;Alphaproteobacteria;Rhodospirillales;Rhodospirillaceae;unclassified;
568	0	0	1	Bacteria;Proteobacteria;Epsilonproteobacteria;Campylobacterales;Campylobacteraceae;Arcobacter;
569	0	1	0	Bacteria;unclassified;unclassified;unclassified;unclassified;unclassified;
570	0	1	0	Bacteria;Proteobacteria;Gammaproteobacteria;unclassified;unclassified;unclassified;
571	0	0	1	Bacteria;Proteobacteria;Gammaproteobacteria;unclassified;unclassified;unclassified;
572	0	1	0	Bacteria;Proteobacteria;Alphaproteobacteria;unclassified;unclassified;unclassified;
573	0	1	0	Bacteria;Proteobacteria;Gammaproteobacteria;Chromatiales;Chromatiaceae;unclassified;
574	0	0	1	Bacteria;unclassified;unclassified;unclassified;unclassified;unclassified;
575	0	0	1	Bacteria;Proteobacteria;Gammaproteobacteria;Vibrionales;Vibrionaceae;Vibrio;
576	0	0	1	Bacteria;Proteobacteria;Gammaproteobacteria;unclassified;unclassified;unclassified;
577	0	0	1	Bacteria;unclassified;unclassified;unclassified;unclassified;unclassified;
578	0	0	1	Bacteria;Bacteroidetes;unclassified;unclassified;unclassified;unclassified;
579	0	1	0	Bacteria;Proteobacteria;Deltaproteobacteria;unclassified;unclassified;unclassified;
580	0	0	1	Bacteria;Proteobacteria;Gammaproteobacteria;unclassified;unclassified;unclassified;
581	0	0	1	Bacteria;Proteobacteria;Alphaproteobacteria;unclassified;unclassified;unclassified;
582	0	0	1	Bacteria;unclassified;unclassified;unclassified;unclassified;unclassified;
583	0	1	0	Bacteria;Proteobacteria;Alphaproteobacteria;Rhizobiales;Rhizobiaceae;Rhizobium;

584	0	0	1	Bacteria;Proteobacteria;Gammaproteobacteria;unclassified;unclassified;unclassified;
585	0	0	1	Bacteria;Proteobacteria;Deltaproteobacteria;Myxococcales;unclassified;unclassified;
586	0	0	1	Bacteria;Proteobacteria;Alphaproteobacteria;Rhodobacterales;Rhodobacteraceae;unclassified;
587	0	0	1	Bacteria;Proteobacteria;Alphaproteobacteria;unclassified;unclassified;unclassified;
588	0	1	0	Bacteria;unclassified;unclassified;unclassified;unclassified;unclassified;
589	0	0	1	Bacteria;Actinobacteria;Actinobacteria;unclassified;unclassified;unclassified;
590	0	0	1	Bacteria;Proteobacteria;Alphaproteobacteria;Rhodobacterales;Rhodobacteraceae;unclassified;
591	0	0	1	Bacteria;Proteobacteria;Deltaproteobacteria;unclassified;unclassified;unclassified;
592	0	1	0	Bacteria;Bacteroidetes;unclassified;unclassified;unclassified;unclassified;
593	0	0	1	Bacteria;Proteobacteria;Deltaproteobacteria;Desulfovibrionales;unclassified;unclassified;
594	0	0	1	Bacteria;Proteobacteria;Alphaproteobacteria;Rhodospirillales;Rhodospirillaceae;unclassified;
595	0	0	1	Bacteria;Bacteroidetes;Flavobacteria;Flavobacteriales;unclassified;unclassified;
596	0	1	0	Bacteria;unclassified;unclassified;unclassified;unclassified;unclassified;
597	0	0	1	Bacteria;Bacteroidetes;Flavobacteria;Flavobacteriales;Flavobacteriaceae;unclassified;
598	0	1	0	Bacteria;Proteobacteria;Betaproteobacteria;Rhodocyclales;Rhodocyclaceae;unclassified;
599	0	0	1	Bacteria;Proteobacteria;Alphaproteobacteria;Rhizobiales;unclassified;unclassified;
600	0	1	0	Bacteria;unclassified;unclassified;unclassified;unclassified;unclassified;
601	0	1	0	Bacteria;Bacteroidetes;unclassified;unclassified;unclassified;unclassified;
602	0	0	1	Bacteria;unclassified;unclassified;unclassified;unclassified;unclassified;
603	0	1	0	Bacteria;Bacteroidetes;unclassified;unclassified;unclassified;unclassified;
604	0	1	0	Bacteria;Bacteroidetes;Sphingobacteria;Sphingobacteriales;Flammeovirgaceae;unclassified;
605	0	1	0	Bacteria;Bacteroidetes;unclassified;unclassified;unclassified;unclassified;
606	0	0	1	Bacteria;Proteobacteria;Deltaproteobacteria;unclassified;unclassified;unclassified;
607	0	0	1	Bacteria;Proteobacteria;Alphaproteobacteria;Rhodobacterales;Rhodobacteraceae;unclassified;
608	0	0	1	Bacteria;Proteobacteria;Deltaproteobacteria;Myxococcales;Polyangiaceae;unclassified;
609	0	0	1	Bacteria;Proteobacteria;Alphaproteobacteria;unclassified;unclassified;unclassified;
610	0	1	0	Bacteria;Proteobacteria;Epsilonproteobacteria;unclassified;unclassified;unclassified;
611	0	0	1	Bacteria;Firmicutes;Clostridia;Clostridiales;Peptostreptococcaceae;unclassified;
612	0	1	0	Bacteria;Bacteroidetes;unclassified;unclassified;unclassified;unclassified;
613	0	0	1	Bacteria;Proteobacteria;Gammaproteobacteria;unclassified;unclassified;unclassified;
614	0	1	0	Bacteria;Acidobacteria;Acidobacteria;Acidobacteriales;Acidobacteriaceae;Gp23
615	0	1	0	Bacteria;Proteobacteria;unclassified;unclassified;unclassified;unclassified;
616	0	0	1	Bacteria;Firmicutes;Bacilli;Lactobacillales;Streptococcaceae;Streptococcus;
617	0	1	0	Bacteria;unclassified;unclassified;unclassified;unclassified;unclassified;
618	0	0	1	Bacteria;Fusobacteria;Fusobacteria;Fusobacteriales;Fusobacteriaceae;unclassified;
619	0	0	1	Bacteria;Bacteroidetes;Sphingobacteria;Sphingobacteriales;unclassified;unclassified;
620	0	1	0	Bacteria;Proteobacteria;Deltaproteobacteria;unclassified;unclassified;unclassified;
621	0	0	1	Bacteria;Proteobacteria;Deltaproteobacteria;unclassified;unclassified;unclassified;
622	0	0	1	Bacteria;Bacteroidetes;unclassified;unclassified;unclassified;unclassified;
623	0	1	0	Bacteria;unclassified;unclassified;unclassified;unclassified;unclassified;
624	0	1	0	Bacteria;Bacteroidetes;unclassified;unclassified;unclassified;unclassified;
625	0	0	1	Bacteria;Proteobacteria;Gammaproteobacteria;Alteromonadales;unclassified;unclassified;

626	0	0	1	Bacteria;Proteobacteria;Alphaproteobacteria;Rhodobacterales;Rhodobacteraceae;Sulfitobacter;
627	0	0	1	Bacteria;Proteobacteria;Gammaproteobacteria;unclassified;unclassified;unclassified;
628	0	1	0	Bacteria;Proteobacteria;Deltaproteobacteria;Bdellovibrionales;Bacteriovoracaceae;unclassified;
629	0	1	0	Bacteria;Proteobacteria;Gammaproteobacteria;unclassified;unclassified;unclassified;
630	0	1	0	Bacteria;unclassified;unclassified;unclassified;unclassified;unclassified;
631	0	0	1	Bacteria;Bacteroidetes;unclassified;unclassified;unclassified;unclassified;
632	0	0	1	Bacteria;Proteobacteria;Gammaproteobacteria;Vibrionales;Vibrionaceae;unclassified;
633	0	1	0	Bacteria;unclassified;unclassified;unclassified;unclassified;unclassified;
634	0	1	0	Bacteria;Bacteroidetes;unclassified;unclassified;unclassified;unclassified;
635	0	0	1	Bacteria;Proteobacteria;Deltaproteobacteria;unclassified;unclassified;unclassified;
636	0	1	0	Bacteria;Proteobacteria;Deltaproteobacteria;Desulfobacterales;Desulfobacteraceae;unclassified;
637	0	1	0	Bacteria;Proteobacteria;Deltaproteobacteria;unclassified;unclassified;unclassified;
638	0	0	1	Bacteria;Proteobacteria;Gammaproteobacteria;unclassified;unclassified;unclassified;
639	0	0	1	Bacteria;Proteobacteria;unclassified;unclassified;unclassified;unclassified;
640	0	1	0	Bacteria;Proteobacteria;Deltaproteobacteria;Myxococcales;unclassified;unclassified;
641	0	1	0	Bacteria;Proteobacteria;Alphaproteobacteria;Sneathiellales;Sneathiellaceae;Sneathiella;
642	0	1	0	Bacteria;Proteobacteria;Alphaproteobacteria;Rhizobiales;unclassified;unclassified;
643	0	0	1	Bacteria;Proteobacteria;Alphaproteobacteria;unclassified;unclassified;unclassified;
644	0	1	0	Bacteria;unclassified;unclassified;unclassified;unclassified;unclassified;
645	0	0	1	Bacteria;Firmicutes;Clostridia;Clostridiales;Clostridiaceae_1;Sarcina;
646	0	1	0	Bacteria;Proteobacteria;Alphaproteobacteria;unclassified;unclassified;unclassified;
647	0	1	0	Bacteria;Bacteroidetes;unclassified;unclassified;unclassified;unclassified;
648	0	1	0	Bacteria;unclassified;unclassified;unclassified;unclassified;unclassified;
649	0	0	1	Bacteria;Proteobacteria;Alphaproteobacteria;unclassified;unclassified;unclassified;
650	0	1	0	Bacteria;Bacteroidetes;unclassified;unclassified;unclassified;unclassified;
651	0	0	1	Bacteria;Bacteroidetes;unclassified;unclassified;unclassified;unclassified;
652	0	0	1	Bacteria;Proteobacteria;Alphaproteobacteria;Sphingomonadales;Sphingomonadaceae;Sphingopyxis;
653	0	1	0	Bacteria;Bacteroidetes;unclassified;unclassified;unclassified;unclassified;
654	0	0	1	Bacteria;Proteobacteria;Deltaproteobacteria;Myxococcales;unclassified;unclassified;
655	0	0	1	Bacteria;unclassified;unclassified;unclassified;unclassified;unclassified;
656	0	0	1	Bacteria;Proteobacteria;Gammaproteobacteria;unclassified;unclassified;unclassified;
657	0	1	0	Bacteria;Proteobacteria;Alphaproteobacteria;unclassified;unclassified;unclassified;
658	0	1	0	Bacteria;Bacteroidetes;unclassified;unclassified;unclassified;unclassified;
659	0	0	1	Bacteria;Proteobacteria;Gammaproteobacteria;unclassified;unclassified;unclassified;
660	0	1	0	Bacteria;Bacteroidetes;unclassified;unclassified;unclassified;unclassified;
661	0	1	0	Bacteria;Bacteroidetes;unclassified;unclassified;unclassified;unclassified;
662	0	0	1	Bacteria;Proteobacteria;Gammaproteobacteria;unclassified;unclassified;unclassified;
663	0	0	1	Bacteria;Proteobacteria;Gammaproteobacteria;unclassified;unclassified;unclassified;
664	0	0	1	Bacteria;Fusobacteria;Fusobacteria;Fusobacteriales;unclassified;unclassified;
665	0	1	0	Bacteria;Bacteroidetes;unclassified;unclassified;unclassified;unclassified;
666	0	0	1	Bacteria;Proteobacteria;Gammaproteobacteria;unclassified;unclassified;unclassified;
667	0	0	1	Bacteria;Proteobacteria;Epsilonproteobacteria;Campylobacterales;Campylobacteraceae;Arcobacter;

668	0	0	1	Bacteria;Proteobacteria;Gammaproteobacteria;unclassified;unclassified;unclassified;
669	0	0	1	Bacteria;Firmicutes;Clostridia;Clostridiales;Ruminococcaceae;Clostridium_III;
670	0	0	1	Bacteria;Proteobacteria;Alphaproteobacteria;Rhizobiales;unclassified;unclassified;
671	0	0	1	Bacteria;Proteobacteria;Deltaproteobacteria;Bdellovibrionales;Bacteriovoracaceae;unclassified;
672	0	1	0	Bacteria;unclassified;unclassified;unclassified;unclassified;unclassified;
673	0	0	1	Bacteria;Proteobacteria;Deltaproteobacteria;Desulfobacterales;Desulfobacteraceae;unclassified;
674	0	0	1	Bacteria;Bacteroidetes;unclassified;unclassified;unclassified;unclassified;
675	0	1	0	Bacteria;unclassified;unclassified;unclassified;unclassified;unclassified;
676	0	1	0	Bacteria;unclassified;unclassified;unclassified;unclassified;unclassified;
677	0	0	1	Bacteria;Proteobacteria;Gammaproteobacteria;Pseudomonadales;Pseudomonadaceae;Pseudomonas;
678	0	0	1	Bacteria;Proteobacteria;unclassified;unclassified;unclassified;unclassified;
679	0	0	1	Bacteria;Proteobacteria;Alphaproteobacteria;unclassified;unclassified;unclassified;
680	0	0	1	Bacteria;Proteobacteria;Gammaproteobacteria;unclassified;unclassified;unclassified;
681	0	0	1	Bacteria;Proteobacteria;unclassified;unclassified;unclassified;unclassified;
682	0	1	0	Bacteria;Bacteroidetes;unclassified;unclassified;unclassified;unclassified;
683	0	0	1	Bacteria;Proteobacteria;Alphaproteobacteria;unclassified;unclassified;unclassified;
684	0	0	1	Bacteria;Proteobacteria;Betaproteobacteria;unclassified;unclassified;unclassified;
685	0	0	1	Bacteria;Actinobacteria;Actinobacteria;unclassified;unclassified;unclassified;
686	0	0	1	Bacteria;Proteobacteria;Deltaproteobacteria;Myxococcales;unclassified;unclassified;
687	0	1	0	Bacteria;Acidobacteria;Acidobacteria;Acidobacteriales;Acidobacteriaceae;Gp24
688	0	1	0	Bacteria;Proteobacteria;Alphaproteobacteria;Sphingomonadales;Erythrobacteraceae;Altererythrobacter;
689	0	1	0	Bacteria;unclassified;unclassified;unclassified;unclassified;unclassified;
690	0	1	0	Bacteria;unclassified;unclassified;unclassified;unclassified;unclassified;
691	0	1	0	Bacteria;Proteobacteria;Alphaproteobacteria;Rhodobacterales;Rhodobacteraceae;unclassified;
692	0	1	0	Bacteria;unclassified;unclassified;unclassified;unclassified;unclassified;
693	0	1	0	Bacteria;Proteobacteria;Deltaproteobacteria;Bdellovibrionales;Bacteriovoracaceae;Bacteriovorax;
694	0	1	0	Bacteria;Bacteroidetes;unclassified;unclassified;unclassified;unclassified;
695	0	1	0	Bacteria;Proteobacteria;Gammaproteobacteria;unclassified;unclassified;unclassified;
696	0	1	0	Bacteria;Proteobacteria;Deltaproteobacteria;Desulfobacterales;Desulfobacteraceae;unclassified;
697	0	1	0	Bacteria;Bacteroidetes;Flavobacteria;Flavobacteriales;Flavobacteriaceae;unclassified;
698	0	1	0	Bacteria;Bacteroidetes;unclassified;unclassified;unclassified;unclassified;
699	0	1	0	Bacteria;Proteobacteria;Deltaproteobacteria;Desulfobacterales;Desulfobacteraceae;unclassified;
700	0	1	0	Bacteria;unclassified;unclassified;unclassified;unclassified;unclassified;
701	0	1	0	Bacteria;unclassified;unclassified;unclassified;unclassified;unclassified;
702	0	1	0	Bacteria;Proteobacteria;Deltaproteobacteria;Desulfobacterales;Desulfobulbaceae;unclassified;
703	0	1	0	Bacteria;Bacteroidetes;unclassified;unclassified;unclassified;unclassified;
704	0	1	0	Bacteria;Bacteroidetes;unclassified;unclassified;unclassified;unclassified;
705	0	1	0	Bacteria;Bacteroidetes;unclassified;unclassified;unclassified;unclassified;
706	0	1	0	Bacteria;Proteobacteria;Deltaproteobacteria;unclassified;unclassified;unclassified;
707	0	1	0	Bacteria;Firmicutes;Clostridia;Clostridiales;Ruminococcaceae;unclassified;
708	0	1	0	Bacteria;Proteobacteria;Gammaproteobacteria;unclassified;unclassified;unclassified;
709	0	1	0	Bacteria;unclassified;unclassified;unclassified;unclassified;unclassified;

710	0	1	0	Bacteria;Proteobacteria;Gammaproteobacteria;unclassified;unclassified;unclassified;
711	0	1	0	Bacteria;unclassified;unclassified;unclassified;unclassified;unclassified;
712	0	1	0	Bacteria;Bacteroidetes;unclassified;unclassified;unclassified;unclassified;
713	0	1	0	Bacteria;Bacteroidetes;unclassified;unclassified;unclassified;unclassified;
714	0	1	0	Bacteria;Bacteroidetes;unclassified;unclassified;unclassified;unclassified;
715	0	1	0	Bacteria;Bacteroidetes;unclassified;unclassified;unclassified;unclassified;
716	0	1	0	Bacteria;Bacteroidetes;Flavobacteria;Flavobacteriales;Flavobacteriaceae;Lutibacter;
717	0	1	0	Bacteria;Proteobacteria;Alphaproteobacteria;Rhodobacterales;Rhodobacteraceae;unclassified;
718	0	1	0	Bacteria;unclassified;unclassified;unclassified;unclassified;unclassified;
719	0	1	0	Bacteria;Spirochaetes;Spirochaetes;Spirochaetales;Spirochaetaceae;Spirochaeta;
720	0	1	0	Bacteria;Proteobacteria;Gammaproteobacteria;Chromatiales;Chromatiaceae;unclassified;
721	0	1	0	Bacteria;Bacteroidetes;unclassified;unclassified;unclassified;unclassified;
722	0	1	0	Bacteria;Proteobacteria;Deltaproteobacteria;Desulfobacterales;Desulfobacteraceae;unclassified;
723	0	1	0	Bacteria;Bacteroidetes;unclassified;unclassified;unclassified;unclassified;
724	0	1	0	Bacteria;Proteobacteria;unclassified;unclassified;unclassified;unclassified;
725	0	1	0	Bacteria;Spirochaetes;Spirochaetes;Spirochaetales;Spirochaetaceae;unclassified;
726	0	1	0	Bacteria;Proteobacteria;Deltaproteobacteria;unclassified;unclassified;unclassified;
727	0	1	0	Bacteria;Proteobacteria;Deltaproteobacteria;unclassified;unclassified;unclassified;
728	0	1	0	Bacteria;Proteobacteria;Deltaproteobacteria;Desulfobacterales;Desulfobulbaceae;unclassified;
729	0	1	0	Bacteria;Bacteroidetes;Bacteroidia;Marinilabiliales;Prolixibacteraceae;Prolixibacter;
730	0	1	0	Bacteria;Proteobacteria;Gammaproteobacteria;Chromatiales;unclassified;unclassified;
731	0	1	0	Bacteria;Bacteroidetes;unclassified;unclassified;unclassified;unclassified;
732	0	1	0	Bacteria;Proteobacteria;Gammaproteobacteria;unclassified;unclassified;unclassified;
733	0	1	0	Bacteria;Firmicutes;Clostridia;Clostridiales;unclassified;unclassified;
734	0	1	0	Bacteria;Proteobacteria;Gammaproteobacteria;unclassified;unclassified;unclassified;
735	0	1	0	Bacteria;Proteobacteria;Alphaproteobacteria;Rhodobacterales;Rhodobacteraceae;Jannaschia;
736	0	1	0	Bacteria;Proteobacteria;Gammaproteobacteria;unclassified;unclassified;unclassified;
737	0	1	0	Bacteria;Proteobacteria;Deltaproteobacteria;Desulfobacterales;unclassified;unclassified;
738	0	1	0	Bacteria;Bacteroidetes;Sphingobacteria;Sphingobacteriales;unclassified;unclassified;
739	0	1	0	Bacteria;Proteobacteria;Deltaproteobacteria;Myxococcales;unclassified;unclassified;
740	0	1	0	Bacteria;Actinobacteria;Actinobacteria;unclassified;unclassified;unclassified;
741	0	1	0	Bacteria;unclassified;unclassified;unclassified;unclassified;unclassified;
742	0	1	0	Bacteria;Bacteroidetes;unclassified;unclassified;unclassified;unclassified;
743	0	1	0	Bacteria;unclassified;unclassified;unclassified;unclassified;unclassified;
744	0	1	0	Bacteria;unclassified;unclassified;unclassified;unclassified;unclassified;
745	0	1	0	Bacteria;Proteobacteria;Gammaproteobacteria;unclassified;unclassified;unclassified;
746	0	1	0	Bacteria;Bacteroidetes;unclassified;unclassified;unclassified;unclassified;
747	0	1	0	Bacteria;Proteobacteria;Deltaproteobacteria;unclassified;unclassified;unclassified;
748	0	1	0	Bacteria;Proteobacteria;Deltaproteobacteria;Desulfobacterales;Desulfobacteraceae;unclassified;
749	0	1	0	Bacteria;Proteobacteria;unclassified;unclassified;unclassified;unclassified;
750	0	1	0	Bacteria;Actinobacteria;Actinobacteria;unclassified;unclassified;unclassified;
751	0	1	0	Bacteria;unclassified;unclassified;unclassified;unclassified;unclassified;

752	0	1	0	Bacteria;Bacteroidetes;unclassified;unclassified;unclassified;unclassified;
753	0	1	0	Bacteria;unclassified;unclassified;unclassified;unclassified;unclassified;
754	0	1	0	Bacteria;Proteobacteria;Alphaproteobacteria;Rhodobacterales;Rhodobacteraceae;unclassified;
755	0	1	0	Bacteria;Proteobacteria;unclassified;unclassified;unclassified;unclassified;
756	0	1	0	Bacteria;Bacteroidetes;unclassified;unclassified;unclassified;unclassified;
757	0	1	0	Bacteria;Proteobacteria;Epsilonproteobacteria;unclassified;unclassified;unclassified;
758	0	1	0	Bacteria;unclassified;unclassified;unclassified;unclassified;unclassified;
759	0	1	0	Bacteria;unclassified;unclassified;unclassified;unclassified;unclassified;
760	0	1	0	Bacteria;Proteobacteria;Epsilonproteobacteria;Campylobacterales;Helicobacteraceae;Sulfurovum;
761	0	1	0	Bacteria;unclassified;unclassified;unclassified;unclassified;unclassified;
762	0	1	0	Bacteria;Proteobacteria;Alphaproteobacteria;Rhodobacterales;Rhodobacteraceae;unclassified;
763	0	1	0	Bacteria;Bacteroidetes;Flavobacteria;Flavobacteriales;Flavobacteriaceae;Actibacter;
764	0	1	0	Bacteria;Proteobacteria;Gammaproteobacteria;Oceanospirillales;Hahellaceae;Hahella;
765	0	1	0	Bacteria;Proteobacteria;Deltaproteobacteria;Bdellovibrionales;Bacteriovoracaceae;Bacteriovorax;
766	0	1	0	Bacteria;Bacteroidetes;unclassified;unclassified;unclassified;unclassified;
767	0	1	0	Bacteria;Proteobacteria;unclassified;unclassified;unclassified;unclassified;
768	0	1	0	Bacteria;Proteobacteria;unclassified;unclassified;unclassified;unclassified;
769	0	1	0	Bacteria;Proteobacteria;Deltaproteobacteria;Desulfobacterales;Desulfobacteraceae;unclassified;
770	0	1	0	Bacteria;Proteobacteria;Deltaproteobacteria;unclassified;unclassified;unclassified;
771	0	1	0	Bacteria;Bacteroidetes;unclassified;unclassified;unclassified;unclassified;
772	0	1	0	Bacteria;Firmicutes;Bacilli;Bacillales;Paenibacillaceae_1;Paenibacillus;
773	0	1	0	Bacteria;Proteobacteria;Gammaproteobacteria;Alteromonadales;Alteromonadaceae;Haliea;
774	0	1	0	Bacteria;unclassified;unclassified;unclassified;unclassified;unclassified;
775	0	1	0	Bacteria;Proteobacteria;Alphaproteobacteria;unclassified;unclassified;unclassified;
776	0	1	0	Bacteria;Proteobacteria;Deltaproteobacteria;Desulfobacterales;Desulfobacteraceae;unclassified;
777	0	1	0	Bacteria;Proteobacteria;Gammaproteobacteria;unclassified;unclassified;unclassified;
778	0	1	0	Bacteria;Proteobacteria;Gammaproteobacteria;Oceanospirillales;Hahellaceae;Hahella;
779	0	1	0	Bacteria;unclassified;unclassified;unclassified;unclassified;unclassified;
780	0	1	0	Bacteria;Bacteroidetes;unclassified;unclassified;unclassified;unclassified;
781	0	1	0	Bacteria;Proteobacteria;Epsilonproteobacteria;Campylobacterales;Campylobacteraceae;Arcobacter;
782	0	1	0	Bacteria;Proteobacteria;Deltaproteobacteria;unclassified;unclassified;unclassified;
783	0	1	0	Bacteria;Bacteroidetes;unclassified;unclassified;unclassified;unclassified;
784	0	1	0	Bacteria;Proteobacteria;unclassified;unclassified;unclassified;unclassified;
785	0	1	0	Bacteria;Bacteroidetes;unclassified;unclassified;unclassified;unclassified;
786	0	1	0	Bacteria;Bacteroidetes;unclassified;unclassified;unclassified;unclassified;
787	0	1	0	Bacteria;Bacteroidetes;Flavobacteria;Flavobacteriales;Cryomorphaceae;unclassified;
788	0	1	0	Bacteria;Proteobacteria;Deltaproteobacteria;Desulfobacterales;Desulfobacteraceae;unclassified;
789	0	1	0	Bacteria;Acidobacteria;Acidobacteria;Acidobacteriales;Acidobacteriaceae;Gp23
790	0	1	0	Bacteria;Proteobacteria;Epsilonproteobacteria;Campylobacterales;Campylobacteraceae;Arcobacter;
791	0	1	0	Bacteria;unclassified;unclassified;unclassified;unclassified;unclassified;
792	0	1	0	Bacteria;Proteobacteria;Gammaproteobacteria;unclassified;unclassified;unclassified;
793	0	1	0	Bacteria;unclassified;unclassified;unclassified;unclassified;unclassified;

794	0	1	0	Bacteria;unclassified;unclassified;unclassified;unclassified;unclassified;
795	0	1	0	Bacteria;Proteobacteria;Deltaproteobacteria;Desulfuromonadales;Desulfuromonadaceae;Desulfuromusa;
796	0	1	0	Bacteria;unclassified;unclassified;unclassified;unclassified;unclassified;
797	0	1	0	Bacteria;unclassified;unclassified;unclassified;unclassified;unclassified;
798	0	1	0	Bacteria;Proteobacteria;Epsilonproteobacteria;Campylobacterales;Campylobacteraceae;Arcobacter;
799	0	1	0	Bacteria;Acidobacteria;Acidobacteria;Acidobacteriales;Acidobacteriaceae;Gp23
800	0	1	0	Bacteria;Proteobacteria;Gammaproteobacteria;unclassified;unclassified;unclassified;
801	0	1	0	Bacteria;Bacteroidetes;unclassified;unclassified;unclassified;unclassified;
802	0	1	0	Bacteria;Proteobacteria;unclassified;unclassified;unclassified;unclassified;
803	0	1	0	Bacteria;unclassified;unclassified;unclassified;unclassified;unclassified;
804	0	1	0	Bacteria;Actinobacteria;Actinobacteria;Actinomycetales;unclassified;unclassified;
805	0	1	0	Bacteria;Proteobacteria;Deltaproteobacteria;Desulfobacterales;Desulfobulbaceae;Desulfopila;
806	0	1	0	Bacteria;Proteobacteria;Deltaproteobacteria;Desulfobacterales;Desulfobacteraceae;unclassified;
807	0	1	0	Bacteria;Proteobacteria;Alphaproteobacteria;Rhizobiales;unclassified;unclassified;
808	0	1	0	Bacteria;Bacteroidetes;unclassified;unclassified;unclassified;unclassified;
809	0	1	0	Bacteria;unclassified;unclassified;unclassified;unclassified;unclassified;
810	0	1	0	Bacteria;Proteobacteria;Alphaproteobacteria;Rhodobacterales;Rhodobacteraceae;unclassified;
811	0	1	0	Bacteria;Proteobacteria;Deltaproteobacteria;Myxococcales;unclassified;unclassified;
812	0	1	0	Bacteria;Proteobacteria;Alphaproteobacteria;Rhodospirillales;Rhodospirillaceae;unclassified;
813	0	1	0	Bacteria;unclassified;unclassified;unclassified;unclassified;unclassified;
814	0	1	0	Bacteria;unclassified;unclassified;unclassified;unclassified;unclassified;
815	0	1	0	Bacteria;Proteobacteria;Deltaproteobacteria;Desulfobacterales;Desulfobacteraceae;unclassified;
816	0	1	0	Bacteria;Proteobacteria;Deltaproteobacteria;unclassified;unclassified;unclassified;
817	0	1	0	Bacteria;Proteobacteria;Deltaproteobacteria;Desulfobacterales;Desulfobacteraceae;unclassified;
818	0	1	0	Bacteria;Bacteroidetes;unclassified;unclassified;unclassified;unclassified;
819	0	1	0	Bacteria;Acidobacteria;Acidobacteria;Acidobacteriales;Acidobacteriaceae;Gp22
820	0	1	0	Bacteria;Proteobacteria;Deltaproteobacteria;Desulfobacterales;Desulfobacteraceae;unclassified;
821	0	1	0	Bacteria;Proteobacteria;Deltaproteobacteria;Desulfobacterales;Desulfobacteraceae;unclassified;
822	0	1	0	Bacteria;Proteobacteria;Deltaproteobacteria;Desulfobacterales;Desulfobacteraceae;unclassified;
823	0	1	0	Bacteria;Bacteroidetes;unclassified;unclassified;unclassified;unclassified;
824	0	1	0	Bacteria;Bacteroidetes;unclassified;unclassified;unclassified;unclassified;
825	0	1	0	Bacteria;Proteobacteria;Deltaproteobacteria;unclassified;unclassified;unclassified;
826	0	1	0	Bacteria;Proteobacteria;Gammaproteobacteria;unclassified;unclassified;unclassified;
827	0	1	0	Bacteria;WS3;unclassified;unclassified;unclassified;unclassified;
828	0	1	0	Bacteria;Proteobacteria;Gammaproteobacteria;unclassified;unclassified;unclassified;
829	0	1	0	Bacteria;Proteobacteria;Deltaproteobacteria;Desulfobacterales;Desulfobacteraceae;unclassified;
830	0	1	0	Bacteria;Bacteroidetes;unclassified;unclassified;unclassified;unclassified;
831	0	1	0	Bacteria;Proteobacteria;Alphaproteobacteria;Rhizobiales;unclassified;unclassified;
832	0	1	0	Bacteria;Proteobacteria;Gammaproteobacteria;unclassified;unclassified;unclassified;
833	0	1	0	Bacteria;unclassified;unclassified;unclassified;unclassified;unclassified;
834	0	1	0	Bacteria;unclassified;unclassified;unclassified;unclassified;unclassified;
835	0	1	0	Bacteria;Bacteroidetes;unclassified;unclassified;unclassified;unclassified;

836	0	1	0	Bacteria;unclassified;unclassified;unclassified;unclassified;unclassified;
837	0	1	0	Bacteria;Proteobacteria;Gammaproteobacteria;unclassified;unclassified;unclassified;
838	0	1	0	Bacteria;unclassified;unclassified;unclassified;unclassified;unclassified;
839	0	1	0	Bacteria;Proteobacteria;Alphaproteobacteria;unclassified;unclassified;unclassified;
840	0	1	0	Bacteria;Bacteroidetes;unclassified;unclassified;unclassified;unclassified;
841	0	1	0	Bacteria;Bacteroidetes;unclassified;unclassified;unclassified;unclassified;
842	0	1	0	Bacteria;unclassified;unclassified;unclassified;unclassified;unclassified;
843	0	1	0	Bacteria;unclassified;unclassified;unclassified;unclassified;unclassified;
844	0	1	0	Bacteria;Proteobacteria;Deltaproteobacteria;Desulfobacterales;Desulfobacteraceae;unclassified;
845	0	1	0	Bacteria;unclassified;unclassified;unclassified;unclassified;unclassified;
846	0	1	0	Bacteria;unclassified;unclassified;unclassified;unclassified;unclassified;
847	0	1	0	Bacteria;Proteobacteria;Gammaproteobacteria;Oceanospirillales;Alcanovoracaceae;Marinicella;
848	0	1	0	Bacteria;Proteobacteria;Gammaproteobacteria;unclassified;unclassified;unclassified;
849	0	1	0	Bacteria;Firmicutes;unclassified;unclassified;unclassified;unclassified;
850	0	1	0	Bacteria;Proteobacteria;unclassified;unclassified;unclassified;unclassified;
851	0	1	0	Bacteria;Bacteroidetes;unclassified;unclassified;unclassified;unclassified;
852	0	1	0	Bacteria;unclassified;unclassified;unclassified;unclassified;unclassified;
853	0	1	0	Bacteria;Bacteroidetes;unclassified;unclassified;unclassified;unclassified;
854	0	1	0	Bacteria;unclassified;unclassified;unclassified;unclassified;unclassified;
855	0	1	0	Bacteria;unclassified;unclassified;unclassified;unclassified;unclassified;
856	0	1	0	Bacteria;unclassified;unclassified;unclassified;unclassified;unclassified;
857	0	1	0	Bacteria;unclassified;unclassified;unclassified;unclassified;unclassified;
858	0	1	0	Bacteria;Acidobacteria;Acidobacteria;Acidobacteriales;Acidobacteriaceae;Gp21
859	0	1	0	Bacteria;Proteobacteria;Gammaproteobacteria;Oceanospirillales;Halomonadaceae;Halomonas;
860	0	1	0	Bacteria;Verrucomicrobia;Opitutae;Puniceicoccales;Puniceicoccaceae;Coraliomargarita;
861	0	1	0	Bacteria;Proteobacteria;Gammaproteobacteria;unclassified;unclassified;unclassified;
862	0	1	0	Bacteria;Proteobacteria;Deltaproteobacteria;Desulfobacterales;Desulfobacteraceae;unclassified;
863	0	1	0	Bacteria;Proteobacteria;Gammaproteobacteria;unclassified;unclassified;unclassified;
864	0	1	0	Bacteria;unclassified;unclassified;unclassified;unclassified;unclassified;
865	0	1	0	Bacteria;unclassified;unclassified;unclassified;unclassified;unclassified;
866	0	1	0	Bacteria;Bacteroidetes;unclassified;unclassified;unclassified;unclassified;
867	0	1	0	Bacteria;Proteobacteria;Alphaproteobacteria;Rhodobacterales;Rhodobacteraceae;unclassified;
868	0	1	0	Bacteria;Proteobacteria;Deltaproteobacteria;Desulfovibrionales;Desulfovibrionaceae;Desulfovibrio;
869	0	1	0	Bacteria;Spirochaetes;Spirochaetes;Spirochaetales;Spirochaetaceae;Spirochaeta;
870	0	1	0	Bacteria;unclassified;unclassified;unclassified;unclassified;unclassified;
871	0	1	0	Bacteria;unclassified;unclassified;unclassified;unclassified;unclassified;
872	0	1	0	Bacteria;Acidobacteria;Acidobacteria;Acidobacteriales;Acidobacteriaceae;Gp23
873	0	1	0	Bacteria;Proteobacteria;Alphaproteobacteria;unclassified;unclassified;unclassified;
874	0	1	0	Bacteria;unclassified;unclassified;unclassified;unclassified;unclassified;
875	0	1	0	Bacteria;Proteobacteria;Deltaproteobacteria;Desulfobacterales;Desulfobacteraceae;Desulfobacter;
876	0	1	0	Bacteria;Acidobacteria;Acidobacteria;Acidobacteriales;Acidobacteriaceae;Gp22
877	0	1	0	Bacteria;unclassified;unclassified;unclassified;unclassified;unclassified;

878	0	1	0	Bacteria;Proteobacteria;Alphaproteobacteria;Sphingomonadales;unclassified;unclassified;
879	0	1	0	Bacteria;Proteobacteria;unclassified;unclassified;unclassified;unclassified;
880	0	1	0	Bacteria;Proteobacteria;Alphaproteobacteria;Rhodobacterales;Rhodobacteraceae;Roseisalinus;
881	0	1	0	Bacteria;Bacteroidetes;unclassified;unclassified;unclassified;unclassified;
882	0	1	0	Bacteria;Proteobacteria;Gammaproteobacteria;Chromatiales;Chromatiaceae;unclassified;
883	0	1	0	Bacteria;Proteobacteria;Gammaproteobacteria;unclassified;unclassified;unclassified;
884	0	1	0	Bacteria;Bacteroidetes;unclassified;unclassified;unclassified;unclassified;
885	0	1	0	Bacteria;Proteobacteria;Deltaproteobacteria;Desulfobacterales;Desulfobacteraceae;unclassified;
886	0	1	0	Bacteria;Proteobacteria;Gammaproteobacteria;unclassified;unclassified;unclassified;
887	0	1	0	Bacteria;Proteobacteria;Gammaproteobacteria;Chromatiales;Chromatiaceae;unclassified;
888	0	1	0	Bacteria;Proteobacteria;unclassified;unclassified;unclassified;unclassified;
889	0	1	0	Bacteria;Bacteroidetes;unclassified;unclassified;unclassified;unclassified;
890	0	1	0	Bacteria;Proteobacteria;Deltaproteobacteria;Desulfobacterales;Desulfobacteraceae;unclassified;
891	0	1	0	Bacteria;Proteobacteria;Gammaproteobacteria;Oceanospirillales;Alcanovoracaceae;Marinicella;
892	0	1	0	Bacteria;Proteobacteria;unclassified;unclassified;unclassified;unclassified;
893	0	1	0	Bacteria;Acidobacteria;Acidobacteria;Acidobacteriales;Acidobacteriaceae;Gp10
894	0	1	0	Bacteria;Proteobacteria;Deltaproteobacteria;unclassified;unclassified;unclassified;
895	0	1	0	Bacteria;Bacteroidetes;unclassified;unclassified;unclassified;unclassified;
896	0	1	0	Bacteria;Proteobacteria;Gammaproteobacteria;unclassified;unclassified;unclassified;
897	0	1	0	Bacteria;Acidobacteria;Acidobacteria;Acidobacteriales;Acidobacteriaceae;Gp3
898	0	1	0	Bacteria;unclassified;unclassified;unclassified;unclassified;unclassified;
899	0	1	0	Bacteria;unclassified;unclassified;unclassified;unclassified;unclassified;
900	0	1	0	Bacteria;Proteobacteria;Gammaproteobacteria;unclassified;unclassified;unclassified;
901	0	1	0	Bacteria;Proteobacteria;Deltaproteobacteria;Desulfobacterales;Desulfobacteraceae;Desulfobacterium;
902	0	1	0	Bacteria;Actinobacteria;Actinobacteria;Actinomycetales;unclassified;unclassified;
903	0	1	0	Bacteria;Bacteroidetes;Sphingobacteria;Sphingobacteriales;unclassified;unclassified;
904	0	1	0	Bacteria;Bacteroidetes;unclassified;unclassified;unclassified;unclassified;
905	0	1	0	Bacteria;Acidobacteria;Acidobacteria;Acidobacteriales;Acidobacteriaceae;Gp10
906	0	1	0	Bacteria;Proteobacteria;Alphaproteobacteria;Rhizobiales;Aurantimonadaceae;unclassified;
907	0	1	0	Bacteria;unclassified;unclassified;unclassified;unclassified;unclassified;
908	0	1	0	Bacteria;unclassified;unclassified;unclassified;unclassified;unclassified;
909	0	1	0	Bacteria;Firmicutes;Clostridia;Clostridiales;unclassified;unclassified;
910	0	1	0	Bacteria;Proteobacteria;Deltaproteobacteria;unclassified;unclassified;unclassified;
911	0	1	0	Bacteria;Proteobacteria;Deltaproteobacteria;unclassified;unclassified;unclassified;
912	0	1	0	Bacteria;Actinobacteria;Actinobacteria;unclassified;unclassified;unclassified;
913	0	1	0	Bacteria;Firmicutes;Bacilli;Bacillales;Bacillaceae_2;unclassified;
914	0	1	0	Bacteria;unclassified;unclassified;unclassified;unclassified;unclassified;
915	0	1	0	Bacteria;Proteobacteria;Gammaproteobacteria;unclassified;unclassified;unclassified;
916	0	1	0	Bacteria;Bacteroidetes;unclassified;unclassified;unclassified;unclassified;
917	0	1	0	Bacteria;Verrucomicrobia;unclassified;unclassified;unclassified;unclassified;
918	0	1	0	Bacteria;Proteobacteria;Deltaproteobacteria;Desulfobacterales;Desulfobacteraceae;unclassified;
919	0	1	0	Bacteria;Proteobacteria;Gammaproteobacteria;Xanthomonadales;Xanthomonadaceae;Luteibacter;

920	0	1	0	Bacteria;Proteobacteria;Gammaproteobacteria;unclassified;unclassified;unclassified;
921	0	1	0	Bacteria;Acidobacteria;Acidobacteria;Acidobacteriales;Acidobacteriaceae;Gp3
922	0	1	0	Bacteria;Proteobacteria;Deltaproteobacteria;Desulfobacterales;Desulfobacteraceae;unclassified;
923	0	1	0	Bacteria;Bacteroidetes;unclassified;unclassified;unclassified;unclassified;
924	0	1	0	Bacteria;unclassified;unclassified;unclassified;unclassified;unclassified;
925	0	1	0	Bacteria;Proteobacteria;Alphaproteobacteria;unclassified;unclassified;unclassified;
926	0	1	0	Bacteria;Proteobacteria;Deltaproteobacteria;Desulfovibrionales;Desulfovibrionaceae;unclassified;
927	0	1	0	Bacteria;Proteobacteria;Deltaproteobacteria;Desulfobacterales;Desulfobacteraceae;Desulfobacter;
928	0	1	0	Bacteria;Bacteroidetes;unclassified;unclassified;unclassified;unclassified;
929	0	1	0	Bacteria;Firmicutes;unclassified;unclassified;unclassified;unclassified;
930	0	1	0	Bacteria;Proteobacteria;Deltaproteobacteria;unclassified;unclassified;unclassified;
931	0	1	0	Bacteria;unclassified;unclassified;unclassified;unclassified;unclassified;
932	0	1	0	Bacteria;Bacteroidetes;Flavobacteria;Flavobacteriales;Flavobacteriaceae;Gramella;
933	0	1	0	Bacteria;Acidobacteria;Acidobacteria;Acidobacteriales;Acidobacteriaceae;Gp21
934	0	1	0	Bacteria;Firmicutes;Clostridia;Clostridiales;Ruminococcaceae;unclassified;
935	0	1	0	Bacteria;unclassified;unclassified;unclassified;unclassified;unclassified;
936	0	1	0	Bacteria;Proteobacteria;Gammaproteobacteria;unclassified;unclassified;unclassified;
937	0	1	0	Bacteria;Acidobacteria;Acidobacteria;Acidobacteriales;Acidobacteriaceae;Gp10
938	0	1	0	Bacteria;unclassified;unclassified;unclassified;unclassified;unclassified;
939	0	1	0	Bacteria;Proteobacteria;unclassified;unclassified;unclassified;unclassified;
940	0	1	0	Bacteria;Bacteroidetes;Flavobacteria;Flavobacteriales;Flavobacteriaceae;unclassified;
941	0	1	0	Bacteria;unclassified;unclassified;unclassified;unclassified;unclassified;
942	0	1	0	Bacteria;unclassified;unclassified;unclassified;unclassified;unclassified;
943	0	1	0	Bacteria;Proteobacteria;Deltaproteobacteria;Myxococcales;unclassified;unclassified;
944	0	1	0	Bacteria;Proteobacteria;Deltaproteobacteria;Desulfobacterales;Desulfobacteraceae;unclassified;
945	0	1	0	Bacteria;unclassified;unclassified;unclassified;unclassified;unclassified;
946	0	1	0	Bacteria;Proteobacteria;Gammaproteobacteria;unclassified;unclassified;unclassified;
947	0	1	0	Bacteria;Bacteroidetes;Bacteroidia;Bacteroidales;Porphyromonadaceae;unclassified;
948	0	1	0	Bacteria;Proteobacteria;Deltaproteobacteria;unclassified;unclassified;unclassified;
949	0	1	0	Bacteria;Firmicutes;Clostridia;Clostridiales;unclassified;unclassified;
950	0	1	0	Bacteria;unclassified;unclassified;unclassified;unclassified;unclassified;
951	0	1	0	Bacteria;Proteobacteria;unclassified;unclassified;unclassified;unclassified;
952	0	1	0	Bacteria;Proteobacteria;Deltaproteobacteria;Desulfobacterales;Desulfobacteraceae;Desulfobacula;
953	0	1	0	Bacteria;Bacteroidetes;Flavobacteria;Flavobacteriales;Cryomorphaceae;unclassified;
954	0	1	0	Bacteria;Proteobacteria;unclassified;unclassified;unclassified;unclassified;
955	0	1	0	Bacteria;Proteobacteria;Gammaproteobacteria;unclassified;unclassified;unclassified;
956	0	1	0	Bacteria;Bacteroidetes;unclassified;unclassified;unclassified;unclassified;
957	0	1	0	Bacteria;Proteobacteria;Deltaproteobacteria;Myxococcales;unclassified;unclassified;
958	0	1	0	Bacteria;WS3;unclassified;unclassified;unclassified;unclassified;
959	0	1	0	Bacteria;unclassified;unclassified;unclassified;unclassified;unclassified;
960	0	1	0	Bacteria;unclassified;unclassified;unclassified;unclassified;unclassified;
961	0	1	0	Bacteria;Acidobacteria;Acidobacteria;Acidobacteriales;Acidobacteriaceae;Gp10

962	0	1	0	Bacteria;Proteobacteria;Alphaproteobacteria;Rhodobacterales;Rhodobacteraceae;unclassified;
963	0	1	0	Bacteria;Proteobacteria;Deltaproteobacteria;unclassified;unclassified;unclassified;
964	0	1	0	Bacteria;unclassified;unclassified;unclassified;unclassified;unclassified;
965	0	1	0	Bacteria;Firmicutes;Clostridia;Clostridiales;Lachnospiraceae;unclassified;
966	0	1	0	Bacteria;Bacteroidetes;unclassified;unclassified;unclassified;unclassified;
967	0	1	0	Bacteria;Proteobacteria;Deltaproteobacteria;Desulfobacterales;Desulfobacteraceae;unclassified;
968	0	1	0	Bacteria;Proteobacteria;Gammaproteobacteria;unclassified;unclassified;unclassified;
969	0	1	0	Bacteria;Firmicutes;Clostridia;Clostridiales;unclassified;unclassified;
970	0	0	1	Bacteria;Proteobacteria;Gammaproteobacteria;Enterobacteriales;Enterobacteriaceae;Serratia;
971	0	0	1	Bacteria;Proteobacteria;Deltaproteobacteria;Desulfobacterales;Desulfobacteraceae;unclassified;
972	0	1	0	Bacteria;Proteobacteria;Deltaproteobacteria;Desulfobacterales;Desulfobacteraceae;unclassified;
973	0	1	0	Bacteria;Bacteroidetes;unclassified;unclassified;unclassified;unclassified;
974	0	1	0	Bacteria;Proteobacteria;Gammaproteobacteria;unclassified;unclassified;unclassified;
975	0	1	0	Bacteria;Proteobacteria;Gammaproteobacteria;Alteromonadales;Alteromonadaceae;Marinobacterium;
976	0	1	0	Bacteria;Firmicutes;Bacilli;Bacillales;Paenibacillaceae_1;unclassified;
977	0	1	0	Bacteria;Proteobacteria;Gammaproteobacteria;unclassified;unclassified;unclassified;
978	0	1	0	Bacteria;Proteobacteria;Deltaproteobacteria;Desulfobacterales;Desulfobacteraceae;Desulfosarcina;
979	0	1	0	Bacteria;unclassified;unclassified;unclassified;unclassified;unclassified;
980	0	1	0	Bacteria;Proteobacteria;Deltaproteobacteria;unclassified;unclassified;unclassified;
981	0	1	0	Bacteria;Proteobacteria;Deltaproteobacteria;Desulfobacterales;Desulfobulbaceae;Desulfopila;
982	0	1	0	Bacteria;Firmicutes;Clostridia;unclassified;unclassified;unclassified;
983	0	1	0	Bacteria;Proteobacteria;Gammaproteobacteria;Cellvibrionales;Halieaceae;Congregibacter;
984	0	1	0	Bacteria;Bacteroidetes;unclassified;unclassified;unclassified;unclassified;
985	0	1	0	Bacteria;unclassified;unclassified;unclassified;unclassified;unclassified;
986	1	0	0	Bacteria;Proteobacteria;unclassified;unclassified;unclassified;unclassified;
987	0	1	0	Bacteria;Bacteroidetes;unclassified;unclassified;unclassified;unclassified;
988	0	1	0	Bacteria;Bacteroidetes;unclassified;unclassified;unclassified;unclassified;
989	1	0	0	Bacteria;Proteobacteria;Alphaproteobacteria;Rhodospirillales;Rhodospirillaceae;unclassified;
990	1	0	0	Bacteria;Bacteroidetes;Sphingobacteria;Sphingobacteriales;Flammeovirgaceae;Marinoscillum;
991	0	1	0	Bacteria;unclassified;unclassified;unclassified;unclassified;unclassified;
992	0	1	0	Bacteria;Proteobacteria;Gammaproteobacteria;unclassified;unclassified;unclassified;
993	0	1	0	Bacteria;unclassified;unclassified;unclassified;unclassified;unclassified;
994	0	1	0	Bacteria;Bacteroidetes;unclassified;unclassified;unclassified;unclassified;
995	0	1	0	Bacteria;Bacteroidetes;unclassified;unclassified;unclassified;unclassified;
996	0	1	0	Bacteria;Proteobacteria;Alphaproteobacteria;Rhodobacterales;Rhodobacteraceae;unclassified;
997	1	0	0	Bacteria;Bacteroidetes;Sphingobacteria;Sphingobacteriales;unclassified;unclassified;
998	1	0	0	Bacteria;Proteobacteria;Gammaproteobacteria;Salinisphaerales;Salinisphaeraceae;Salinisphaera;
999	1	0	0	Bacteria;Proteobacteria;Deltaproteobacteria;Desulfuromonadales;unclassified;unclassified;
1000	0	0	1	Bacteria;unclassified;unclassified;unclassified;unclassified;unclassified;
1001	0	1	0	Bacteria;Proteobacteria;Epsilonproteobacteria;Campylobacterales;unclassified;unclassified;
1002	0	1	0	Bacteria;unclassified;unclassified;unclassified;unclassified;unclassified;
1003	1	0	0	Bacteria;Proteobacteria;Alphaproteobacteria;Caulobacterales;Caulobacteraceae;Phenylobacterium;

1004	1	0	0	Bacteria;unclassified;unclassified;unclassified;unclassified;unclassified;
1005	0	0	1	Bacteria;Bacteroidetes;Flavobacteria;Flavobacteriales;Flavobacteriaceae;unclassified;
1006	0	1	0	Bacteria;Proteobacteria;Gammaproteobacteria;unclassified;unclassified;unclassified;
1007	0	0	1	Bacteria;Proteobacteria;Deltaproteobacteria;Myxococcales;unclassified;unclassified;
1008	0	1	0	Bacteria;unclassified;unclassified;unclassified;unclassified;unclassified;
1009	0	0	1	Bacteria;unclassified;unclassified;unclassified;unclassified;unclassified;
1010	0	1	0	Bacteria;Verrucomicrobia;Opitutae;unclassified;unclassified;unclassified;
1011	0	1	0	Bacteria;unclassified;unclassified;unclassified;unclassified;unclassified;
1012	0	0	1	Bacteria;unclassified;unclassified;unclassified;unclassified;unclassified;
1013	0	0	1	Bacteria;Acidobacteria;Holophagae;Acanthopleuribacterales;Acanthopleuribacteraceae;Acanthopleuribacter;
1014	0	0	1	Bacteria;Proteobacteria;Epsilonproteobacteria;Campylobacterales;unclassified;unclassified;
1015	0	0	1	Bacteria;Proteobacteria;Gammaproteobacteria;Xanthomonadales;Sinobacteraceae;Steroidobacter;
1016	0	1	0	Bacteria;Proteobacteria;Gammaproteobacteria;Chromatiales;Chromatiaceae;unclassified;
1017	0	1	0	Bacteria;Bacteroidetes;unclassified;unclassified;unclassified;unclassified;
1018	0	0	1	Bacteria;Proteobacteria;Alphaproteobacteria;unclassified;unclassified;unclassified;
1019	0	0	1	Bacteria;Acidobacteria;Acidobacteria;Acidobacteriales;Acidobacteriaceae;Gp10
1020	0	1	0	Bacteria;Firmicutes;Clostridia;Clostridiales;unclassified;unclassified;
1021	0	1	0	Bacteria;Bacteroidetes;unclassified;unclassified;unclassified;unclassified;
1022	0	0	1	Bacteria;unclassified;unclassified;unclassified;unclassified;unclassified;
1023	0	1	0	Bacteria;Proteobacteria;Deltaproteobacteria;Desulfobacterales;Desulfobacteraceae;unclassified;
1024	0	0	1	Bacteria;Proteobacteria;Deltaproteobacteria;Desulfobacterales;Desulfobacteraceae;unclassified;
1025	0	1	0	Bacteria;Proteobacteria;unclassified;unclassified;unclassified;unclassified;
1026	0	0	1	Bacteria;Proteobacteria;Gammaproteobacteria;Chromatiales;unclassified;unclassified;
1027	0	0	1	Bacteria;Proteobacteria;Betaproteobacteria;Burkholderiales;unclassified;unclassified;
1028	0	0	1	Bacteria;unclassified;unclassified;unclassified;unclassified;unclassified;
1029	0	0	1	Bacteria;Bacteroidetes;unclassified;unclassified;unclassified;unclassified;
1030	0	0	1	Bacteria;Proteobacteria;unclassified;unclassified;unclassified;unclassified;
1031	0	1	0	Bacteria;Bacteroidetes;unclassified;unclassified;unclassified;unclassified;
1032	0	0	1	Bacteria;Proteobacteria;Gammaproteobacteria;unclassified;unclassified;unclassified;
1033	0	1	0	Bacteria;unclassified;unclassified;unclassified;unclassified;unclassified;
1034	0	1	0	Bacteria;unclassified;unclassified;unclassified;unclassified;unclassified;
1035	0	0	1	Bacteria;Bacteroidetes;Flavobacteria;Flavobacteriales;unclassified;unclassified;
1036	0	0	1	Bacteria;unclassified;unclassified;unclassified;unclassified;unclassified;
1037	0	1	0	Bacteria;Bacteroidetes;unclassified;unclassified;unclassified;unclassified;
1038	0	1	0	Bacteria;unclassified;unclassified;unclassified;unclassified;unclassified;
1039	0	0	1	Bacteria;unclassified;unclassified;unclassified;unclassified;unclassified;
1040	0	0	1	Bacteria;Proteobacteria;Deltaproteobacteria;unclassified;unclassified;unclassified;
1041	0	1	0	Bacteria;Bacteroidetes;Sphingobacteria;Sphingobacteriales;unclassified;unclassified;
1042	0	1	0	Bacteria;Bacteroidetes;unclassified;unclassified;unclassified;unclassified;
1043	0	1	0	Bacteria;unclassified;unclassified;unclassified;unclassified;unclassified;
1044	0	0	1	Bacteria;Proteobacteria;Deltaproteobacteria;Desulfuromonadales;unclassified;unclassified;
1045	0	1	0	Bacteria;Proteobacteria;Gammaproteobacteria;unclassified;unclassified;unclassified;

1046	0	1	0	Bacteria;Bacteroidetes;unclassified;unclassified;unclassified;unclassified;
1047	0	1	0	Bacteria;unclassified;unclassified;unclassified;unclassified;unclassified;
1048	0	1	0	Bacteria;unclassified;unclassified;unclassified;unclassified;unclassified;
1049	0	0	1	Bacteria;unclassified;unclassified;unclassified;unclassified;unclassified;
1050	0	0	1	Bacteria;Proteobacteria;unclassified;unclassified;unclassified;unclassified;
1051	0	0	1	Bacteria;unclassified;unclassified;unclassified;unclassified;unclassified;
1052	0	0	1	Bacteria;Proteobacteria;Gammaproteobacteria;Alteromonadales;unclassified;unclassified;
1053	0	0	1	Bacteria;Proteobacteria;Gammaproteobacteria;unclassified;unclassified;unclassified;
1054	0	0	1	Bacteria;Bacteroidetes;unclassified;unclassified;unclassified;unclassified;
1055	0	1	0	Bacteria;Acidobacteria;Acidobacteria;Acidobacteriales;Acidobacteriaceae;Gp10
1056	0	0	1	Bacteria;unclassified;unclassified;unclassified;unclassified;unclassified;
1057	0	0	1	Bacteria;Bacteroidetes;unclassified;unclassified;unclassified;unclassified;
1058	0	0	1	Bacteria;Bacteroidetes;unclassified;unclassified;unclassified;unclassified;
1059	0	0	1	Bacteria;Bacteroidetes;unclassified;unclassified;unclassified;unclassified;
1060	0	0	1	Bacteria;Bacteroidetes;Flavobacteria;Flavobacteriales;Flavobacteriaceae;unclassified;
1061	0	1	0	Bacteria;Proteobacteria;Deltaproteobacteria;Desulfobacterales;Desulfobacteraceae;Desulfobacterium;
1062	0	0	1	Bacteria;Proteobacteria;Gammaproteobacteria;unclassified;unclassified;unclassified;
1063	0	0	1	Bacteria;unclassified;unclassified;unclassified;unclassified;unclassified;
1064	0	0	1	Bacteria;Proteobacteria;Deltaproteobacteria;Bdellovibrionales;Bacteriovoracaceae;Bacteriovorax;
1065	0	0	1	Bacteria;Proteobacteria;Gammaproteobacteria;Xanthomonadales;Xanthomonadaceae;Rhodanobacter;
1066	0	1	0	Bacteria;Proteobacteria;unclassified;unclassified;unclassified;unclassified;
1067	0	1	0	Bacteria;Proteobacteria;Gammaproteobacteria;unclassified;unclassified;unclassified;
1068	0	0	1	Bacteria;unclassified;unclassified;unclassified;unclassified;unclassified;
1069	0	0	1	Bacteria;unclassified;unclassified;unclassified;unclassified;unclassified;
1070	0	0	1	Bacteria;Bacteroidetes;Bacteroidia;Bacteroidales;unclassified;unclassified;
1071	0	0	1	Bacteria;Proteobacteria;unclassified;unclassified;unclassified;unclassified;
1072	0	1	0	Bacteria;Firmicutes;unclassified;unclassified;unclassified;unclassified;
1073	0	1	0	Bacteria;unclassified;unclassified;unclassified;unclassified;unclassified;
1074	0	0	1	Bacteria;Proteobacteria;Gammaproteobacteria;Oceanospirillales;Halomonadaceae;Halomonas;
1075	0	1	0	Bacteria;Proteobacteria;Gammaproteobacteria;unclassified;unclassified;unclassified;
1076	0	0	1	Bacteria;Bacteroidetes;unclassified;unclassified;unclassified;unclassified;
1077	0	1	0	Bacteria;Proteobacteria;Deltaproteobacteria;Desulfobacterales;Desulfobacteraceae;Desulfobacter;
1078	0	0	1	Bacteria;Proteobacteria;Deltaproteobacteria;Desulfobacterales;Desulfobulbaceae;unclassified;
1079	0	0	1	Bacteria;Proteobacteria;Deltaproteobacteria;Desulfobacterales;Desulfobacteraceae;unclassified;
1080	0	0	1	Bacteria;Proteobacteria;Alphaproteobacteria;Rhodobacterales;Rhodobacteraceae;unclassified;
1081	0	1	0	Bacteria;unclassified;unclassified;unclassified;unclassified;unclassified;
1082	0	1	0	Bacteria;Proteobacteria;Deltaproteobacteria;Desulfobacterales;Desulfobacteraceae;Desulfobacula;
1083	1	0	0	Bacteria;Proteobacteria;Gammaproteobacteria;unclassified;unclassified;unclassified;
1084	1	0	0	Bacteria;Proteobacteria;Betaproteobacteria;Burkholderiales;Oxalobacteraceae;unclassified;
1085	0	1	0	Bacteria;Proteobacteria;Gammaproteobacteria;unclassified;unclassified;unclassified;
1086	1	0	0	Bacteria;Proteobacteria;Deltaproteobacteria;unclassified;unclassified;unclassified;
1087	0	1	0	Bacteria;Proteobacteria;Gammaproteobacteria;unclassified;unclassified;unclassified;

1088	1	0	0	Bacteria;Proteobacteria;Gammaproteobacteria;Pseudomonadales;Moraxellaceae;Acinetobacter;
1089	0	1	0	Bacteria;Bacteroidetes;unclassified;unclassified;unclassified;unclassified;
1090	1	0	0	Bacteria;Proteobacteria;unclassified;unclassified;unclassified;unclassified;
1091	1	0	0	Bacteria;Bacteroidetes;Sphingobacteria;Sphingobacteriales;Saprospiraceae;Lewinella;
1092	1	0	0	Bacteria;Proteobacteria;unclassified;unclassified;unclassified;unclassified;
1093	1	0	0	Bacteria;Proteobacteria;Alphaproteobacteria;Rhodobacterales;Rhodobacteraceae;unclassified;
1094	1	0	0	Bacteria;Proteobacteria;Gammaproteobacteria;Alteromonadales;Alteromonadaceae;Marinobacter;
1095	1	0	0	Bacteria;Proteobacteria;Alphaproteobacteria;Rhodobacterales;Rhodobacteraceae;unclassified;
1096	0	1	0	Bacteria;unclassified;unclassified;unclassified;unclassified;unclassified;
1097	1	0	0	Bacteria;Proteobacteria;Alphaproteobacteria;unclassified;unclassified;unclassified;
1098	1	0	0	Bacteria;Proteobacteria;Deltaproteobacteria;Myxococcales;unclassified;unclassified;
1099	0	1	0	Bacteria;Bacteroidetes;Flavobacteria;Flavobacteriales;Flavobacteriaceae;Salinimicrobium;
1100	1	0	0	Bacteria;Proteobacteria;Gammaproteobacteria;unclassified;unclassified;unclassified;
1101	0	1	0	Bacteria;Acidobacteria;Acidobacteria;Acidobacteriales;Acidobacteriaceae;Gp21
1102	0	1	0	Bacteria;unclassified;unclassified;unclassified;unclassified;unclassified;
1103	1	0	0	Bacteria;Proteobacteria;Gammaproteobacteria;unclassified;unclassified;unclassified;
1104	1	0	0	Bacteria;Proteobacteria;Alphaproteobacteria;Rhodobacterales;Rhodobacteraceae;unclassified;
1105	0	1	0	Bacteria;Bacteroidetes;unclassified;unclassified;unclassified;unclassified;
1106	0	1	0	Bacteria;Proteobacteria;Alphaproteobacteria;Rhodobacterales;Rhodobacteraceae;unclassified;
1107	0	1	0	Bacteria;Bacteroidetes;unclassified;unclassified;unclassified;unclassified;
1108	1	0	0	Bacteria;Bacteroidetes;Flavobacteria;Flavobacteriales;Flavobacteriaceae;Chryseobacterium;
1109	1	0	0	Bacteria;Bacteroidetes;Sphingobacteria;Sphingobacteriales;unclassified;unclassified;
1110	1	0	0	Bacteria;Proteobacteria;Gammaproteobacteria;Pseudomonadales;Moraxellaceae;unclassified;
1111	1	0	0	Bacteria;Proteobacteria;Gammaproteobacteria;Alteromonadales;unclassified;unclassified;
1112	0	1	0	Bacteria;unclassified;unclassified;unclassified;unclassified;unclassified;
1113	1	0	0	Bacteria;Bacteroidetes;Flavobacteria;Flavobacteriales;Flavobacteriaceae;unclassified;
1114	0	1	0	Bacteria;Proteobacteria;Gammaproteobacteria;unclassified;unclassified;unclassified;
1115	0	1	0	Bacteria;Proteobacteria;Gammaproteobacteria;unclassified;unclassified;unclassified;
1116	1	0	0	Bacteria;Proteobacteria;Betaproteobacteria;Burkholderiales;Comamonadaceae;unclassified;
1117	1	0	0	Bacteria;Proteobacteria;Betaproteobacteria;Burkholderiales;Oxalobacteraceae;Herminiimonas;
1118	0	1	0	Bacteria;Proteobacteria;Deltaproteobacteria;Desulfobacterales;Desulfobacteraceae;Desulfobacula;
1119	1	0	0	Bacteria;Proteobacteria;Deltaproteobacteria;unclassified;unclassified;unclassified;
1120	1	0	0	Bacteria;Proteobacteria;Betaproteobacteria;Burkholderiales;Alcaligenaceae;Achromobacter;
1121	1	0	0	Bacteria;unclassified;unclassified;unclassified;unclassified;unclassified;
1122	0	1	0	Bacteria;unclassified;unclassified;unclassified;unclassified;unclassified;
1123	1	0	0	Bacteria;Proteobacteria;Gammaproteobacteria;Alteromonadales;Shewanellaceae;Shewanella;
1124	0	1	0	Bacteria;unclassified;unclassified;unclassified;unclassified;unclassified;
1125	1	0	0	Bacteria;Proteobacteria;Alphaproteobacteria;Rhodobacterales;Rhodobacteraceae;unclassified;
1126	1	0	0	Bacteria;Proteobacteria;Deltaproteobacteria;Desulfuromonadales;unclassified;unclassified;
1127	0	1	0	Bacteria;Proteobacteria;Alphaproteobacteria;Rhodobacterales;Rhodobacteraceae;unclassified;
1128	0	1	0	Bacteria;unclassified;unclassified;unclassified;unclassified;unclassified;
1129	0	1	0	Bacteria;unclassified;unclassified;unclassified;unclassified;unclassified;

1130	1	0	0	Bacteria;Proteobacteria;Betaproteobacteria;Burkholderiales;Comamonadaceae;Variovorax;
1131	1	0	0	Bacteria;Proteobacteria;Deltaproteobacteria;unclassified;unclassified;unclassified;
1132	0	1	0	Bacteria;unclassified;unclassified;unclassified;unclassified;unclassified;
1133	1	0	0	Bacteria;Bacteroidetes;Flavobacteria;Flavobacteriales;Flavobacteriaceae;unclassified;
1134	1	0	0	Bacteria;Proteobacteria;Betaproteobacteria;Burkholderiales;Oxalobacteraceae;Herminiimonas;
1135	1	0	0	Bacteria;Proteobacteria;Gammaproteobacteria;Alteromonadales;Pseudoalteromonadaceae;Algicola;
1136	1	0	0	Bacteria;Proteobacteria;Gammaproteobacteria;unclassified;unclassified;unclassified;
1137	1	0	0	Bacteria;Proteobacteria;Gammaproteobacteria;unclassified;unclassified;unclassified;
1138	1	0	0	Bacteria;Proteobacteria;Betaproteobacteria;Burkholderiales;Burkholderiaceae;Polynucleobacter;
1139	0	1	0	Bacteria;unclassified;unclassified;unclassified;unclassified;unclassified;
1140	1	0	0	Bacteria;Verrucomicrobia;Verrucomicrobiae;Verrucomicrobiales;unclassified;unclassified;
1141	0	1	0	Bacteria;Proteobacteria;Gammaproteobacteria;unclassified;unclassified;unclassified;
1142	1	0	0	Bacteria;Firmicutes;Bacilli;Bacillales;Alicyclobacillaceae;Alicyclobacillus;
1143	0	1	0	Bacteria;Proteobacteria;Gammaproteobacteria;Oceanospirillales;Halomonadaceae;Halomonas;
1144	0	1	0	Bacteria;Firmicutes;Clostridia;Clostridiales;Clostridiaceae_1;unclassified;
1145	0	1	0	Bacteria;Firmicutes;unclassified;unclassified;unclassified;unclassified;
1146	1	0	0	Bacteria;Actinobacteria;Actinobacteria;Actinomycetales;Micrococcaceae;Arthrobacter;
1147	1	0	0	Bacteria;Bacteroidetes;unclassified;unclassified;unclassified;unclassified;
1148	1	0	0	Bacteria;Proteobacteria;Gammaproteobacteria;Vibrionales;Vibrionaceae;Vibrio;
1149	1	0	0	Bacteria;Proteobacteria;Gammaproteobacteria;unclassified;unclassified;unclassified;
1150	0	1	0	Bacteria;Proteobacteria;Alphaproteobacteria;unclassified;unclassified;unclassified;
1151	0	1	0	Bacteria;Bacteroidetes;unclassified;unclassified;unclassified;unclassified;
1152	1	0	0	Bacteria;Proteobacteria;Gammaproteobacteria;Thiotrichales;Francisellaceae;Francisella;
1153	1	0	0	Bacteria;Proteobacteria;Gammaproteobacteria;unclassified;unclassified;unclassified;
1154	1	0	0	Bacteria;unclassified;unclassified;unclassified;unclassified;unclassified;
1155	1	0	0	Bacteria;Proteobacteria;Alphaproteobacteria;Caulobacterales;Caulobacteraceae;Brevundimonas;
1156	1	0	0	Bacteria;unclassified;unclassified;unclassified;unclassified;unclassified;
1157	0	1	0	Bacteria;unclassified;unclassified;unclassified;unclassified;unclassified;
1158	1	0	0	Bacteria;Proteobacteria;unclassified;unclassified;unclassified;unclassified;
1159	0	1	0	Bacteria;Proteobacteria;Gammaproteobacteria;unclassified;unclassified;unclassified;
1160	0	1	0	Bacteria;unclassified;unclassified;unclassified;unclassified;unclassified;
1161	1	0	0	Bacteria;Verrucomicrobia;Verrucomicrobiae;Verrucomicrobiales;Verrucomicrobiaceae;unclassified;
1162	0	1	0	Bacteria;Proteobacteria;Betaproteobacteria;Burkholderiales;unclassified;unclassified;
1163	1	0	0	Bacteria;Proteobacteria;Alphaproteobacteria;unclassified;unclassified;unclassified;
1164	1	0	0	Bacteria;Proteobacteria;Alphaproteobacteria;Rhodobacterales;Rhodobacteraceae;Loktanella;
1165	0	1	0	Bacteria;Bacteroidetes;unclassified;unclassified;unclassified;unclassified;
1166	0	0	1	Bacteria;Proteobacteria;Deltaproteobacteria;Desulfobacterales;Desulfobacteraceae;Desulfobacterium;
1167	1	0	0	Bacteria;Bacteroidetes;Flavobacteria;Flavobacteriales;Flavobacteriaceae;Flavobacterium;
1168	1	0	0	Bacteria;Firmicutes;Clostridia;Clostridiales;Clostridiaceae_1;Clostridium_sensu_stricto;
1169	1	0	0	Bacteria;Proteobacteria;unclassified;unclassified;unclassified;unclassified;
1170	1	0	0	Bacteria;Proteobacteria;Gammaproteobacteria;Alteromonadales;Colwelliaceae;Thalassomonas;
1171	0	1	0	Bacteria;Proteobacteria;Deltaproteobacteria;Desulfobacterales;Desulfobacteraceae;unclassified;

1172	0	0	1	Bacteria;Acidobacteria;Acidobacteria;Acidobacteriales;Acidobacteriaceae;Gp4
1173	0	0	1	Bacteria;Proteobacteria;Deltaproteobacteria;unclassified;unclassified;unclassified;
1174	0	0	1	Bacteria;Proteobacteria;Alphaproteobacteria;Rhodobacterales;Rhodobacteraceae;unclassified;
1175	0	1	0	Bacteria;Proteobacteria;Deltaproteobacteria;Desulfobacterales;Desulfobulbaceae;unclassified;
1176	0	0	1	Bacteria;Bacteroidetes;unclassified;unclassified;unclassified;unclassified;
1177	0	1	0	Bacteria;Proteobacteria;Gammaproteobacteria;unclassified;unclassified;unclassified;
1178	0	0	1	Bacteria;Proteobacteria;Alphaproteobacteria;unclassified;unclassified;unclassified;
1179	0	0	1	Bacteria;unclassified;unclassified;unclassified;unclassified;unclassified;
1180	0	0	1	Bacteria;unclassified;unclassified;unclassified;unclassified;unclassified;
1181	0	1	0	Bacteria;WS3;unclassified;unclassified;unclassified;unclassified;
1182	0	0	1	Bacteria;Proteobacteria;Alphaproteobacteria;Caulobacterales;Hyphomonadaceae;unclassified;
1183	0	1	0	Bacteria;Firmicutes;Clostridia;Clostridiales;Eubacteriaceae;Alkalibacter;
1184	0	1	0	Bacteria;Bacteroidetes;unclassified;unclassified;unclassified;unclassified;
1185	0	0	1	Bacteria;Proteobacteria;Gammaproteobacteria;unclassified;unclassified;unclassified;
1186	0	1	0	Bacteria;Acidobacteria;Acidobacteria;Acidobacteriales;Acidobacteriaceae;Gp10
1187	0	0	1	Bacteria;Proteobacteria;Deltaproteobacteria;Desulfobacterales;Desulfobulbaceae;unclassified;
1188	0	0	1	Bacteria;Proteobacteria;Gammaproteobacteria;unclassified;unclassified;unclassified;
1189	0	0	1	Bacteria;Proteobacteria;Epsilonproteobacteria;Campylobacterales;unclassified;unclassified;
1190	0	0	1	Bacteria;Proteobacteria;Gammaproteobacteria;Vibrionales;Vibrionaceae;unclassified;
1191	0	1	0	Bacteria;unclassified;unclassified;unclassified;unclassified;unclassified;
1192	0	1	0	Bacteria;unclassified;unclassified;unclassified;unclassified;unclassified;
1193	0	0	1	Bacteria;Bacteroidetes;unclassified;unclassified;unclassified;unclassified;
1194	0	0	1	Bacteria;Acidobacteria;unclassified;unclassified;unclassified;unclassified;
1195	0	1	0	Bacteria;Bacteroidetes;unclassified;unclassified;unclassified;unclassified;
1196	0	0	1	Bacteria;Proteobacteria;Gammaproteobacteria;unclassified;unclassified;unclassified;
1197	0	1	0	Bacteria;Firmicutes;unclassified;unclassified;unclassified;unclassified;
1198	0	0	1	Bacteria;Proteobacteria;Gammaproteobacteria;unclassified;unclassified;unclassified;
1199	0	0	1	Bacteria;Proteobacteria;Gammaproteobacteria;unclassified;unclassified;unclassified;
1200	0	1	0	Bacteria;Proteobacteria;Gammaproteobacteria;unclassified;unclassified;unclassified;
1201	0	1	0	Bacteria;Bacteroidetes;unclassified;unclassified;unclassified;unclassified;
1202	0	0	1	Bacteria;Firmicutes;Clostridia;Clostridiales;Gracilibacteraceae;Gracilibacter;
1203	0	1	0	Bacteria;Proteobacteria;Deltaproteobacteria;Desulfobacterales;Desulfobacteraceae;unclassified;
1204	0	0	1	Bacteria;Proteobacteria;Gammaproteobacteria;unclassified;unclassified;unclassified;
1205	0	1	0	Bacteria;Proteobacteria;Gammaproteobacteria;unclassified;unclassified;unclassified;
1206	0	0	1	Bacteria;Proteobacteria;Gammaproteobacteria;unclassified;unclassified;unclassified;
1207	0	0	1	Bacteria;Proteobacteria;Gammaproteobacteria;Enterobacteriales;Enterobacteriaceae;Escherichia_Shigella;
1208	0	1	0	Bacteria;unclassified;unclassified;unclassified;unclassified;unclassified;
1209	0	0	1	Bacteria;Acidobacteria;Holophagae;Acanthopleuribacterales;Acanthopleuribacteraceae;Acanthopleuribacter;
1210	0	0	1	Bacteria;unclassified;unclassified;unclassified;unclassified;unclassified;
1211	0	0	1	Bacteria;Proteobacteria;Gammaproteobacteria;unclassified;unclassified;unclassified;
1212	0	0	1	Bacteria;Bacteroidetes;unclassified;unclassified;unclassified;unclassified;
1213	0	0	1	Bacteria;Proteobacteria;Alphaproteobacteria;Rhodospirillales;unclassified;unclassified;

1214	0	0	1	Bacteria;Proteobacteria;Gammaproteobacteria;unclassified;unclassified;unclassified;
1215	0	0	1	Bacteria;Firmicutes;Clostridia;Clostridiales;Ruminococcaceae;unclassified;
1216	0	1	0	Bacteria;Firmicutes;Bacilli;Lactobacillales;Carnobacteriaceae;Alkalibacterium;
1217	0	1	0	Bacteria;Proteobacteria;unclassified;unclassified;unclassified;unclassified;
1218	0	0	1	Bacteria;unclassified;unclassified;unclassified;unclassified;unclassified;
1219	0	0	1	Bacteria;unclassified;unclassified;unclassified;unclassified;unclassified;
1220	0	1	0	Bacteria;Bacteroidetes;Sphingobacteria;Sphingobacteriales;Flammeovirgaceae;Fulvivirga;
1221	0	0	1	Bacteria;Proteobacteria;unclassified;unclassified;unclassified;unclassified;
1222	0	1	0	Bacteria;Firmicutes;Erysipelotrichia;Erysipelotrichales;Erysipelotrichaceae;unclassified;
1223	0	1	0	Bacteria;Proteobacteria;unclassified;unclassified;unclassified;unclassified;
1224	0	1	0	Bacteria;Proteobacteria;Deltaproteobacteria;Desulfobacterales;Desulfobacteraceae;unclassified;
1225	0	1	0	Bacteria;unclassified;unclassified;unclassified;unclassified;unclassified;
1226	0	0	1	Bacteria;Proteobacteria;Gammaproteobacteria;unclassified;unclassified;unclassified;
1227	0	0	1	Bacteria;Proteobacteria;unclassified;unclassified;unclassified;unclassified;
1228	0	1	0	Bacteria;Proteobacteria;Gammaproteobacteria;unclassified;unclassified;unclassified;
1229	0	1	0	Bacteria;Proteobacteria;Gammaproteobacteria;unclassified;unclassified;unclassified;
1230	0	0	1	Bacteria;Proteobacteria;Gammaproteobacteria;unclassified;unclassified;unclassified;
1231	0	0	1	Bacteria;Proteobacteria;Deltaproteobacteria;Desulfovibrionales;Desulfovibrionaceae;Desulfovibrio;
1232	0	0	1	Bacteria;Proteobacteria;Gammaproteobacteria;unclassified;unclassified;unclassified;
1233	0	1	0	Bacteria;Proteobacteria;Deltaproteobacteria;Desulfuromonadales;unclassified;unclassified;
1234	0	1	0	Bacteria;Bacteroidetes;Flavobacteria;Flavobacteriales;unclassified;unclassified;
1235	0	0	1	Bacteria;unclassified;unclassified;unclassified;unclassified;unclassified;
1236	0	1	0	Bacteria;Proteobacteria;Deltaproteobacteria;Desulfobacterales;unclassified;unclassified;
1237	0	0	1	Bacteria;unclassified;unclassified;unclassified;unclassified;unclassified;
1238	0	1	0	Bacteria;Proteobacteria;Deltaproteobacteria;Desulfobacterales;Desulfobacteraceae;unclassified;
1239	0	0	1	Bacteria;Proteobacteria;Alphaproteobacteria;Rhodobacterales;Rhodobacteraceae;unclassified;
1240	0	0	1	Bacteria;Proteobacteria;Deltaproteobacteria;Desulfobacterales;Desulfobacteraceae;Desulfosarcina;
1241	0	0	1	Bacteria;Proteobacteria;Gammaproteobacteria;unclassified;unclassified;unclassified;
1242	0	1	0	Bacteria;Proteobacteria;Epsilonproteobacteria;Campylobacterales;Helicobacteraceae;Sulfurovum;
1243	0	0	1	Bacteria;Proteobacteria;Deltaproteobacteria;unclassified;unclassified;unclassified;
1244	0	0	1	Bacteria;Proteobacteria;unclassified;unclassified;unclassified;unclassified;
1245	0	1	0	Bacteria;Proteobacteria;Deltaproteobacteria;Desulfobacterales;Desulfobulbaceae;Desulfobulbus;
1246	0	1	0	Bacteria;Firmicutes;unclassified;unclassified;unclassified;unclassified;
1247	0	1	0	Bacteria;unclassified;unclassified;unclassified;unclassified;unclassified;
1248	0	0	1	Bacteria;Proteobacteria;Gammaproteobacteria;Oceanospirillales;Oceanospirillaceae;unclassified;
1249	0	0	1	Bacteria;Acidobacteria;Acidobacteria;Acidobacteriales;Acidobacteriaceae;Gp9
1250	0	1	0	Bacteria;Proteobacteria;Gammaproteobacteria;unclassified;unclassified;unclassified;
1251	0	0	1	Bacteria;Bacteroidetes;Flavobacteria;Flavobacteriales;unclassified;unclassified;
1252	0	0	1	Bacteria;Bacteroidetes;unclassified;unclassified;unclassified;unclassified;
1253	0	1	0	Bacteria;Proteobacteria;Gammaproteobacteria;unclassified;unclassified;unclassified;
1254	0	0	1	Bacteria;Proteobacteria;Alphaproteobacteria;Rhodobacterales;Rhodobacteraceae;unclassified;
1255	0	1	0	Bacteria;Proteobacteria;Deltaproteobacteria;unclassified;unclassified;unclassified;

1256	0	0	1	Bacteria;Proteobacteria;Gammaproteobacteria;unclassified;unclassified;unclassified;
1257	0	0	1	Bacteria;Proteobacteria;Alphaproteobacteria;unclassified;unclassified;unclassified;
1258	0	1	0	Bacteria;Bacteroidetes;Sphingobacteria;Sphingobacteriales;unclassified;unclassified;
1259	0	0	1	Bacteria;Firmicutes;unclassified;unclassified;unclassified;unclassified;
1260	0	1	0	Bacteria;Firmicutes;Clostridia;Clostridiales;Lachnospiraceae;unclassified;
1261	0	1	0	Bacteria;Proteobacteria;unclassified;unclassified;unclassified;unclassified;
1262	0	0	1	Bacteria;Proteobacteria;Gammaproteobacteria;unclassified;unclassified;unclassified;
1263	0	1	0	Bacteria;Acidobacteria;Acidobacteria;Acidobacteriales;Acidobacteriaceae;Gp23
1264	0	0	1	Bacteria;Acidobacteria;Acidobacteria;Acidobacteriales;Acidobacteriaceae;Gp23
1265	0	0	1	Bacteria;Bacteroidetes;unclassified;unclassified;unclassified;unclassified;
1266	0	0	1	Bacteria;Bacteroidetes;unclassified;unclassified;unclassified;unclassified;
1267	0	0	1	Bacteria;Proteobacteria;Gammaproteobacteria;unclassified;unclassified;unclassified;
1268	0	0	1	Bacteria;Bacteroidetes;Sphingobacteria;Sphingobacteriales;Saprospiraceae;Haliscomenobacter;
1269	0	0	1	Bacteria;unclassified;unclassified;unclassified;unclassified;unclassified;
1270	0	0	1	Bacteria;Proteobacteria;Gammaproteobacteria;unclassified;unclassified;unclassified;
1271	0	0	1	Bacteria;unclassified;unclassified;unclassified;unclassified;unclassified;
1272	0	0	1	Bacteria;Proteobacteria;Alphaproteobacteria;unclassified;unclassified;unclassified;
1273	0	0	1	Bacteria;unclassified;unclassified;unclassified;unclassified;unclassified;
1274	0	0	1	Bacteria;Bacteroidetes;unclassified;unclassified;unclassified;unclassified;
1275	0	0	1	Bacteria;unclassified;unclassified;unclassified;unclassified;unclassified;
1276	0	0	1	Bacteria;Proteobacteria;Alphaproteobacteria;unclassified;unclassified;unclassified;
1277	0	1	0	Bacteria;Firmicutes;unclassified;unclassified;unclassified;unclassified;
1278	0	0	1	Bacteria;Bacteroidetes;Flavobacteria;Flavobacteriales;Cryomorphaceae;Crocinitomix;
1279	0	0	1	Bacteria;Proteobacteria;Alphaproteobacteria;unclassified;unclassified;unclassified;
1280	0	0	1	Bacteria;Proteobacteria;Deltaproteobacteria;Desulfobacterales;Desulfobacteraceae;unclassified;
1281	0	0	1	Bacteria;Bacteroidetes;unclassified;unclassified;unclassified;unclassified;
1282	0	0	1	Bacteria;Proteobacteria;Gammaproteobacteria;Alteromonadales;Colwelliaceae;Colwellia;
1283	0	0	1	Bacteria;Proteobacteria;Gammaproteobacteria;unclassified;unclassified;unclassified;
1284	0	0	1	Bacteria;Proteobacteria;unclassified;unclassified;unclassified;unclassified;
1285	0	0	1	Bacteria;Bacteroidetes;unclassified;unclassified;unclassified;unclassified;
1286	0	1	0	Bacteria;Bacteroidetes;unclassified;unclassified;unclassified;unclassified;
1287	0	0	1	Bacteria;Actinobacteria;Actinobacteria;Actinomycetales;Brevibacteriaceae;Brevibacterium;
1288	0	0	1	Bacteria;unclassified;unclassified;unclassified;unclassified;unclassified;
1289	0	0	1	Bacteria;Proteobacteria;Gammaproteobacteria;unclassified;unclassified;unclassified;
1290	0	1	0	Bacteria;Proteobacteria;unclassified;unclassified;unclassified;unclassified;
1291	0	0	1	Bacteria;Proteobacteria;Deltaproteobacteria;Desulfobacterales;Desulfobacteraceae;unclassified;
1292	0	1	0	Bacteria;unclassified;unclassified;unclassified;unclassified;unclassified;
1293	0	0	1	Bacteria;Proteobacteria;Gammaproteobacteria;unclassified;unclassified;unclassified;
1294	0	1	0	Bacteria;Proteobacteria;Alphaproteobacteria;Rhodobacterales;Rhodobacteraceae;unclassified;
1295	0	1	0	Bacteria;Fusobacteria;Fusobacteria;Fusobacteriales;Fusobacteriaceae;Fusobacterium;
1296	0	0	1	Bacteria;Proteobacteria;Deltaproteobacteria;Myxococcales;unclassified;unclassified;
1297	0	0	1	Bacteria;Proteobacteria;Gammaproteobacteria;unclassified;unclassified;unclassified;

1298	0	1	0	Bacteria;unclassified;unclassified;unclassified;unclassified;unclassified;
1299	0	0	1	Bacteria;Proteobacteria;unclassified;unclassified;unclassified;unclassified;
1300	0	1	0	Bacteria;Bacteroidetes;unclassified;unclassified;unclassified;unclassified;
1301	0	0	1	Bacteria;unclassified;unclassified;unclassified;unclassified;unclassified;
1302	0	1	0	Bacteria;unclassified;unclassified;unclassified;unclassified;unclassified;
1303	0	1	0	Bacteria;Bacteroidetes;unclassified;unclassified;unclassified;unclassified;
1304	0	1	0	Bacteria;unclassified;unclassified;unclassified;unclassified;unclassified;
1305	0	0	1	Bacteria;unclassified;unclassified;unclassified;unclassified;unclassified;
1306	0	0	1	Bacteria;Proteobacteria;Zetaproteobacteria;Mariprofundales;Mariprofundaceae;Mariprofundus;
1307	0	0	1	Bacteria;Proteobacteria;Gammaproteobacteria;unclassified;unclassified;unclassified;
1308	0	0	1	Bacteria;Proteobacteria;Betaproteobacteria;unclassified;unclassified;unclassified;
1309	0	0	1	Bacteria;Proteobacteria;Alphaproteobacteria;unclassified;unclassified;unclassified;
1310	0	0	1	Bacteria;Proteobacteria;Alphaproteobacteria;Rhodobacterales;Rhodobacteraceae;unclassified;
1311	0	1	0	Bacteria;Proteobacteria;unclassified;unclassified;unclassified;unclassified;
1312	0	0	1	Bacteria;Proteobacteria;Gammaproteobacteria;unclassified;unclassified;unclassified;
1313	0	0	1	Bacteria;Bacteroidetes;unclassified;unclassified;unclassified;unclassified;
1314	0	0	1	Bacteria;Proteobacteria;Gammaproteobacteria;unclassified;unclassified;unclassified;
1315	0	0	1	Bacteria;Acidobacteria;Acidobacteria;Acidobacteriales;Acidobacteriaceae;Gp23
1316	0	0	1	Bacteria;Chloroflexi;unclassified;unclassified;unclassified;unclassified;
1317	0	0	1	Bacteria;Bacteroidetes;unclassified;unclassified;unclassified;unclassified;
1318	0	0	1	Bacteria;unclassified;unclassified;unclassified;unclassified;unclassified;
1319	0	1	0	Bacteria;Proteobacteria;Deltaproteobacteria;unclassified;unclassified;unclassified;
1320	0	0	1	Bacteria;unclassified;unclassified;unclassified;unclassified;unclassified;
1321	0	1	0	Bacteria;Proteobacteria;Gammaproteobacteria;unclassified;unclassified;unclassified;
1322	0	0	1	Bacteria;unclassified;unclassified;unclassified;unclassified;unclassified;
1323	0	0	1	Bacteria;Bacteroidetes;unclassified;unclassified;unclassified;unclassified;
1324	0	0	1	Bacteria;Proteobacteria;Gammaproteobacteria;unclassified;unclassified;unclassified;
1325	0	1	0	Bacteria;Bacteroidetes;Sphingobacteria;Sphingobacteriales;Flammeovirgaceae;unclassified;
1326	0	1	0	Bacteria;unclassified;unclassified;unclassified;unclassified;unclassified;
1327	0	0	1	Bacteria;unclassified;unclassified;unclassified;unclassified;unclassified;
1328	0	1	0	Bacteria;Proteobacteria;Gammaproteobacteria;unclassified;unclassified;unclassified;
1329	0	0	1	Bacteria;Proteobacteria;Gammaproteobacteria;Alteromonadales;Idiomarinaceae;Idiomarina;
1330	0	0	1	Bacteria;unclassified;unclassified;unclassified;unclassified;unclassified;
1331	0	1	0	Bacteria;Bacteroidetes;unclassified;unclassified;unclassified;unclassified;
1332	0	0	1	Bacteria;Spirochaetes;Spirochaetes;Spirochaetales;Spirochaetaceae;Spirochaeta;
1333	0	1	0	Bacteria;Proteobacteria;unclassified;unclassified;unclassified;unclassified;
1334	0	0	1	Bacteria;unclassified;unclassified;unclassified;unclassified;unclassified;
1335	0	0	1	Bacteria;Acidobacteria;Acidobacteria;Acidobacteriales;Acidobacteriaceae;Gp22
1336	0	0	1	Bacteria;Proteobacteria;Gammaproteobacteria;Oceanospirillales;Alcanivoracaceae;Marinicella;
1337	0	0	1	Bacteria;Proteobacteria;Deltaproteobacteria;Desulfobacterales;Desulfobacteraceae;unclassified;
1338	0	1	0	Bacteria;unclassified;unclassified;unclassified;unclassified;unclassified;
1339	0	0	1	Bacteria;Proteobacteria;Deltaproteobacteria;Desulfobacterales;Desulfobacteraceae;unclassified;

1340	0	1	0	Bacteria;Proteobacteria;Gammaproteobacteria;unclassified;unclassified;unclassified;
1341	0	0	1	Bacteria;Proteobacteria;Alphaproteobacteria;unclassified;unclassified;unclassified;
1342	0	0	1	Bacteria;unclassified;unclassified;unclassified;unclassified;unclassified;
1343	0	0	1	Bacteria;Proteobacteria;Alphaproteobacteria;Rhodobacterales;Rhodobacteraceae;Roseovarius;
1344	0	0	1	Bacteria;Proteobacteria;unclassified;unclassified;unclassified;unclassified;
1345	0	1	0	Bacteria;Bacteroidetes;unclassified;unclassified;unclassified;unclassified;
1346	0	0	1	Bacteria;WS3;unclassified;unclassified;unclassified;unclassified;
1347	0	0	1	Bacteria;Bacteroidetes;unclassified;unclassified;unclassified;unclassified;
1348	0	0	1	Bacteria;Proteobacteria;Gammaproteobacteria;unclassified;unclassified;unclassified;
1349	0	1	0	Bacteria;Bacteroidetes;unclassified;unclassified;unclassified;unclassified;
1350	0	0	1	Bacteria;Actinobacteria;Actinobacteria;unclassified;unclassified;unclassified;
1351	0	0	1	Bacteria;Proteobacteria;Gammaproteobacteria;unclassified;unclassified;unclassified;
1352	0	0	1	Bacteria;Proteobacteria;unclassified;unclassified;unclassified;unclassified;
1353	0	1	0	Bacteria;Proteobacteria;Alphaproteobacteria;Rhizobiales;Aurantimonadaceae;Fulvimarina;
1354	0	1	0	Bacteria;Proteobacteria;Gammaproteobacteria;unclassified;unclassified;unclassified;
1355	0	0	1	Bacteria;Proteobacteria;Gammaproteobacteria;unclassified;unclassified;unclassified;
1356	0	1	0	Bacteria;Verrucomicrobia;Verrucomicrobiae;Verrucomicrobiales;unclassified;unclassified;
1357	0	1	0	Bacteria;TM7;unclassified;unclassified;unclassified;unclassified;
1358	0	0	1	Bacteria;Verrucomicrobia;Opitutae;Puniceicoccales;Puniceicoccaceae;unclassified;
1359	0	1	0	Bacteria;unclassified;unclassified;unclassified;unclassified;unclassified;
1360	0	0	1	Bacteria;Proteobacteria;Gammaproteobacteria;Salinisphaerales;Salinisphaeraceae;Salinisphaera;
1361	0	1	0	Bacteria;Bacteroidetes;Flavobacteria;Flavobacteriales;Cryomorphaceae;Owenweeksia;
1362	0	0	1	Bacteria;Proteobacteria;Gammaproteobacteria;unclassified;unclassified;unclassified;
1363	0	0	1	Bacteria;Acidobacteria;Acidobacteria;Acidobacteriales;Acidobacteriaceae;Gp22
1364	0	0	1	Bacteria;Bacteroidetes;Sphingobacteria;Sphingobacteriales;unclassified;unclassified;
1365	0	0	1	Bacteria;Proteobacteria;Gammaproteobacteria;unclassified;unclassified;unclassified;
1366	0	0	1	Bacteria;Proteobacteria;unclassified;unclassified;unclassified;unclassified;
1367	0	0	1	Bacteria;Bacteroidetes;unclassified;unclassified;unclassified;unclassified;
1368	0	1	0	Bacteria;Bacteroidetes;Flavobacteria;Flavobacteriales;Cryomorphaceae;unclassified;
1369	0	0	1	Bacteria;unclassified;unclassified;unclassified;unclassified;unclassified;
1370	0	0	1	Bacteria;Proteobacteria;Gammaproteobacteria;unclassified;unclassified;unclassified;
1371	0	0	1	Bacteria;Proteobacteria;Gammaproteobacteria;Thiotrichales;Thiotrichaceaea;Cocleimonas;
1372	0	1	0	Bacteria;unclassified;unclassified;unclassified;unclassified;unclassified;
1373	0	1	0	Bacteria;Bacteroidetes;Sphingobacteria;Sphingobacteriales;unclassified;unclassified;
1374	0	0	1	Bacteria;Proteobacteria;Deltaproteobacteria;Desulfobacterales;Desulfobacteraceae;unclassified;
1375	0	1	0	Bacteria;Proteobacteria;Deltaproteobacteria;Desulfobacterales;Desulfobacteraceae;Desulfobacterium;
1376	0	1	0	Bacteria;Proteobacteria;Deltaproteobacteria;Desulfobacterales;Desulfobacteraceae;unclassified;
1377	0	1	0	Bacteria;Proteobacteria;Gammaproteobacteria;unclassified;unclassified;unclassified;
1378	0	0	1	Bacteria;unclassified;unclassified;unclassified;unclassified;unclassified;
1379	0	1	0	Bacteria;Bacteroidetes;unclassified;unclassified;unclassified;unclassified;
1380	0	0	1	Bacteria;Proteobacteria;Deltaproteobacteria;Bdellovibrionales;Bacteriovoracaceae;Bacteriovorax;
1381	0	0	1	Bacteria;Proteobacteria;Gammaproteobacteria;unclassified;unclassified;unclassified;

1382	0	1	0	Bacteria;unclassified;unclassified;unclassified;unclassified;unclassified;
1383	0	0	1	Bacteria;Proteobacteria;Gammaproteobacteria;unclassified;unclassified;unclassified;
1384	0	1	0	Bacteria;Firmicutes;Clostridia;Clostridiales;unclassified;unclassified;
1385	0	1	0	Bacteria;Bacteroidetes;unclassified;unclassified;unclassified;unclassified;
1386	0	0	1	Bacteria;Proteobacteria;Gammaproteobacteria;Alteromonadales;Alteromonadaceae;Haliea;
1387	0	0	1	Bacteria;Proteobacteria;Deltaproteobacteria;Myxococcales;unclassified;unclassified;
1388	0	0	1	Bacteria;Proteobacteria;Alphaproteobacteria;Rhodobacterales;Rhodobacteraceae;unclassified;
1389	0	0	1	Bacteria;Bacteroidetes;unclassified;unclassified;unclassified;unclassified;
1390	0	1	0	Bacteria;Bacteroidetes;unclassified;unclassified;unclassified;unclassified;
1391	0	0	1	Bacteria;Bacteroidetes;unclassified;unclassified;unclassified;unclassified;
1392	0	0	1	Bacteria;Proteobacteria;Gammaproteobacteria;Alteromonadales;unclassified;unclassified;
1393	0	1	0	Bacteria;Proteobacteria;Gammaproteobacteria;unclassified;unclassified;unclassified;
1394	0	1	0	Bacteria;unclassified;unclassified;unclassified;unclassified;unclassified;
1395	0	0	1	Bacteria;Proteobacteria;Alphaproteobacteria;unclassified;unclassified;unclassified;
1396	0	0	1	Bacteria;Acidobacteria;Acidobacteria;Acidobacteriales;Acidobacteriaceae;Gp10
1397	0	1	0	Bacteria;Proteobacteria;Alphaproteobacteria;Rhodobacterales;Rhodobacteraceae;unclassified;
1398	0	0	1	Bacteria;Acidobacteria;Acidobacteria;Acidobacteriales;Acidobacteriaceae;Gp10
1399	0	1	0	Bacteria;Proteobacteria;Gammaproteobacteria;unclassified;unclassified;unclassified;
1400	0	0	1	Bacteria;Proteobacteria;unclassified;unclassified;unclassified;unclassified;
1401	0	0	1	Bacteria;unclassified;unclassified;unclassified;unclassified;unclassified;
1402	0	1	0	Bacteria;unclassified;unclassified;unclassified;unclassified;unclassified;
1403	0	1	0	Bacteria;TM7;unclassified;unclassified;unclassified;unclassified;
1404	0	1	0	Bacteria;Proteobacteria;Deltaproteobacteria;Desulfuromonadales;Desulfuromonadaceae;unclassified;
1405	0	0	1	Bacteria;Bacteroidetes;Flavobacteria;Flavobacteriales;Flavobacteriaceae;unclassified;
1406	0	0	1	Bacteria;Proteobacteria;Epsilonproteobacteria;Campylobacterales;Campylobacteraceae;unclassified;
1407	0	1	0	Bacteria;Proteobacteria;Deltaproteobacteria;Desulfobacterales;Desulfobulbaceae;Desulfobulbus;
1408	0	0	1	Bacteria;Bacteroidetes;unclassified;unclassified;unclassified;unclassified;
1409	0	1	0	Bacteria;Proteobacteria;Alphaproteobacteria;unclassified;unclassified;unclassified;
1410	0	1	0	Bacteria;Firmicutes;Clostridia;Clostridiales;Incertae_Sedis_XI;Dethiosulfatibacter;
1411	0	0	1	Bacteria;Proteobacteria;Gammaproteobacteria;unclassified;unclassified;unclassified;
1412	0	0	1	Bacteria;unclassified;unclassified;unclassified;unclassified;unclassified;
1413	0	1	0	Bacteria;Proteobacteria;Deltaproteobacteria;Desulfovibrionales;Desulfovibrionaceae;unclassified;
1414	0	1	0	Bacteria;Firmicutes;Clostridia;Clostridiales;Lachnospiraceae;unclassified;
1415	0	1	0	Bacteria;Bacteroidetes;Bacteroidia;Marinilabiliales;Marinifilaceae;Marinifilum;
1416	0	1	0	Bacteria;Proteobacteria;Deltaproteobacteria;Desulfuromonadales;Geobacteraceae;unclassified;
1417	0	0	1	Bacteria;Proteobacteria;unclassified;unclassified;unclassified;unclassified;
1418	0	1	0	Bacteria;Proteobacteria;Deltaproteobacteria;Desulfobacterales;Desulfobacteraceae;Desulfosarcina;
1419	0	0	1	Bacteria;Bacteroidetes;unclassified;unclassified;unclassified;unclassified;
1420	0	0	1	Bacteria;Proteobacteria;Alphaproteobacteria;Kordiimonadales;Kordiimonadaceae;Kordiimonas;
1421	0	0	1	Bacteria;Proteobacteria;Alphaproteobacteria;Rhodobacterales;Rhodobacteraceae;unclassified;
1422	0	1	0	Bacteria;Firmicutes;Clostridia;Clostridiales;unclassified;unclassified;
1423	0	0	1	Bacteria;Proteobacteria;Deltaproteobacteria;unclassified;unclassified;unclassified;

1424	0	1	0	Bacteria;unclassified;unclassified;unclassified;unclassified;unclassified;
1425	0	1	0	Bacteria;unclassified;unclassified;unclassified;unclassified;unclassified;
1426	0	0	1	Bacteria;Proteobacteria;Deltaproteobacteria;unclassified;unclassified;unclassified;
1427	0	1	0	Bacteria;Proteobacteria;Gammaproteobacteria;Thiotrichales;Thiotrichaceae;Cocleimonas;
1428	0	0	1	Bacteria;unclassified;unclassified;unclassified;unclassified;unclassified;
1429	0	0	1	Bacteria;Proteobacteria;Deltaproteobacteria;Desulfobacterales;Desulfobulbaceae;unclassified;
1430	0	0	1	Bacteria;Firmicutes;Clostridia;Clostridiales;unclassified;unclassified;
1431	0	0	1	Bacteria;Bacteroidetes;Flavobacteria;Flavobacteriales;Flavobacteriaceae;unclassified;
1432	0	0	1	Bacteria;unclassified;unclassified;unclassified;unclassified;unclassified;
1433	0	1	0	Bacteria;WS3;unclassified;unclassified;unclassified;unclassified;
1434	0	1	0	Bacteria;Bacteroidetes;unclassified;unclassified;unclassified;unclassified;
1435	0	0	1	Bacteria;Proteobacteria;Epsilonproteobacteria;Campylobacterales;Campylobacteraceae;Arcobacter;
1436	0	1	0	Bacteria;Bacteroidetes;Flavobacteria;Flavobacteriales;Flavobacteriaceae;Kordia;
1437	0	1	0	Bacteria;Proteobacteria;Alphaproteobacteria;Rhodobacterales;Rhodobacteraceae;unclassified;
1438	0	0	1	Bacteria;Proteobacteria;Alphaproteobacteria;Caulobacterales;Hyphomonadaceae;Hyphomonas;
1439	0	1	0	Bacteria;Proteobacteria;Deltaproteobacteria;unclassified;unclassified;unclassified;
1440	0	0	1	Bacteria;Proteobacteria;Alphaproteobacteria;unclassified;unclassified;unclassified;
1441	0	0	1	Bacteria;Actinobacteria;Actinobacteria;unclassified;unclassified;unclassified;
1442	0	1	0	Bacteria;Proteobacteria;Gammaproteobacteria;unclassified;unclassified;unclassified;
1443	0	1	0	Bacteria;Proteobacteria;Gammaproteobacteria;unclassified;unclassified;unclassified;
1444	0	0	1	Bacteria;unclassified;unclassified;unclassified;unclassified;unclassified;
1445	0	0	1	Bacteria;Bacteroidetes;unclassified;unclassified;unclassified;unclassified;
1446	0	0	1	Bacteria;Proteobacteria;Gammaproteobacteria;unclassified;unclassified;unclassified;
1447	0	1	0	Bacteria;Proteobacteria;Alphaproteobacteria;Rhodobacterales;Rhodobacteraceae;unclassified;
1448	0	0	1	Bacteria;Proteobacteria;unclassified;unclassified;unclassified;unclassified;
1449	0	0	1	Bacteria;unclassified;unclassified;unclassified;unclassified;unclassified;
1450	0	1	0	Bacteria;Firmicutes;Bacilli;Bacillales;unclassified;unclassified;
1451	0	0	1	Bacteria;Proteobacteria;Gammaproteobacteria;Alteromonadales;unclassified;unclassified;
1452	0	0	1	Bacteria;Proteobacteria;Gammaproteobacteria;unclassified;unclassified;unclassified;
1453	0	1	0	Bacteria;unclassified;unclassified;unclassified;unclassified;unclassified;
1454	0	0	1	Bacteria;unclassified;unclassified;unclassified;unclassified;unclassified;
1455	0	0	1	Bacteria;Proteobacteria;Epsilonproteobacteria;Campylobacterales;unclassified;unclassified;
1456	0	1	0	Bacteria;Proteobacteria;Alphaproteobacteria;Rhodobacterales;Rhodobacteraceae;unclassified;
1457	0	1	0	Bacteria;Acidobacteria;Acidobacteria;Acidobacteriales;Acidobacteriaceae;Gp10
1458	0	0	1	Bacteria;Proteobacteria;unclassified;unclassified;unclassified;unclassified;
1459	0	0	1	Bacteria;Bacteroidetes;Bacteroidia;Bacteroidales;Marinifilaceae;Marinifilum;
1460	0	0	1	Bacteria;Proteobacteria;Deltaproteobacteria;Myxococcales;Kofleriaceae;Kofleria;
1461	0	0	1	Bacteria;Proteobacteria;Deltaproteobacteria;Myxococcales;Polyangiaceae;unclassified;
1462	0	0	1	Bacteria;unclassified;unclassified;unclassified;unclassified;unclassified;
1463	0	0	1	Bacteria;unclassified;unclassified;unclassified;unclassified;unclassified;
1464	0	1	0	Bacteria;unclassified;unclassified;unclassified;unclassified;unclassified;
1465	0	0	1	Bacteria;Proteobacteria;Alphaproteobacteria;unclassified;unclassified;unclassified;

1466	0	0	1	Bacteria;Proteobacteria;Gammaproteobacteria;Pseudomonadales;Pseudomonadaceae;Pseudomonas;
1467	0	0	1	Bacteria;unclassified;unclassified;unclassified;unclassified;unclassified;
**Total**	**4344**	**3120**	**2844**	
